# Towards developing novel and sustainable molecular light-to-heat converters†

**DOI:** 10.1039/d1sc05077j

**Published:** 2021-10-18

**Authors:** Temitope T. Abiola, Benjamin Rioux, Josene M. Toldo, Jimmy Alarcan, Jack M. Woolley, Matthew A. P. Turner, Daniel J. L. Coxon, Mariana Telles do Casal, Cédric Peyrot, Matthieu M. Mention, Wybren J. Buma, Michael N. R. Ashfold, Albert Braeuning, Mario Barbatti, Vasilios G. Stavros, Florent Allais

**Affiliations:** Department of Chemistry, University of Warwick Gibbet Hill Road Coventry CV4 7AL UK v.stavros@warwick.ac.uk; URD Agro-Biotechnologies (ABI), CEBB, AgroParisTech 51110 Pomacle France florent.allais@agroparistech.fr; Aix Marseille Université, CNRS, ICR Marseille France josene-maria.toldo@univ-amu.fr; Department of Food Safety, German Federal Institute for Risk Assessment Max-Dohrn-Str. 8-10 10589 Berlin Germany Albert.Braeuning@bfr.bund.de; Department of Physics, University of Warwick Gibbet Hill Road Coventry CV4 7AL UK; EPSRC Centre for Doctoral Training in Diamond Science and Technology UK; Van ‘t Hoff Institute for Molecular Sciences, University of Amsterdam Amsterdam The Netherlands; Institute for Molecules and Materials, FELIX Laboratory, Radboud University 6525 ED Nijmegen The Netherlands; School of Chemistry, University of Bristol Cantock's Close Bristol BS8 1TS UK; Institut Universitaire de France 75231 Paris France

## Abstract

Light-to-heat conversion materials generate great interest due to their widespread applications, notable exemplars being solar energy harvesting and photoprotection. Another more recently identified potential application for such materials is in molecular heaters for agriculture, whose function is to protect crops from extreme cold weather and extend both the growing season and the geographic areas capable of supporting growth, all of which could help reduce food security challenges. To address this demand, a new series of phenolic-based barbituric absorbers of ultraviolet (UV) radiation has been designed and synthesised in a sustainable manner. The photophysics of these molecules has been studied in solution using femtosecond transient electronic and vibrational absorption spectroscopies, allied with computational simulations and their potential toxicity assessed by *in silico* studies. Following photoexcitation to the lowest singlet excited state, these barbituric absorbers repopulate the electronic ground state with high fidelity on an ultrafast time scale (within a few picoseconds). The energy relaxation pathway includes a twisted intramolecular charge-transfer state as the system evolves out of the Franck–Condon region, internal conversion to the ground electronic state, and subsequent vibrational cooling. These barbituric absorbers display promising light-to-heat conversion capabilities, are predicted to be non-toxic, and demand further study within neighbouring application-based fields.

## Abstract 2

The synthesis and photophysical properties of phenolic barbiturics are reported. These molecules convert absorbed ultraviolet light to heat with high fidelity and may be suitable for inclusion in foliar sprays to boost crop protection and production.

## Graphical abstract

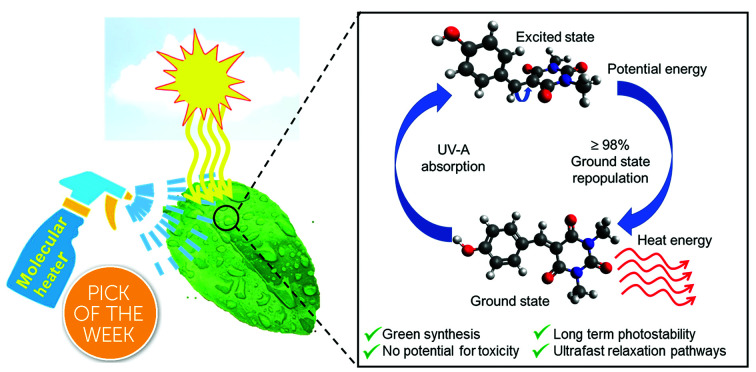

## Introduction

Photothermal (light-to-heat converting) materials are of paramount importance in many fields, including photonics, biomedical therapy, and photoprotection.^1–6^ Several materials, largely nano-based,^7–15^ have been designed and studied for photothermal applications. However, single-photon electronic excitation of small organic molecules in solution at 350 nm deposits nearly 4 eV into one solute molecule. Assuming efficient internal conversion and subsequent vibrational relaxation, most of this energy will be released as local heating of proximal solvent molecules. This offers a tantalising avenue for designing and applying a new class of photothermal materials. Here we focus on materials based on phenolic barbituric-acid derived molecules, which we henceforth term as barbiturics.

**Scheme 1:**
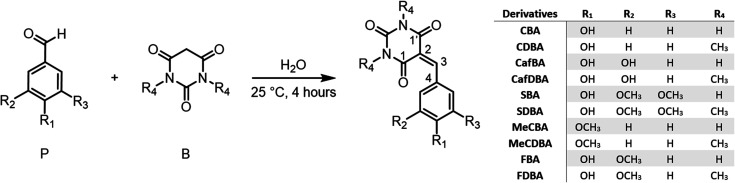
Synthesis of phenolic barbituric acid derivatives synthesis: coumaryl barbituric acid (CBA), coumaryl dimethyl barbituric acid (CDBA), caffeyl barbituric acid (CafBA), caffeyl dimethyl barbituric acid (CafDBA), sinapyl barbituric acid (SBA), sinapyl dimethyl barbituric acid (SDBA), 4-methoxy coumaryl barbituric acid (MeCBA), 4-methoxy coumaryl dimethyl barbituric acid (MeCDBA), ferulyl barbituric acid (FBA), and ferulyl dimethyl barbituric acid (FDBA). The central C atoms in the product are labelled consistently with the numbering scheme used in the electronic structure calculations.

Barbituric acid and its derivatives find widespread use as bio- and chemo-sensors for cell imaging and dye photosensitised polymerisation reactions.^16–24^ Molecules with the barbituric group (B in Scheme 1) show strong absorption localised in the UV-A (320–400 nm) region associated with excitation from the highest occupied molecular π orbital to the lowest unoccupied π* orbital, with molar extinction coefficients in the range of 30 000–40 000 M^−1^ cm^−1^.^20^ This absorption is significantly red-shifted relative to the corresponding π* ← π absorptions in smaller substituted phenols which, in terms of light-harvesting efficiency, conveys a significant advantage given that UV-A radiation is much more abundant at the Earth's surface than its UV-B counterpart (280–320 nm).^25^ Previous reports have shown that the photochemical properties of barbituric-acid derived molecules can be very sensitive to ring substituents.^16–21^ The barbituric absorbers featured here are formed by adding a phenolic substituent (P in Scheme 1) to the barbituric ring, which is a recognised means of promoting non-radiative decay pathways.^26–32^ In this way, the role of barbituric acid derivatives is switched from light-emitting to heat-emitting species, making them potential materials for photothermal applications.

The present work describes early endeavours towards a proof-of-concept to develop a new class of barbiturics that can be used as photothermal materials for agricultural applications, probably within a foliar spray. The foliar spray is envisioned containing light-to-heat converting species or ‘molecular heaters’, which would help protect plants from cold snaps that contribute to crop damage and food security challenges.^33–37^ Such an additional heat source should also allow some expansion of growth seasons and the geographic regions capable of supporting growth. To explore this further, we have: (1) applied green chemistry principles to the well-established Knoevenagel and Knoevenagel–Doebner condensation reactions of cinnamates^38–41^ to develop a synthetic route to barbiturics;^38,39,41–51^ (2) explored the photophysical properties of these barbiturics using steady-state and ultrafast transient absorption spectroscopies to gain insight into the light-to-heat generating pathways; (3) complemented these experiments with time-dependent density functional theory (TDDFT) and DFT/multireference configuration interaction (DFT/MRCI) calculations to describe these molecular relaxation pathways more fully; and (4) used *in silico* approaches to explore the potential toxicity of the barbiturics on humans.^52,53^

## Experimental and computational methods

### Synthesis and characterisation methods

Various benzaldehydes (P in Scheme 1) and barbituric or dimethyl barbituric acid were mixed in a round-bottom flask with H_2_O (100 g L^−1^) and stirred at room temperature for 4 hours. The precipitate was then filtered and freeze-dried overnight.

All reagents were purchased from Sigma-Aldrich, TCI, Merck or VWR and used as received. Solvents were purchased from Thermo Fisher Scientific and VWR. Deuterated dimethyl sulfoxide (DMSO-*d*_6_ <0.02% H_2_O) was purchased from Euriso-top. NMR analyses were recorded on a Bruker Fourier 300 spectrometer.

^1^H and ^13^C NMR spectra of samples were recorded in DMSO at, respectively, 300 MHz and 75 MHz. ^1^H chemical shifts are reported in parts per million defined relative to the solvent residual peak. Melting points were recorded using a Metler Toledo MP50 Melting Points system (*T*_initial_ = 150 °C, heating 3 °C min^−1^ until 299 °C with ME-18552 sample tubes) or calculated from differential scanning calorimetry measurements performed with a DSC Q20 system from TA Instruments. Typically, an ∼8 mg sample was placed in a sealed pan, flushed with high purity (99%) nitrogen gas, and passed through heat–cool–heat cycles at 10 °C min^−1^ in a temperature range of −50 °C to 200 °C. Thermal stability was assessed by thermogravimetric analysis (TGA) using a TA Q500 system from TA Instruments. Typically, ∼2 mg of each sample was equilibrated at 50 °C for 30 min and flushed with high purity nitrogen gas. All experiments were performed with a heating rate of 10 °C min^−1^ up to 500 °C. The reported *T*_d5%_ and *T*_d50%_ values represent the temperatures at which, respectively, 5% and 50% of the mass has been lost. Mass spectral analysis employed an Agilent Technologies 6545 Q-TOF LC/MS.

### Steady-state spectroscopy

Samples of each barbituric were prepared in both DMSO and dioxane (at concentration ∼30 μM) for long-term photostability studies. The UV-vis measurements were taken in a 1 cm path length quartz cuvette using a Cary 60 Spectrometer (Agilent Technologies) both before irradiation and at various times during 2 h irradiation with an arc lamp (Fluorolog 3, Horiba). The irradiance at maximal absorption (*λ*_max_) at the sample for each molecule was set so as to mimic one sun equivalent at the Earth's surface, with an 8 nm full width half maximum (FWHM). For the fluorescence emission measurements, samples were prepared to a concentration of ∼3 μM in dioxane and excited at the appropriate *λ*_max_ in a 1 cm pathlength quartz cuvette using the Horiba FluoroLog-3. Strong solvent emission precluded recording of similar fluorescence spectra for samples in DMSO.

^1^H NMR spectra of these sample solutions were taken before and after irradiation in an effort to identify any photoproducts formed.

### Transient absorption spectroscopy

#### (i) Transient electronic absorption spectroscopy (TEAS)

The femtosecond (fs) TEAS setup and procedure used to explore the photodynamics of the barbiturics has been detailed previously,^32,54–57^ and only information specific to the present experiment is reported here. Separate samples of CDBA, CBA, and MeCDBA, (coumaryl series) and of FBA and FDBA (ferulyl series) were prepared to 1 mM concentration in DMSO and dioxane (Fisher Chemicals). In all cases, the pump excitation wavelength was chosen to match the relevant *λ*_max_. The sample was delivered through a demountable Harrick Scientific flow-through cell equipped with two CaF_2_ windows separated by 100 μm polytetrafluoroethylene spacers, thereby defining the optical path length of the sample. The samples were circulated using a diaphragm pump (SIMDOS, KNF) recirculating from a 25 mL sample reservoir to ensure each pump–probe pulse sequence interacts with a fresh sample, with a maximum pump–probe delay of 2 ns.

#### (ii) Transient vibrational absorption spectroscopy (TVAS)

The TVAS setup and experimental technique employed in this work have also been reported in detail previously^32,58^ and, again, only information specific to the current experiments is reported. TVAS measurements of CBA and CDBA were undertaken in DMSO at 30 mM concentration, using the same sample delivery system as for the TEAS studies, but with a 150 μm thick spacer (to achieve a change in optical density ΔOD of ∼0.002) and a maximum pump–probe delay of 2.5 ns. In both cases, the UV pump pulse was centred at 385 nm, with a mid-IR probe pulse centred at 1529 cm^−1^. FBA and FDBA were not studied by TVAS because their solubility in DMSO was insufficient to achieve the required concentration. Fourier transform infrared (FTIR) spectra of each solution were taken using an FTIR spectrometer (VERTEX 70v, Bruker) under a nitrogen environment to remove vibrational features associated with atmospheric gases. The same sample holder used for the TVAS measurements was employed to record the FTIR spectra over a wavenumber range of 500–4000 cm^−1^ with a resolution of 1 cm^−1^.

### Electronic structure theory

The ground (S_0_) and first three singlet excited states (S_1_, S_2_, and S_3_) of the coumaryl and ferulyl series were optimised using DFT and TDDFT, respectively, with the ωB97XD functional.^59^ This functional was chosen due to the charge-transfer character of the S_1_ state.^60^ Yet, this functional shows a reasonable agreement with the *λ*_max_ absorption band for the barbiturics and has been reported to describe well the dynamics of similar compounds in a previous study. The optimisations were undertaken using the cc-pVDZ basis set, whereas the aug-cc-pVDZ basis set^61^ was used when calculating the vertical excitations, adiabatic energies and oscillator strengths. Solvent effects were included using the linear-response Polarisable Continuum Model (LR-PCM) with either DMSO or dioxane as implicit solvent.^62^

Additional B3LYP calculations using D3 dispersion correction,^63–65^ with a cc-pVDZ basis set, and PCM/DMSO were run to obtain geometries and frequencies/wavenumbers for ground state CBA and CDBA to help guide assignment of the ground state vibrational modes contributing to the measured FTIR spectra. Gaussian 16 rev a03 ^66^ was used for all (TD)DFT calculations.

The S_1_/S_0_ minimum energy crossing points (MECPs) were optimised using the penalty function method proposed by Martínez and co-workers,^67,68^ implemented in the Conical Intersection Optimizer (CIOpt) program, which we adapted to work with Gaussian software. These calculations were performed at the same level of theory as used for the (TD)DFT calculations. The topography of potential energy curves (PECs) was further characterised by calculating energies (at the ωB97XD/cc-pVDZ PCM/DMSO level) along with linear interpolations in internal coordinates (LIICs)^69^ linking the S_0_ minimum energy geometry, the S_1_ minimum energy geometry, and the S_1_/S_0_ MECP.

Absorption spectra of the various barbiturics in their respective S_1_ states were computed using the combined density functional theory and multireference configuration interaction (DFT/MRCI) method^70,71^ interfaced with Turbomole v.7.5.^72,73^ Twenty roots were computed using the S_1_ state density as a reference to obtain the transition dipole moments between the S_1_ and higher-lying excited states. Single point calculations at the DFT/MRCI level (in the gas phase) were also undertaken at each LIIC point along the S_1_ state PECs (hitherto described by TDDFT calculations).

The amount of excited-state charge-transfer character was evaluated by computing the charge-transfer index using the TheoDORE program,^74^ at the TDDFT level.

Further information regarding all these calculations is provided in ESI-J.†

### Antiradical activity

The radical scavenging activities of the barbituric derivatives were determined using the standard 2,2-diphenyl-1-picrylhydrazyl (DPPH) assay.^75^ This involved adding the barbituric derivative of interest in solution in ethanol to a homogeneous DPPH solution. These studies were performed under stirring for 7 h 25 min, with the following concentration scale: 400, 200, 100, 50, 25, and 12.5 μM. Every 5 min, the absorbance of the solution was measured at 520 nm. Curves showing percentage DPPH and reduced DPPH were plotted using Regressi® software, using an average of the last six points and the time taken to halve the initial DPPH free radical concentration, *i.e.*, EC_50_, determined from the crossing point of %DPPH and %reduced DPPH. The lower the EC_50_ value, the higher the antioxidant potential.

### *In silico* toxicology

#### (i) Mutagenicity and carcinogenicity

The mutagenic and carcinogenic properties of the barbiturics were investigated using three different software tools, as the predictive power of such approaches is proven to increase with the use of multiple models.^76,77^ Briefly, the different predictions obtained with the Toxicity Estimation Software Tool^78^ (TEST), the VEGA^79^ and LAZAR^80^ platforms were converted to a score value between 0 and 1, in which presumed non-mutagenicity/non-carcinogenicity falls in the range of 0–0.5, while mutagenicity/carcinogenicity ranges from 0.5 to 1. More detailed discussions and explanations of the generation of these score values and their meanings can be found in ESI-A.†

#### (ii) Endocrine toxicity

The VEGA platform was also used to investigate the endocrine toxicity of the barbiturics. The term “endocrine toxicity” here encompasses a total of 5 models, one that estimates receptor binding affinity (the estrogen receptor relative binding affinity model IRFMN) and four that predict receptor-mediated effects (estrogen receptor-mediated effect IRFMN/CERAPP, androgen receptor-mediated effect IRFMN/COMPARA, thyroid receptor alpha effect NRMEA, and thyroid receptor beta effect NRMEA). For each model, a qualitative prediction (yes/no) and information about the reliability of the prediction (low, moderate, or high reliability) are provided.

#### (iii) Acute and short-term toxicity

Acute and short-term toxicity were investigated using the *in silico* oral LD_50_ and the no-observed-adverse-effect level (NOAEL) after 90 days toxicity studies. The oral LD_50_ in rats was estimated by TEST based on a dataset comprising values from 7413 substances. The NOAEL was estimated using the module NOAEL – IRFMN/CORAL provided by the VEGA platform based on a dataset comprising values from repeated-dose 90 day oral toxicity studies in rodents with 140 substances.

#### (iv) Read-across approach

The read-across approach relies on structural similarities (termed chemical analogues) to predict, by extrapolation, the toxicity of compounds of interest. Using the OECD QSAR Toolbox,^81^ a list of structurally similar known compounds (barbiturate drugs in the present case) was selected and available experimental data relating to the endpoint's mutagenicity, carcinogenicity, and acute toxicity were extracted. The module ≪ read-across ≫ was then used to make predictions for the barbiturics of present interest.

## Results

### Synthesis

The barbiturics were synthesised using a green methodology based on the Knoevenagel condensation in water. The syntheses use no catalyst or organic solvent, thereby improving atom economy,^40,82^ and were performed at room temperature, thereby minimising energy consumption. Further, the barbiturics precipitated readily from the reaction medium, enabling their recovery and purification simply by a classic filtration, without recourse to energy- and solvent-consuming, waste-generating, and silica gel chromatography. Yields *via* this straightforward and sustainable synthetic method were in the range of 86–100%.

The synthesised barbiturics were fully characterised by ^1^H and ^13^C NMR spectrometry as well as high-resolution mass spectrometry (ESI-B and Fig. S1–S30†).

### Steady-state spectroscopy

**Fig. 1:**
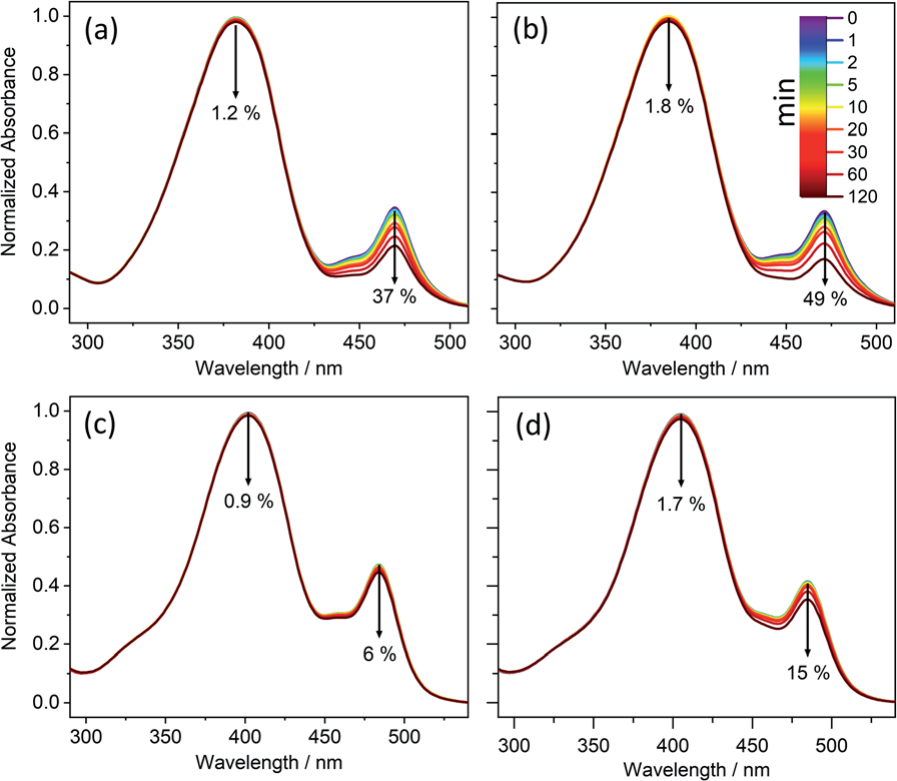
Long-term photostability of (a) CBA, (b) CDBA, (c) FBA, and (d) FDBA. UV-visible spectra of samples obtained in DMSO at varying duration of irradiation with a xenon arc lamp. The downward arrows denote the observed decrease in absorbance over 120 minutes of irradiation, with the time colour-coded.

UV-vis absorption spectra of the various barbiturics were measured in DMSO and dioxane. As shown in Fig. 1 and ESI Table S2,† each displays a broad absorption band with *λ*_max_ in the UV-A region. The electronic structure calculations (see later) associate this absorption with excitation to the first excited singlet (S_1_) state. Of the barbiturics investigated here, those with a single OH/OCH_3_ group in the R_1_-position (*i.e.*, CBA, CDBA, MeCBA and MeCDBA) exhibit the shortest *λ*_max_ (∼370–380 nm in both solvents). Introducing a second such group in the R_2_-position (as in FBA, CafBA, FDBA and CafDBA) causes a notable redshift in *λ*_max_ (to ∼410 nm), which is boosted further when a third such group is introduced in the R_3_-position (as in SBA and SDBA with *λ*_max_ ∼415 nm). The progressive addition of –OH/–OCH_3_ to the phenyl ring stabilises the ππ* state due to increased conjugation (ESI-J and Fig. S45† for detailed discussion).

In all cases, a second absorption feature is observable at *λ* ∼ 480 nm, which we attribute to the deprotonated phenolate anion. Given that this anion has no bearing on the ensuing discussion, it is not considered further here (though additional details and justification of this assignment can be found in the ESI-C and ESI-E, including Fig. S31 and S37†).

The photostability of these molecules was explored as described in the Experimental and computational methods. The results of these studies are reported in Fig. 1 and ESI-C.† They demonstrate the photostability of all the barbiturics, revealing only a small (<6%) reduction in sample absorbance at *λ*_max_ (ESI Table S2†).

^1^H NMR spectra of these solutions before- and after-irradiation show no observable difference (ESI-C and Fig. S32†), implying little or no photoproduct formation. Further irradiation experiments employing 10-fold reduced barbituric concentrations (*i.e.*, 1 mM) and with the irradiance increased to 7 sun equivalents still yielded no detectable photoproduct (by ^1^H NMR).

All the barbiturics were found to display good photostability, so only the coumaryl and ferulyl series were selected for further detailed study.

### Transient absorption spectroscopy

**Fig. 2:**
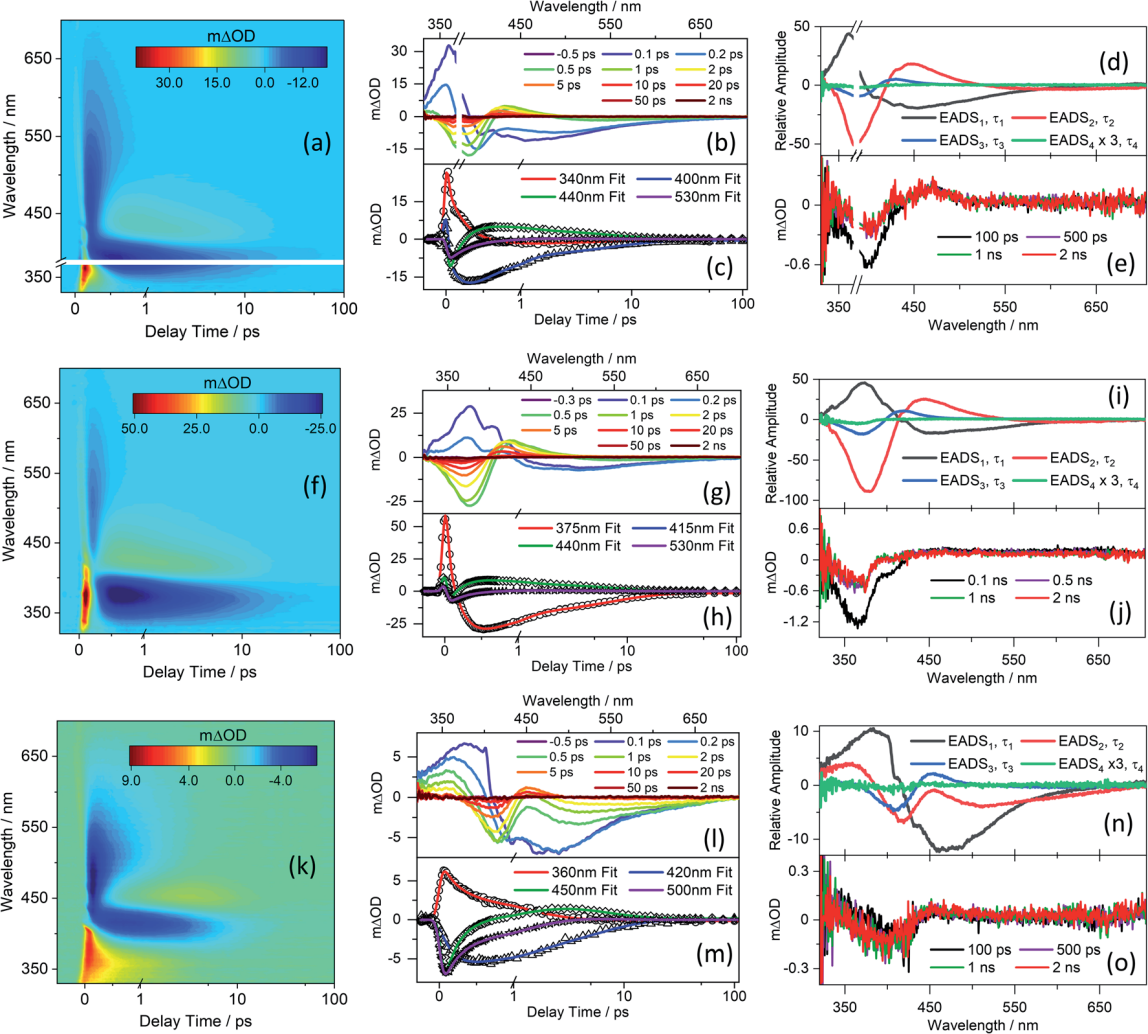
TEA spectra obtained for 1 mM of (a) CDBA/DMSO photoexcited at 385 nm, (f) MeCDBA/DMSO photoexcited at 375 nm, and (k) FDBA/DMSO photoexcited at 404 nm, shown as false colour maps. In each case, the pump–probe delay time is presented as a linear plot until 1 ps and then as a logarithmic scale between 1 and 100 ps. The same data are presented as line plots of *m*ΔOD *vs.* probe wavelength at selected pump–probe time delays in panels (b), (g), and (l) for CDBA/DMSO, MeCDBA/DMSO, and FDBA/DMSO, respectively. Panels (c), (h), and (m) show transients (raw data as symbols and fits as solid lines) at selected probe wavelengths for CDBA/DMSO, MeCDBA/DMSO, and FDBA/DMSO, respectively. The evolution associated difference spectra (EADS) produced by the fitting procedure are shown in panels (d) CDBA/DMSO, (i) MeCDBA/DMSO, and (n) FDBA/DMSO with, in each case, EADS_4_ multiplied by three as a visual aid. The transients at longer delay time (100 ps, 500 ps, 1 ns, and 2 ns) are shown in panels (e), (j) and (o) for CDBA/DMSO, MeCDBA/DMSO, and FDBA/DMSO, respectively. The smaller *m*ΔOD signal of the 2 ns transients observed for the ferulyl series as compared with the coumaryl series reflects the lower pump excitation power from our setup at the wavelength of interest.

TEA spectra measured following excitation of separate solutions of CDBA, MeCDBA, and FDBA in DMSO at the respective *λ*_max_ are presented in Fig. 2, while the corresponding spectra for CBA and FBA are consigned to the ESI-D and Fig. S33,† given their close similarities with those for the corresponding dimethyl substituted derivatives. Similar data obtained in dioxane and at higher barbituric concentrations in DMSO are reported in ESI Fig. S34–S36.† To verify the possible impact of the phenolate anion on the overall dynamics of the barbiturics, TEA spectra obtained following photoexcitation at 485 nm in the FDBA in DMSO sample are shown in ESI Fig. S37.†

Given the evident similarities between the TEA spectra measured when exciting all barbiturics at the appropriate *λ*_max_ values, in both DMSO and dioxane, the results are discussed as a collective. The spectra show four distinct features. The first is a strong negative feature which, in DMSO, is centred at ∼385 nm for CDBA and CBA, ∼375 nm for MeCDBA, and ∼405 nm for FDBA and FBA. Comparison with the UV-vis spectra reported in Fig. 1 and ESI Table S2† implies that this feature is attributable to the ground state bleach (GSB). A second negative feature evident at longer wavelengths is attributable to stimulated emission (SE) from the S_1_ state. This assignment is supported by the good match with the weak emission spectra observed following excitation of the various barbiturics at their respective *λ*_max_ (ESI-F and Fig. S38†). The SE feature is centred at ∼430 nm (∼460 nm) for the coumaryl (ferulyl) series and, in all cases, extends towards the red (∼650 nm) end of the TEA spectrum. The third is an intense positive feature peaking at ∼350 nm in all cases, attributable to excited-state absorption (ESA) (*i.e.*, absorption of S_1_ state molecules). This feature rapidly decays to zero within pump–probe delays of <500 fs (<3 ps) for the coumaryl (ferulyl) series. The decay timescale of this feature also matches the decay of the SE in all barbiturics. The slower decays of the ESA feature in TEA spectra of the ferulyl barbiturics compared to the coumaryl analogues are associated with the topography of the excited-state potential energy surface (PES) discussed later. Finally, the TEA spectra of all the barbiturics show a second ESA feature centred at ∼430 nm (∼450 nm) for the coumaryl (ferulyl) series, which decays on a similar timescale as the GSB. This second ESA feature is logically associated with vibrationally excited electronic ground state molecules formed following internal conversion (IC) from the S_1_ to S_0_ state.

Kinetic information was extracted from these TEA spectra by applying a global sequential 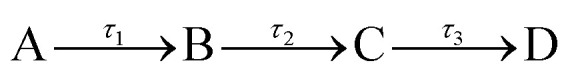 decay model, implemented through the Glotaran software package.^83,84^ The extracted time constants are reported in Table 1, while discussion of the evolution associated difference spectra (EADS) shown in Fig. 2d, l, and n is presented in ESI-D.† Note that the best fit includes a small, relatively featureless, and long-lived residual with an associated time constant (*τ*_4_) that persists well beyond the maximum available pump–probe delays. Note also that the quoted errors in Table 1 are those returned by the fitting software to 2σ, though the quality of the fits are better evaluated by inspecting the associated residuals reported in ESI-G and Fig. S39.† Where the error returned by the fitting package was shorter than the instrument response time, the error is quoted as half of the instrument response (as determined *via* the solvent-only transients presented in ESI-H and Fig. S40†).

**Table 1:** Time constants and associated errors extracted from fitting the TEA spectra collected for CBA, CDBA, MeCDBA, FBA, and FDBA in DMSO (top) and dioxane (bottom)

Solvent		CBA	CDBA	MeCDBA	FBA	FDBA
DMSO	*τ*_1_/fs	210 ± 40	230 ± 40	200 ± 40	170 ± 60	180 ± 60
	*τ*_2_/fs	390 ± 40	480 ± 40	440 ± 40	1050 ± 60	1130 ± 60
	*τ*_3_/ps	6.40 ± 0.10	6.80 ± 0.20	7.68 ± 0.10	6.52 ± 0.12	6.00 ± 0.10
	*τ*_4_/ns	>2	>2	>2	>2	>2
Dioxane	*τ*_1_/fs	200 ± 40	220 ± 40	210 ± 40	230 ± 40	270 ± 40
	*τ*_2_/fs	330 ± 40	380 ± 40	410 ± 40	710 ± 40	820 ± 40
	*τ*_3_/ps	6.82 ± 0.10	7.10 ± 0.10	8.10 ± 0.04	8.16 ± 0.13	8.43 ± 0.14
	*τ*_4_/ns	>2	>2	>2	>2	>2

The attribution of these time constants to specific photophysical processes is presented in tandem with guidance provided by the electronic structure calculations presented in the next section.

**Fig. 3:**
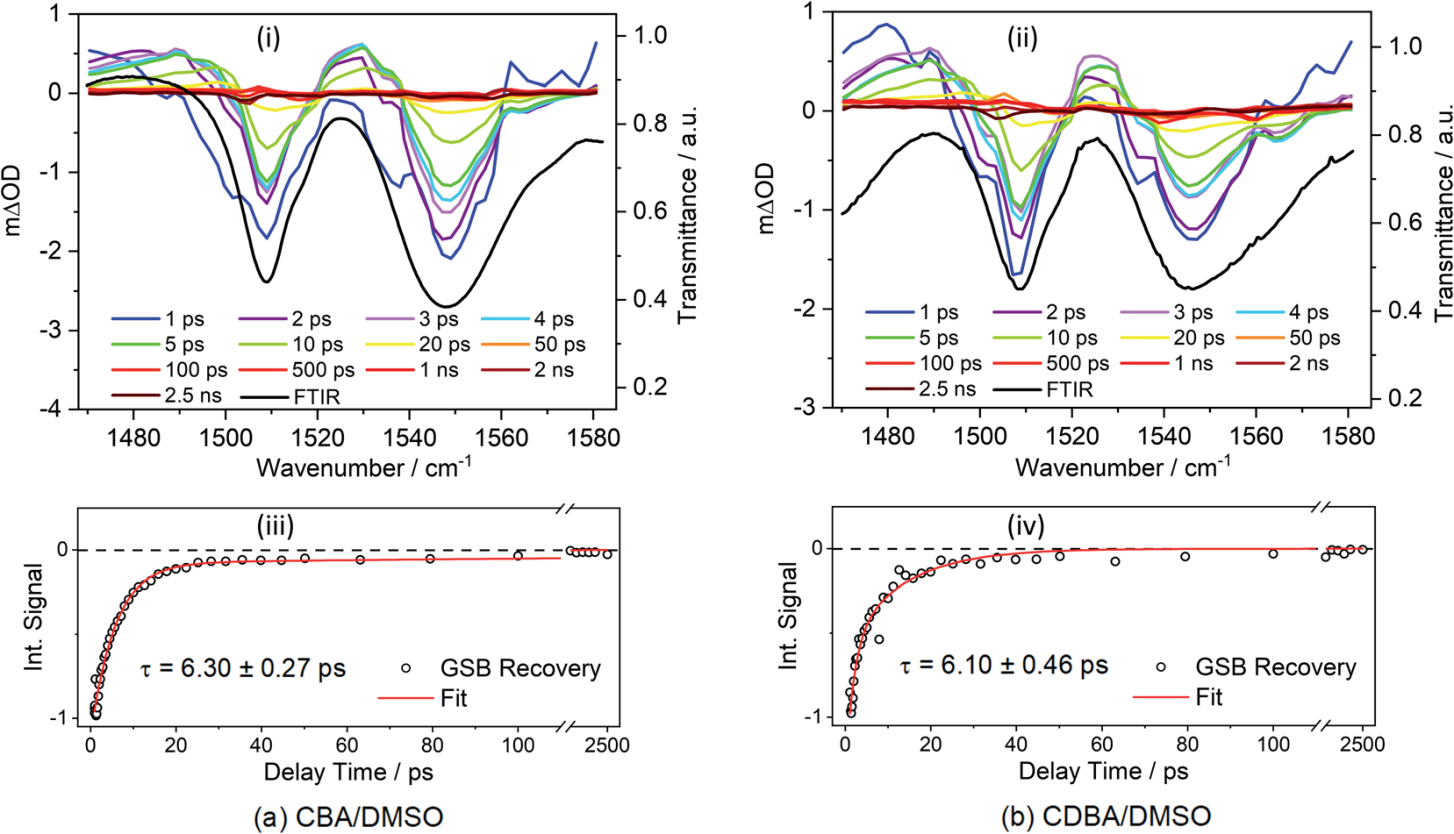
TVA spectra obtained for 30 mM solutions of (a) CBA/DMSO and (b) CDBA/DMSO, both photoexcited at 385 nm and using a broadband IR probe pulse centred at 1529 cm^−1^. The TVA spectra in the upper panels (i) and (ii) are presented as smoothed coloured line plots of *m*ΔOD (left-hand *y*-axis) *vs.* probe wavenumber at selected pump–probe delay times. The steady-state FTIR spectra are shown as black lines in the respective panels, with the transmittance scale shown on the right-hand *y*-axis. The lower panels (iii) and (iv) show the transients for the GSB recovery (raw data as open circles and fit as solid red line) of signals at selected wavenumber. The normalized integration of the GSB signal for CBA/DMSO (1539–1560 cm^−1^) and CDBA/DMSO (1534–1560 cm^−1^) were fitted with mono-exponential functions. In both cases, the delay times are plotted linearly until 110 ps; then there is a break until 1300 ps beyond which the 1300–2500 ps data are plotted on a logarithmic scale to show the full GSB recovery.

First, we report TVAS measurements following excitation of CBA and CDBA in DMSO at their respective *λ*_max_ values, which afford further insights into the UV-A induced dynamics of the barbiturics, most specifically the vibrational relaxation (*i.e.* photon energy to heat conversion) following IC from the S_1_ state to high vibrational levels of the S_0_ state. This part of the study began with recording FTIR spectra of CBA and CDBA in DMSO (ESI-I and Fig. S41†) and assigning the various features by comparison to the calculated S_0_ normal mode frequencies (wavenumbers) discussed below. TVA spectra for CBA and CDBA in DMSO in the probe window spanning 1470–1580 cm^−1^ are shown in Fig. 3 (additional TVA spectra are shown in ESI Fig. S42†), along with mono-exponential fits for the recovery of the respective GSB features centred at ∼1545 cm^−1^ which yield time constants of 6.30 ± 0.27 ps (CBA) and 6.10 ± 0.46 ps (CDBA). Similar fits of the GSB feature at ∼1507 cm^−1^ in both spectra are reported in ESI Table S4.† A global fitting procedure has not been employed here as the primary interest is just the recovery of S_0_ population (as revealed by the probed ground state vibrational mode). The exponential fit is started at the instant of maximal GSB signal intensity, *i.e.*, at a pump–probe delay of 0.9 ps in all cases, thereby avoiding any coherent artefacts at early time delays and effects attributable to perturbed-free induction decay, as reported previously,^85^ or solvent heating. Readers might also note that, within the available signal to noise and in contrast to the 2 ns transient of the TEA data, the GSB features in Fig. 3 appear to recover fully within the maximum pump–probe delay (2.5 ns).

The calculated S_0_ state vibrational frequencies (given in wavenumbers and shown in ESI-J i.†) suggest that the monitored GSB features for CBA and CDBA are associated with the allylic C=C stretch at ∼1545 cm^−1^ and the aromatic C–H in-plane bend at ∼1508 cm^−1^. Careful inspection of Fig. 3 reveals weaker ESA features also, most evident at ∼1490 and ∼1530 cm^−1^ in both CBA and CDBA, which might extend over the monitored GSB features. These absorptions could arise from several sources, including (i) photoexcited molecules in the S_1_ excited state, (ii) vibrationally ‘hot’ S_0_ molecules formed following IC from the S_1_ state, and (iii) photoproducts or intermediate species. The centre wavenumbers of these ESA features match reasonably with the predicted allylic C=C stretch and aromatic C–H in-plane bending motions in the S_1_ state. However, the fits to the TEAS data show that the S_1_ population decays back to the ground state within a few hundreds of fs, whereas the ESA features in the TVA spectra persist for several ps. The decay of these ESA features occurs on a similar timescale to that of the ESA features in the TEA spectra assigned to vibrational cooling of ‘hot’ S_0_ molecules (*i.e. τ*_3_ in Table 1), and it is tempting to attribute these ESA features in the TVA spectra accordingly.

### Electronic structure calculations

Quantum chemical calculations were undertaken to characterise the excited states of the barbiturics and provide further insights into their photodynamics. The ground and excited states of selected members of the coumaryl and ferulyl series were computed using (TD)DFT and implicit solvation (PCM/DMSO) (ESI-J ii, Fig. S43 and S44†). These molecules all have planar ground-state minimum energy geometries, with the rings coplanar to one another. However, the global minimum of the respective S_1_ states has a ∼90° twisted geometry (where the state has a twisted intramolecular charge-transfer character, henceforth labelled S_1-TICT_). Thus, following vertical excitation to the S_1_ state Franck–Condon (S_1-FC_), a considerable energy stabilisation is observed along the inter-ring twist coordinate of the respective S_1_ state potentials.

For the coumaryl derivatives, the present calculations find that the S_1-TICT_ geometry can be reached directly by optimising the S_1_ state starting from S_1-FC_. The situation with the ferulyl derivatives is more complicated. These molecules have two structural conformers, *syn* and *anti*, both containing an intramolecular hydrogen bond. The former is more stable (by 0.05 eV) in the ground state. From the perspective of the S_1_ PES, the main difference between them is that the *anti*-conformer has a locally excited, partially twisted minimum (S_1-LE_), with a C4–C3–C2–C1 dihedral angle of ∼25° (defined using the C atom numbering scheme shown in Scheme 1) located before reaching the global S_1-TICT_ minimum. No equivalent secondary minimum was located for the *syn*-conformer, but another (quasi-) stationary point associated with a coplanar geometry (S_1-pl_) was located. Nevertheless, this latter structure has an imaginary frequency that, although small, represents the out-of-plane movement that brings the molecule to the twisted geometry. The S_1_ state in both conformers has dominant locally excited (LE) character at the S_1-FC_ (and S_1-LE_ and S_1-pl_) geometries but strong charge-transfer (CT) character at the S_1-TICT_ minimum.

**Fig. 4:**
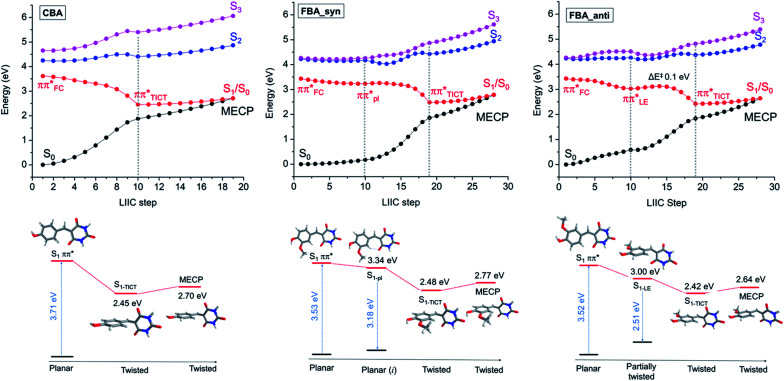
Potential energy curves calculated at the TD-ωB97XD/cc-pVDZ level of theory using PCM/DMSO for CBA, FBA_anti, and FBA_syn. For CBA: LIIC starting from the S_1_ geometry accessed by vertical excitation from the S_0_ state to the S_1-TICT_ min (shown up to the dashed grey line) and from S_1-TICT_ to S_1_/S_0_ MECP. For FBA: LIIC from the S_1_ geometry accessed by vertical excitation to the S_1-LE_ minimum or S_1-pl_ (shown up to the first dashed grey line), from the former point to the S_1-TICT_ (up to the second dashed grey line), and from S_1-TICT_ to the S_1_/S_0_ MECP. The FBA_*syn* isomer is 0.05 eV (1.1 kcal mol^−1^) more stable than FBA_*anti* in the ground state.

Vertical and adiabatic excitation energies for CBA and *syn*- and *anti*-FBA are shown in Fig. 4 and, for all other molecules investigated computationally, reported in ESI Table S6.† In all cases, photoexcitation populates the bright ^1^ππ* (S_1_) state, the S_1_ ← S_0_ transitions are predicted to have large (>0.75) oscillator strengths, and the S_2_ state is predicted to lie at least 0.5 eV above the S_1_ state. Natural Transition Orbitals (NTOs), which characterise the vertical excitations and the optimised excited states, are shown in the ESI Fig. S46–S49.† Inspection of the adiabatic excitation energies shows that the stabilization energy of S_1_ is considerably larger than of S_2_ and S_3_. As a result, the S_*n*_ − S_1_ (*n* = 2, 3) energy gap increases significantly upon geometry relaxation from the vertically excited geometry (ESI Table S6†). Along the linear interpolations in internal coordinate (LIIC) in Fig. 4, we can also see that the Sn state energies lie well above that of the S_1_ state, limiting the possibility of IC to a dark excited state, as has been proposed in previous studies of coumaric and ferulic cinnamate derivatives.^86,87^ Characterisation of the excited-state charge-transfer characters is reported in the ESI-J iii, Table S7, and Fig. S50.†

The geometries at the low-lying S_1_/S_0_ MECPs are similar to those at the respective S_1-TICT_ minima (ESI Fig. S43 and S44†), but the MECPs are consistently higher in energy (by ∼0.2–0.3 eV using PCM/DMSO). PECs for CBA and FBA following linear interpolations between the S_1_ vertically excited structure and the stationary points on the S_1_ state are shown in Fig. 4. The PECs for CDBA and FBDA are similar to those for CBA and FBA, respectively, as shown in ESI Fig. S51.† A common characteristic for all these molecules is that the pathway to the S_1_/S_0_ intersection is sloped.^88^ Relative to the respective S_1-TICT_ minima, the S_1_/S_0_ MECPs are higher in energy by 0.26 eV for CBA (and 0.32 eV for CDBA); by 0.22 eV for *anti*-FBA and *anti*-FDBA; and by 0.30 eV (0.38 eV) for *syn*-FBA (*syn*-FDBA). This suggests that any molecule photoexcited to the S_1_ PES that relaxes towards the S_1-TICT_ geometry will need to overcome a small energy barrier in order to undergo IC to the S_0_ state; some small fraction of the population may be (at least temporarily) trapped in the S_1-TICT_ minimum_,_ which might account for the (minor) long-lived component returned in the kinetic modelling of the TEAS data.

LIIC calculations also show that relaxation along the twisting coordinate from S_1-FC_ to S_1-TICT_ is barrierless for CBA (and CDBA). Similarly, for the *syn*-conformer of FBA (and FDBA), no barrier was found between the S_1-pl_ and S_1-TICT_ geometries. In the *anti*-conformer of FBA (and FDBA), however, the calculations return a small (0.1 eV) barrier along the LIIC connecting the S_1-LE_ and S_1-TICT_ minimum energy geometries. In all cases, the S_1_ potential-energy profile is relatively flat until reaching ∼65° twist angle. Since the S_1_/S_0_ energy gap is smaller than 1 eV at the S_1-TICT_ minimum energy geometry, we should not expect to observed visible fluorescence from molecules trapped in this well (whatever is the S_1_–S_0_ oscillator strength at this very twisted geometry). We propose that the weak fluorescence (<1% quantum yield) detected experimentally originates from molecules temporarily trapped in a very shallow S_1-LE_ minimum, which could be hidden in our calculations in the flat region before populating the S_1-TICT_ minimum.

### Rationalising the early time decay lifetimes

The early time decay kinetics revealed by the TEAS and TVAS measurements are now considered in the light of the quantum chemistry results. The ultrafast time constant (*τ*_1_) must relate to the photoexcited population evolving from S_1-FC_ towards the S_1-TICT_ minimum. This interpretation is consistent with the observed invariance of *τ*_1_ to the choice of solvent or the presence of the –OCH_3_ substituent in the ferulyl (*cf.* the coumaryl) barbiturics (Table 1).

*τ*_2_, in contrast, is longer in the ferulyl molecules than in the coumaryl series. It is also longer when FBA and FBDA are in DMSO than in dioxane. The influence of solvent polarity was investigated by additional TDDFT calculations for CBA and FBA using dioxane (*ε* = 2.2) as an implicit solvent instead of DMSO (*ε* = 46.8) (ESI Tables S8 and S9†). Changing the dielectric constant is found to have little effect on the vertical and adiabatic excitation energies of the locally-excited S_1_ state but has a greater impact on the S_1-TICT_ minimum and the S_1_/S_0_ MECP energies. The impact is method-specific, however. The LR/PCM calculations predict lower adiabatic energies at these geometries in dioxane than in DMSO. Such a result is surprising. The more polar TICT state (*cf.* the LE state) would generally be expected to be stabilised more by increasing the solvent polarity.^89^ Including one explicit DMSO molecule hydrogen-bonded to the hydroxyl group (shown in ESI Fig. S52†) does not change this result. The SS-PCM^90^ calculations for *syn*-FBA, however, do predict greater relative stabilisation of S_1-TICT_ and the S_1_/S_0_ MECP in DMSO than in dioxane (ESI-J v for discussion). More importantly, in the present context, all calculations return a smaller energy barrier to accessing S_1_/S_0_ MECP from S_1-TICT_ in dioxane than in DMSO (<0.1 eV in dioxane, *cf.* ∼0.2–0.4 eV in DMSO), consistent with the longer *τ*_2_ values obtained experimentally for the latter solvent and encouraging the view that *τ*_2_ reflects the timescale for IC to high vibrational levels of the S_0_ state through a non-adiabatic transition to the S_0_ state of the S_1_/S_0_ MECP.

DFT/MRCI calculated energies and oscillator strengths for transitions between the S_1_ state and the next 18 higher-lying singlet excited states of CBA and *syn*- and *anti*-FBA are presented in ESI Fig. S53 and S54† and discussed in ESI-J vi.† Briefly, the ESA centred at ∼350 nm plausibly samples S_1_ molecules evolving along the whole LIIC investigated, *i.e.* from S_1-FC_ through to the S_1_/S_0_ MECP.

### Antiradical activity

Antioxidants afford protection against reactive oxygen species (ROS) induced by UV,^91^ and it would clearly be beneficial for molecular heaters to exhibit both good photostability and antioxidant properties. Phenols are widely recognised as good antioxidant agents,^41,92^ so it was logical to investigate the antioxidant capacities of the phenolic barbiturics of current interest. By way of illustration, the radical scavenging ability of CafBA is described in ESI-K and Fig. S55.† Results for all the barbiturics are presented in ESI Table S10† and benchmarked against two families of commercially available antioxidants, butylhydroxyanisole (BHA) and butylhydroxytoluene (BHT), as well as the parent acids, *i.e.* barbituric acid (BA) and dimethyl barbituric acid (DBA). These four compounds all show EC_50_ values in the range of 2.8–4.2 nmol (recall, the lower the EC_50_ value the better protection against ROS generation). Amongst the barbiturics investigated here, those based on the caffeyl and sinapyl derivatives return similar EC_50_ values to those of the reference compounds (indeed the values for the caffeyl derivatives are superior to them), whereas those for the ferulyl and coumaryl are typically a few times higher.

### Mutagenicity, carcinogenicity, and endocrine toxicity

The predicted mutagenicity scores (ESI-L and Fig. S56a†) for all the barbiturics are <0.5 but above the 0.33 threshold value. On this basis, the barbiturics of current interest are predicted to be non-mutagenic, but the reliability of the prediction is not optimal. The carcinogenicity study returned scores that are between the thresholds discussed in ESI-A.† However, the low reliability of the prediction rendered the result inconclusive (ESI Fig. S56b†). None of the barbiturics was predicted to exert endocrine effects *via* estrogen or thyroid receptors (ESI Table S11†), but only CDBA, MeCDBA, and FDBA were predicted to have no binding affinity for estrogen receptors. All compounds were predicted active regarding the androgen receptor.

### Acute and short-term toxicity

The predicted acute and short-term oral LD_50_ and NOAEL values in rats are shown in ESI Table S12.† The LD_50_ values all fall in the range of 1600–2900 mg per kg body weight (bw), while the NOAEL values range between 8 and 120 mg per kg bw per day. These values suggest overall low toxicity for the barbiturics when compared with phenobarbital (PB), an approved anti-epileptic drug.

### Read-across approach

The read-across analysis was performed by drawing on data for five barbituric acids, including PB, listed in ESI Fig. S57.† Based on these reference data, the read-across approach predicts (ESI Table S13†) that none of the barbiturics of interest has the potential for acute toxicity, genotoxicity, or carcinogenicity.

## Discussion

The steady-state illumination and transient absorption measurements demonstrate that all barbiturics of current interest are photostable absorbers of UV-A/B radiation, both in strongly (DMSO) and weakly (dioxane) interacting solvents, confirming the suitability of these molecules as ‘molecular heaters’, by converting absorbed UV light into heat. Following initial photoexcitation to the S_1_ state, these molecules undergo efficient non-radiative transfer back to the S_0_ state, with minimal rival (and unwanted) photochemistry.

We now review the photodynamical processes associated with the extracted time constants (given in Table 1) and discuss the implications of these findings in the context of previous related studies.^55,93^ Photoexcitation results in the initial population of the S_1_ (^1^ππ*) state. These locally-excited S_1_ molecules evolve from the FC region towards a global minimum with a ∼90° twisted geometry (S_1-TICT_) and substantial charge-transfer character. This ultrafast dynamical process, described by a lifetime *τ*_1_, is near-independent of both the solvent and the presence of an additional methoxy substituent at position R_1_, R_2_ or R_3_ (see Scheme 1) of the benzene ring. As discussed above, this picture is corroborated by the TEA data, the TDDFT, and the DFT/MRCI calculations.

Population flowing into the S_1-TICT_ state decays with a longer time constant, *τ*_2_, to the ground state *via* the S_1_/S_0_ MECP. The quantum chemistry calculations show this MECP lying slightly above the S_1-TICT_ minimum, by an amount that depends both on the choice of solvent (the energy barrier is predicted to be larger in DMSO) and the presence of the –OMe group in the ferulyl but not the coumaryl derivatives. As discussed above, the experimental data lend support to these predictions. The *τ*_2_ values determined for the coumaryl derivatives are consistently smaller than those for the ferulyl species in both solvents; also, the *τ*_2_ values obtained for barbiturics in DMSO are greater than those in dioxane.

**Fig. 5:**
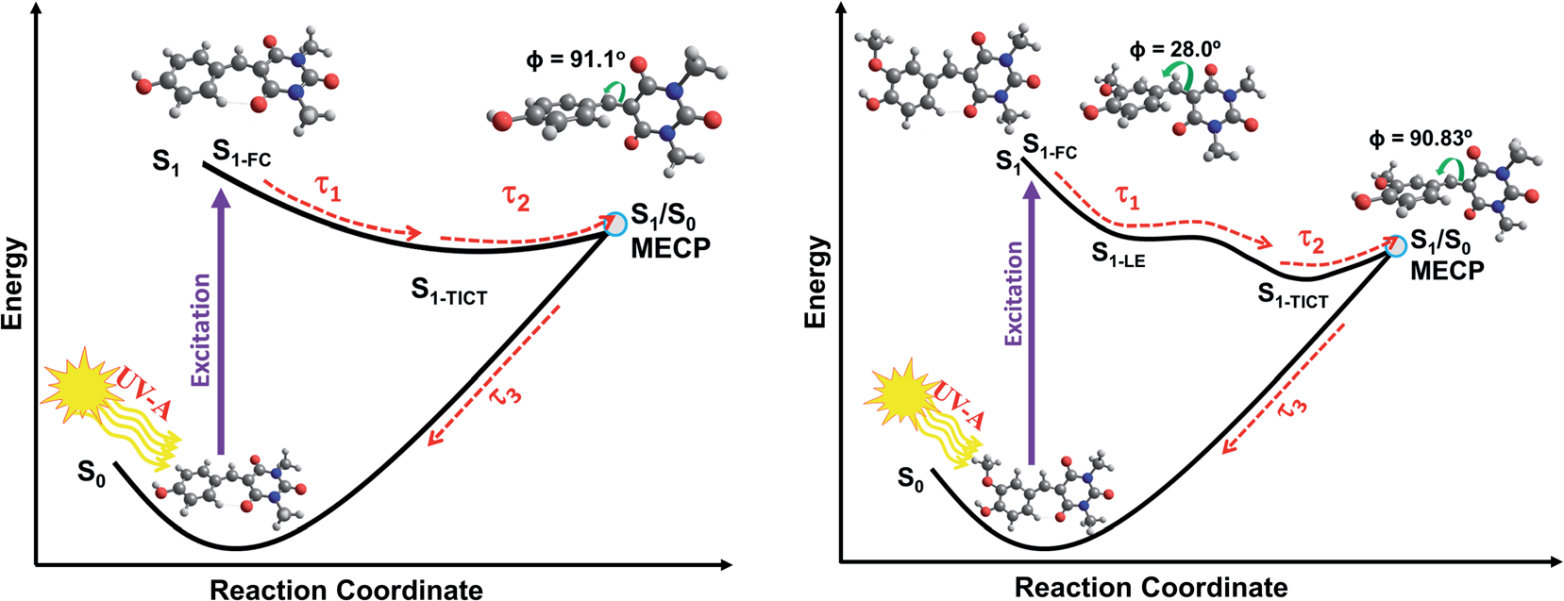
Schematic of the relaxation pathways for CDBA (left) and FDBA-*anti* (right) representing the coumaryl and ferulyl series, respectively. Molecular conformations at the Franck–Condon (FC) region, at the local S_1-LE_ minimum (only for FDBA-*anti*) and at S_1_/S_0_ MECP are shown. The respective *Φ* (C1–C2–C3–C4) dihedral angles, (see Scheme 1 for atom numbering), are also indicated. The molecular structures for the S_1-TICT_ configurations are omitted here for clarity but are shown in ESI Fig. S43 and S44† and are very similar to the respective S_1_/S_0_ MECP geometries.

IC yields vibrationally hot S_0_ molecules, which dissipate their excess energy to the surrounding solvent bath as heat through vibrational cooling.^30,94^ We associate this process with the time constant *τ*_3_. As reported elsewhere,^94–96^ the excess vibrational energy of the relaxing S_0_ species following IC manifests in the TEA spectra as absorption on the long wavelength side of the thermalised S_1_ ← S_0_ band (*i.e.* in the 430–470 nm range in the case of CBA) which progressively narrows and blue-shifts at longer time delays – as shown in all the TEA spectra in Fig. 2. The *τ*_3_ values extracted from data measured in DMSO are consistently slightly shorter than the corresponding quantities measured in dioxane, in keeping with prior expectations that interaction of solutes with a more strongly interacting (polar) solvent will promote a higher rate of vibrational energy transfer.^97^ The fits to the TVAS data for CBA and CDBA in DMSO (Fig. 3) return similar (∼6 ps) time constants for the GSB recovery, further validating the proposal that *τ*_3_ reports on the vibrational cooling of hot S_0_ molecules.^98,99^ Taken in their entirety, the present experimental and computational data support the view that the relaxation pathways following UV photoexcitation of the barbiturics are as shown schematically in Fig. 5.

Although the molecular structure of the barbiturics reported in the current work differs from the previously reported Meldrum's derivatives,^93,100^ the photophysics of both classes of molecules following UV excitation is largely similar. Hence, the prior literature lends further support to the present interpretation.

Table 1 listed a further time constant, *τ*_4_, included in the fitting process to accommodate the (small) incomplete recovery of the GSB signal observed in TEA spectra measured in both solvents. An additional ESA feature centred at ∼450 nm is also observable in the long time delay TEA spectra measured in DMSO, most visibly in the case of CDBA. (Fig. 2 and ESI Fig. S33–S36†). Note, we could not confirm similar incomplete GSB recovery in the TVA spectra recorded at long pump–probe delay times (Δ*t* = 2.5 ns) but give less weight to this finding, given the poor signal-to-noise ratio at such low signal strengths. The GSB recovery provides a measure of how fast (and how completely) the photoexcited population returns to the S_0_ state and indicates the closed-loop that repopulates the ground state through a reversal of the allylic bond twist after IC. Considering both the TEAS and TVAS data, we estimate (from the TEA spectra of the coumaryl and ferulyl series in DMSO by comparing the intensity of the residual absorbance in the 2 ns transients with that of the transient at the instant of maximal GSB signal) that the ‘incompleteness’ of the GSB recovery at Δ*t* = 2 ns may be ∼1% (∼2%) for coumaryl (ferulyl) series. The slightly higher incomplete recovery observed for the ferulyl species is in line with the electronic structure calculations, which predict an additional energy barrier on the PES connecting the S_1-LE_ to S_1-TICT_ geometries.

Based on the foregoing TDDFT and DFT/MRCI calculations and our inability to detect any stable photoproducts (*e.g.*, by ^1^H NMR following prolonged illumination, ESI-B†), the most straightforward attribution of the long-lived (>2 ns) component identified in the TEAS data is that some small fraction of the photoexcited population is (temporarily) trapped in the S_1-LE_ minimum. These S_1-LE_ molecules would display characteristic ESA spectra, but it is recognised that similar conclusions could be drawn if a small fraction of the photoexcited molecules were to undergo intersystem crossing to the lowest triplet (T_1_) state.

We now return to discuss the implications of the *in silico* toxicology studies. The predicted oral LD_50_ values for the barbiturics of interest are above the experimentally determined value for PB, a WHO model list of essential medicines with a similar molecular structure to the barbiturics (ESI Table S12†),^101^ but none (apart from MeCDBA) fall close to the strong acute toxicity threshold. The predicted NOAELs for the dimethyl-substituted barbiturics compare well with the 10 mg per kg bw per day NOAEL determined for PB in a two-year carcinogenicity study in male CD-1 mice.^102^ The present study suggests that the barbiturics have no mutagenic potential (one primary mechanism of genotoxicity), in line with previous studies on PB.^103^ The read-across analysis provides further encouragement, predicting negative genetic toxicity and negative carcinogenicity for all the barbituric derivatives, again consistent with the fact that PB is not considered carcinogenic for humans and with data from different epidemiological studies that have shown no clear evidence for an increased liver tumour risk.^104,105^ Finally, PB is inactive towards the androgen and estrogen receptors (based on experimental data used to build the datasets of the VEGA programs), leading to predictions that the barbiturics will not display endocrine toxicity. Thus, the *in silico* data reveal no critical potential for toxicity, especially genotoxicity, in any of the barbituric derivatives and encourage the view that these candidate compounds merit further development, testing (including *in vitro* and *in vivo* toxicological testing) and application as molecular heaters.

As a final aside, we note that most commercial foliar sprays are prepared in water. The barbiturics of present interest are not soluble in water, but we envisage formulations wherein the chosen barbituric is encapsulated in an oil phase and then applied as a suspension in water. This method, referred to as oil-in-water emulsions in agrochemical formulations, has been reported as safe and cost-effective.^106^ The present studies show that the photophysics of these barbiturics is largely insensitive to the solvent environment (polar or non-polar), and it is reasonable to anticipate that the light-to-heat conversion mechanism will not be significantly compromised by the formulation. The oil-in-water emulsion of these molecular heaters and further formulation developments are currently ongoing within our laboratories in collaboration with our academic and industrial collaborators.

## Conclusions

This work has developed a new series of phenolic barbituric acid derivatives with potential application as photothermal materials. An important attribute of these barbiturics is the red-shifted UV-absorption into the UV-A region, in contrast to the many similar organic filter molecules studied previously, whose absorption falls at UV-B and shorter wavelengths.^27,30,54,55,93^ This offers a critical advantage since UV-A is relatively abundant at the Earth surface, promoting barbiturics in solar photothermal applications.

The barbiturics have been synthesised through catalyst- and organic solvent-free Knoevenagel condensation. This approach offers a sustainable and environmentally friendly synthetic route of barbiturics, which could be extended to other similar systems. The photophysics of these barbiturics following absorption of UV radiation has been studied through ultrafast transient absorption spectroscopies, excited-state calculations, and steady-state studies. The UV-visible absorption spectra, photostability, and NMR studies show that these molecules absorb UV radiation efficiently, without photoproduct formation over two hours of sun-like irradiation. The ultrafast studies demonstrate that, after photoexcitation, these barbiturics return to the ground state on an ultrafast (picosecond) timescale with an impressive (>98%) cycling efficiency. The relaxation mechanism has been determined to be initiated by excited state distortion to a TICT state, followed by S_1_/S_0_ IC, giving rise to vibrationally hot electronic ground state molecules which transfer vibrational energy to the solvent environment. Therefore, the primary deactivation mechanism is converting the absorbed UV photon energy into vibrational energy, which is transferred to the immediate environment as heat, thereby making this series of molecules suitable for applications where efficient light-to-heat conversion material is required. Furthermore, *in silico* data have shown that these barbiturics do not show any critical potential for toxicity or genotoxicity.

Considering their green synthesis, impressive photostability, and lack of critical toxicity, the phenolic barbituric acid derivatives introduced in this work are promising molecular heaters for applications in agriculture (particularly when incorporated in a foliar spray to promote crop heating), phototherapy, photoimaging, and generally where photothermal conversion is desirable.

## Data availability

The datasets presented in this study can be found in online repositories. The names of the repository/repositories and accession number(s) can be found below: Zenodo repository DOI: 10.5281/zenodo.5564384.

## Author contributions

T. T. A., J. M. W. and D. C. acquired and analysed the transient absorption data. T. T. A. acquired and analysed the steady-state spectroscopy data. J. M. T. conceived and conducted the computational studies. J. M. T., M. T do C. and M. B. interpreted the computational data. B. R., C. P., M. M. M. and F. A. performed the synthesis, characterisation, and antiradical analysis. J. A. and A. B. conducted and analysed the *in silico* toxicology studies. M. N. R. A., W. J. B. and V. G. S. conceived the spectroscopy experiments and guided the accompanying data analysis and interpretation. All authors contributed to writing the manuscript.

## Conflicts of interest

There are no conflicts to declare.

## Supplementary Material

SC-012-D1SC05077J-s001
