# Supplementary material for: Towards developing novel and sustainable molecular light-to-heat converters
Source: Chem Sci. 2021 Oct 18;12(46):15239–52. doi: 10.1039/d1sc05077j (PMC8634993; doi:10.1039/d1sc05077j)
Supplement: SC-012-D1SC05077J-s001 [file SC-012-D1SC05077J-s001.pdf]

Electronic Supplementary Material (ESI) for Chemical Science.
This journal is © The Royal Society of Chemistry 2021

ELECTRONIC SUPPLEMENTARY INFORMATION

Towards Developing Novel and Sustainable Molecular Light-to-Heat Converters

Temitope T. Abiola,[a,#] Benjamin Rioux,[b,#] Josene M. Toldo,[*,c,#] Jimmy Alarcan,[d,#] Jack M. Woolley,[a] Matthew A.P. Turner,[a,e] Daniel J. L. Coxon,[a,e,f] Mariana T. do Casal,[c] Cédric Peyrot,[b] Matthieu M. Mention,[b] Wybren J. Buma,[g,h] Michael N.R. Ashfold,[i] Albert Braeuning,[*,d] Mario Barbatti,[c,j] Vasilios G. Stavros[*,a] and Florent Allais[*,b]

a. Department of Chemistry, University of Warwick, Gibbet Hill Road, Coventry, CV4 7AL.

Email: v.stavros@warwick.ac.uk

b. URD Agro-Biotechnologies (ABI), CEBB, AgroParisTech, 51110, Pomacle, France.

Email: florent.allais@agroparistech.fr

c. Aix Marseille Université, CNRS, ICR, Marseille, France.

Email: josene-maria.TOLDO@univ-amu.fr

d. Department of Food Safety, German Federal Institute for Risk Assessment, Max-Dohrn-Str. 8-10, 10589, Berlin, Germany. Email: Albert.Braeuning@bfr.bund.de

e. Department of Physics, University of Warwick, Gibbet Hill Road, Coventry, CV4 7AL, United Kindom.

f. EPSRC Centre for Doctoral Training in Diamond Science and Technology, United Kingdom.

g. Van 't Hoff Institute for Molecular Sciences, University of Amsterdam, Amsterdam, The Netherlands.

h. Institute for Molecules and Materials, FELIX Laboratory, Radboud University, 6525 ED Nijmegen, The Netherlands.

i. School of Chemistry, University of Bristol, Cantock's Close, Bristol, BS8 1TS, United Kingdom.

j. Institut Universitaire de France, 75231 Paris, France.

# These authors contributed equally to this work.

Table of Contents

A. *In-Silico* toxicology methods. 3

B. Characterisation of the barbituric derivatives 5

i. Barbituric acid series 5

ii. Dimethyl barbituric acid series: 13

C. Additional steady-state measurements. 21

D. Additional TEAS measurements. 24

E. TEA spectra of FDBA in DMSO photoexcited at 485 nm. 28

F. Fluorescence emission spectra. 30

G. Residuals for the sequential fit to the TEA spectra. 31

H. Instrument response. 32

I. FTIR measurements and additional TVAS data 33

J. Computational studies 35

i. Wavenumber calculations for assigning the FTIR spectra 35

ii. Geometries and energies 37

iii. Characterisation of the states in terms of charge transfer character 41

iv. Linear interpolations 43

v. Solvent effects 43

vi. DFT/MRCI calculations 45

K. Antiradical activities 48

L. Additional *in silico* toxicology prediction results 49

M. References 51

A. *In-Silico* toxicology methods.

In silico analysis was performed using three different software tools.

The Toxicity Estimation Software Tool (TEST) was employed for mutagenicity prediction. The output value for the "Consensus Method" was used for the present study, which is the mean of the two values obtained for the "Hierarchical Clustering" and the "Nearest Neighbor" methods. TEST gives a prediction in the form of a numeric value between 0 and 1, in which presumable non-mutagenicity ranges from 0 to 0.50, while mutagenicity ranges from 0.51 to 1.

In addition to the TEST platform, we have also employed the VEGA platform, which utilises four models for predicting mutagenicity and carcinogenicity, together with one mutagenicity consensus model. The mutagenicity endpoint models (i.e. ISS, SARpy, CAESAR, Mutagenicity Read-Across/KNN) were developed based on experimental data derived from *in vitro* studies (e.g. the Ames Test in *Salmonella typhimurium* strains). In contrast, the carcinogenicity models (i.e. ISS, ISSCAN-CGX, CAESAR, ANTARES) were built using *in vivo* data from studies in different species (mainly mice and rats). The VEGA models give a prediction with a "yes" or "no" statement, together with information on the reliability of the prediction (low, moderate, or high reliability). To allow comparison with results from other methods, the wording of the VEGA predictions was translated into a so-called mutagenicity or carcinogenicity score ranging between 0 and 1. In this case, 0 stands for non-mutagenic/non-carcinogenic and 1 stands for strongly mutagenic/carcinogenic. The classification for the graded translation is given in Table S1. The mutagenicity consensus model gives a score value ranging from 0 to 1. In cases where the test compound is predicted as non-mutagenic, the given value must then be re-scaled to fit the graduation described in Table S1. Hence, non-mutagenicity values were re-scaled using the following calculation: $\boldsymbol{score} = \frac{(\mathbf{1}-\boldsymbol{x}) \times \mathbf{0.5}}{\mathbf{0.9}}$, where $\boldsymbol{x}$ is the value given by VEGA for the mutagenicity consensus model. In cases where the test compound is predicted as mutagenic, the given value is re-scaled using the following calculation: $\boldsymbol{score} = \mathbf{1} - \frac{(\mathbf{1}-\boldsymbol{x}) \times \mathbf{0.5}}{\mathbf{0.9}}$, where $\boldsymbol{x}$ is again the value given by VEGA for the mutagenicity consensus model.

Table S1. Translation of the VEGA predictions into a mutagenicity/carcinogenicity score

Prediction	Reliability	Score
mutagenic/carcinogenic	experimental data	1
mutagenic/carcinogenic	good reliability	0.9
possible mutagenic/carcinogenic	good reliability	0.8
mutagenic/carcinogenic	moderate reliability	0.7
possible mutagenic/carcinogenic	moderate reliability	0.6
(possible) mutagenic/carcinogenic	low reliability	0.5
(possible) non-mutagenic/non-carcinogenic	low reliability	0.5
possible non-mutagenic/non-carcinogenic	moderate reliability	0.4
non-mutagenic/non-carcinogenic	moderate reliability	0.3
possible non-mutagenic/non-carcinogenic	good reliability	0.2
non-mutagenic/non-carcinogenic	good reliability	0.1
non-mutagenic/non-carcinogenic	experimental data	0

The open-source software LAZAR includes the three models for carcinogenicity prediction (“Rat”, “Mouse”, and “Rodents (multiple species/sites)”), and one model for mutagenicity prediction (“*Salmonella typhimurium*”). The model gives a prediction in the form of an “active/non-active” statement, combined with a probability score. The predictions of the three LAZAR carcinogenicity models were combined and translated into a single carcinogenicity score as follows: Starting from a virtual value of 0.5, a value of 0.133 was added for every model that predicted “carcinogenic”, while 0.133 was subtracted for every model yielding “non-carcinogenic”. Thus, in the case that all three models predicted a compound to be carcinogenic, the final value would be 0.9, and in the case that all three models yielded “non-carcinogenic” for a given compound, the final value would be 0.1. For mutagenicity prediction, the given probability was translated into a score as follows: A probability value higher than 0.66 was considered equal to the “good reliability” of the VEGA predictions and is attributed to the score of 0.1 or 0.9 depending on the negative or positive prediction. In contrast, a probability value lower than 0.33 was considered equal to the “low reliability” of the VEGA predictions and is attributed to the score of 0.5. However, a probability value between 0.33–0.66 was considered equal to the “moderate reliability” of the VEGA predictions and is attributed the score of 0.3 or 0.7 depending on the negative or positive prediction. To achieve a single mutagenicity/carcinogenicity score, the arithmetic mean of the different generated prediction scores was calculated and interpreted as follows: A score >0.66 means a positive prediction with good reliability; a score <0.33 means a negative prediction with good reliability; scores between 0.33 and 0.66 are considered equivocal, i.e. scores in the ranges 0.33-0.5 and 0.5-0.66 are regarded as, respectivelty, negative and positive predictions but with insufficient reliability.

B. Characterisation of the barbituric derivatives

i. Barbituric acid series

Coumaryl barbituric acid (CBA): filtration led to a yellow powder (91%); m.p. 139 °C; UV: λ_{max} (EtOH, nm) 385, ε (L.mol^{-1}.cm^{-1}) 27700.

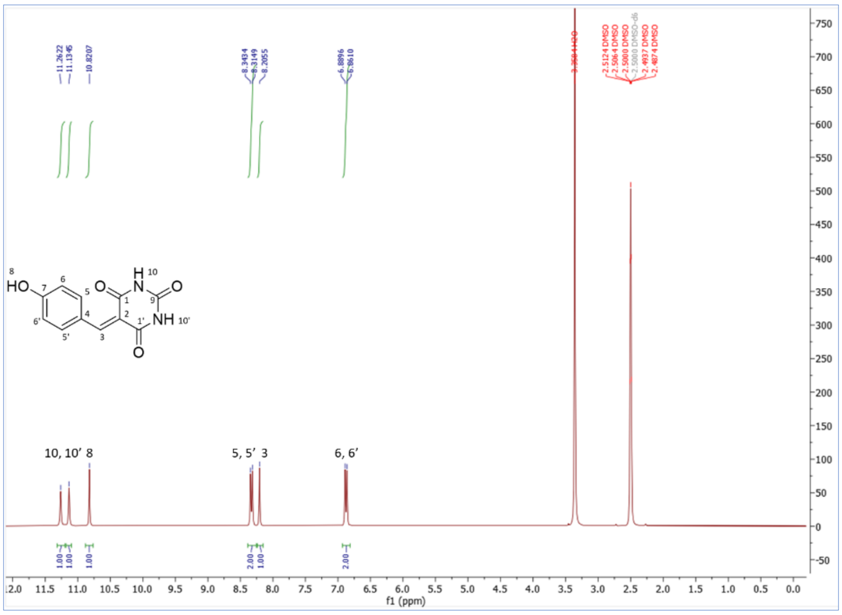

Fig. S1: ^{1}H NMR (300 MHz, 25 °C, DMSO-d$_6$) δ: 11.26 and 11.13 (2H, 2s, H-10 and H-10'), 10.82 (1H, s, H-8), 8.33 (2H, d, *J* = 8.6 Hz, H-5 and H-5'), 8.21 (1H, s, H-3), 6.88 (2H, d, *J* = 8.6 Hz, H-6 and H-6').

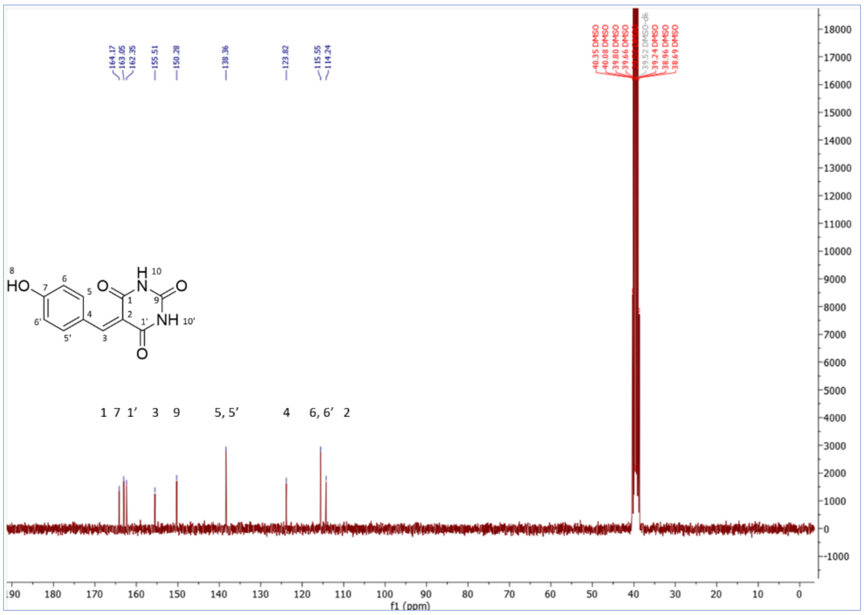

Fig. S2: ^{13}C NMR (75 MHz, 25 °C, DMSO-d$_6$) δ: 164.2 (C-1), 163.1 (C-7), 162.4 (C-1'), 155.5 (C-3), 150.3 (C-9), 138.4 (C-5 and C-5'), 123.8 (C-4), 115.6 (C-6 and C-6'), 114.2 (C-2).

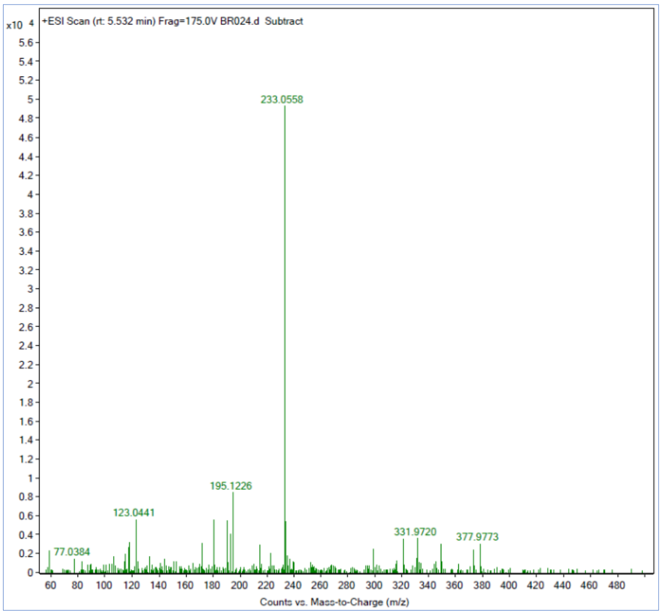

Fig. S3: TOF MS ES+: $[M+H]^+$ for $C_{11}H_9N_2O_4$: *m/z* 233.0562; found: *m/z* 233.0558.

Ferulyl barbituric acid (FBA): filtration led to a yellow orange powder (88%); m.p. not-determined; UV: λ_{max} (EtOH, nm) 405, ε ($L.mol^{-1}.cm^{-1}$) 33500.

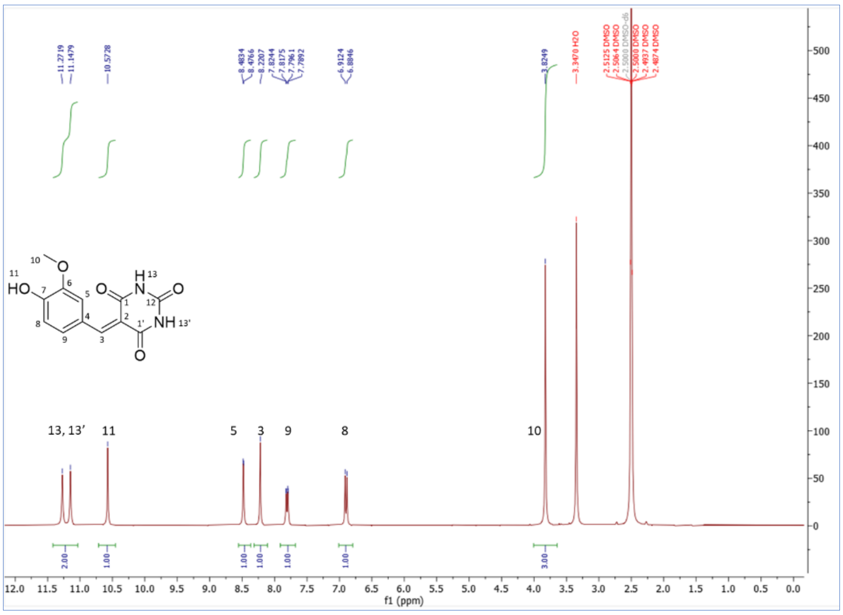

Fig. S4: ^1H NMR (300 MHz, 25 °C, DMSO-d_6) δ: 11.27 and 11.15 (2H, 2s, H-13 and H-13'), 10.57 (1H, s, H-11), 8.48 (1H, d, *J* = 2.0 Hz, H-5), 8.22 (1H, s, H-3), 7.81 (1H, dd, *J* = 2.0 Hz and *J* = 8.5 Hz, H-9), 6.90 (1H, d, *J* = 8.3 Hz, H-8), 3.82 (3H, s, H-10).

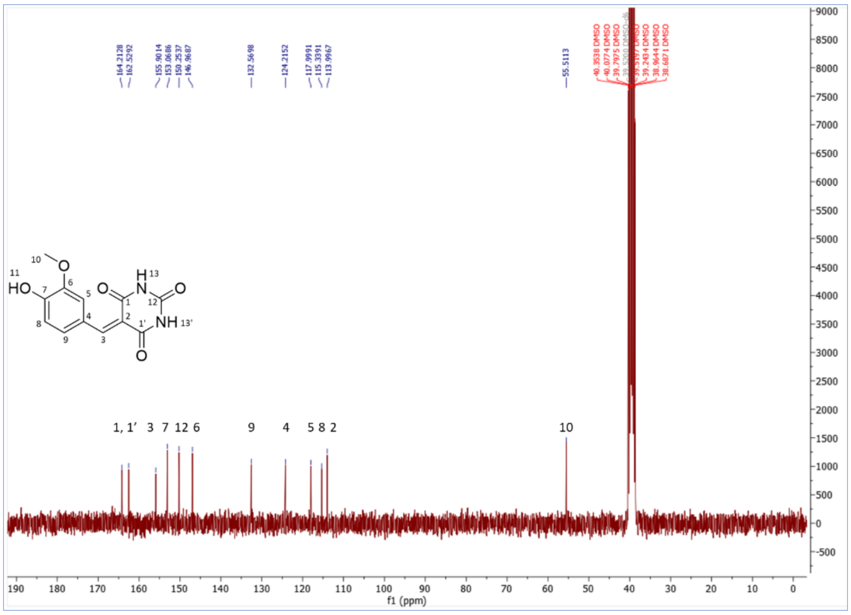

Fig. S5: ^{13}C NMR (75 MHz, 25 °C, DMSO-d_6δ: 164.2 (C-1), 162.5 (C-1'), 155.9 (C-3), 153.1 (C-7), 150.3 (C-12), 147.0 (C-6), 132.6 (C-9), 124.2 (C-4), 118.0 (C-5), 115.3 (C-8), 114.0 (C-2), 55.5 (C-10).

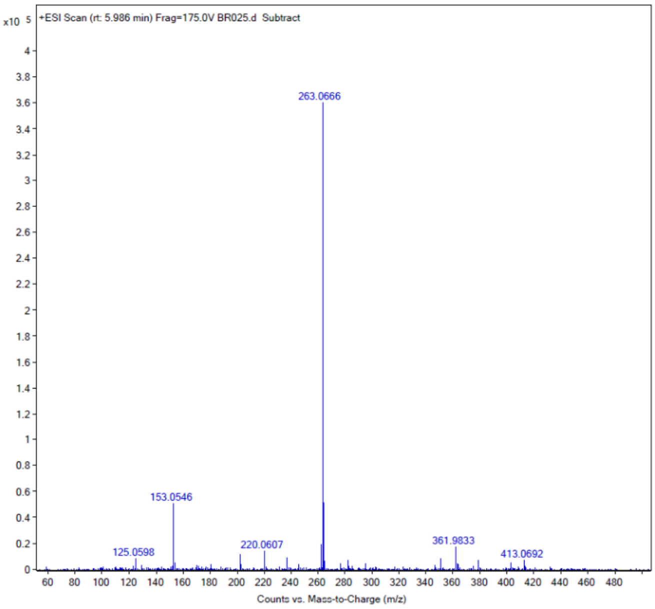

Fig. S6: TOF MS ES+: $[M+H]^+$ for $C_{12}H_{11}N_2O_5$: *m/z* 263.0668; found: *m/z* 263.0666.

Caffeyl barbituric acid (CafBA): filtration led to a yellow orange powder (92%); m.p. 151 °C; UV: λ_{max} (EtOH, nm) 409, ε ($L.mol^{-1}.cm^{-1}$) 21700

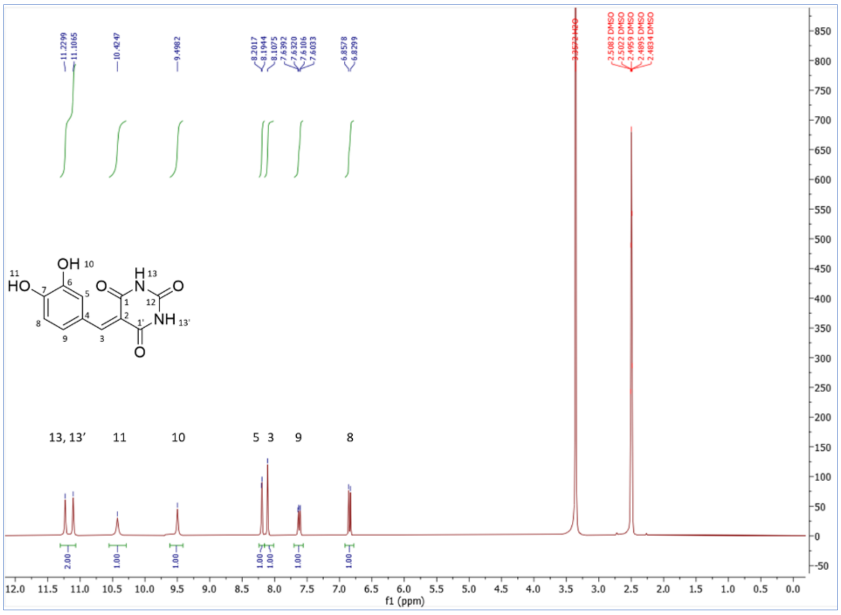

Fig. S7: ^{1}H NMR (300 MHz, 25 °C, DMSO-d_6) δ: 11.30 and 11.11 (2H, 2s, H-13 and H-13'), 10.42 and 9.50 (2H, 2s, H-11 and H-10), 8.20 (1H, d, *J* = 2.2 Hz, H-5), 8.11 (1H, s, H-3), 7.62 (1H, dd, *J* = 2.2 Hz and *J* = 8.6 Hz, H-9), 6.84 (1H, d, *J* = 8.4 Hz, H-8).

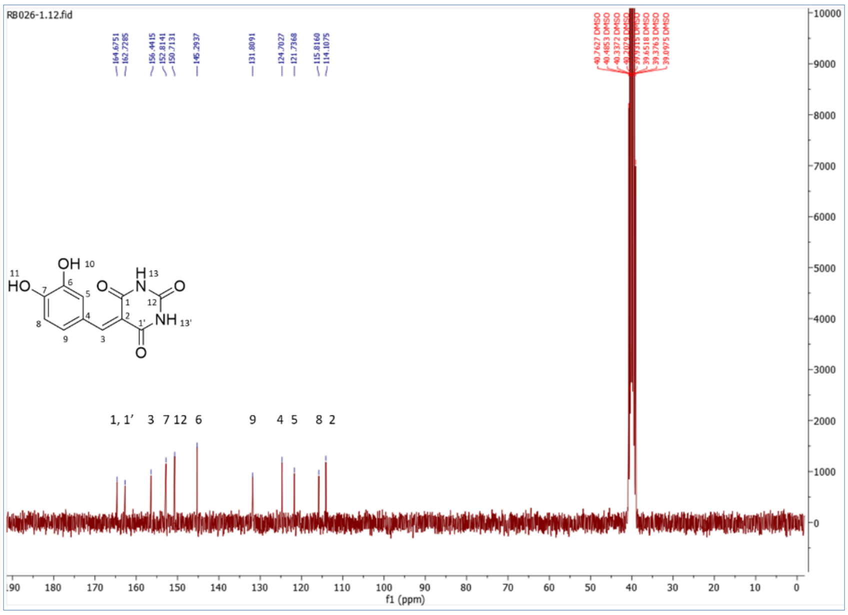

Fig. S8: ^{13}C NMR (75 MHz, 25 °C, DMSO-d_6) δ: 164.7 (C-1), 162.7 (C-1'), 156.4 (C-3), 152.8 (C-7), 150.7 (C-12), 145.3 (C-6), 131.8 (C-9), 124.7 (C-4), 121.7 (C-5), 115.8 (C-8), 114.1 (C-2).

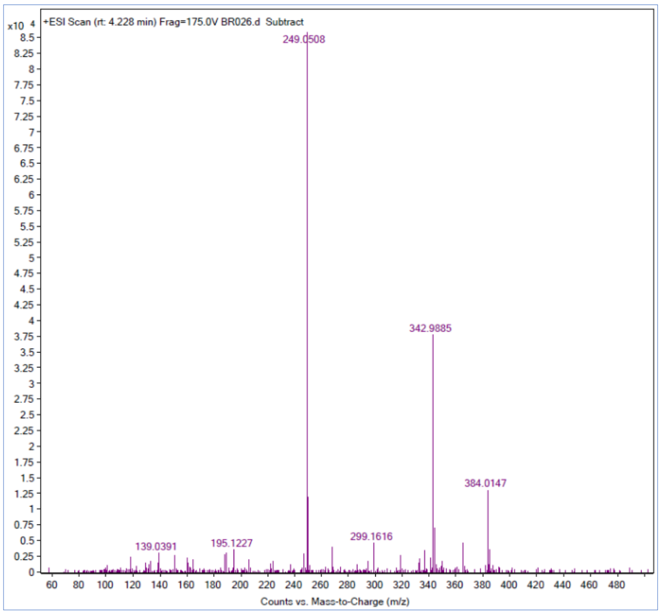

Fig. S9: TOF MS ES+: $[M+H]^+$ for $C_{11}H_9N_2O_5$: *m/z* 249.0511; found: *m/z* 249.0508.

Sinapyl barbituric acid (SBA): filtration led to an orange powder (96%); m.p. 149 °C; UV: λ_{max} (EtOH, nm) 416 and 500, ε ($L.mol^{-1}.cm^{-1}$) 23781 and 29500.

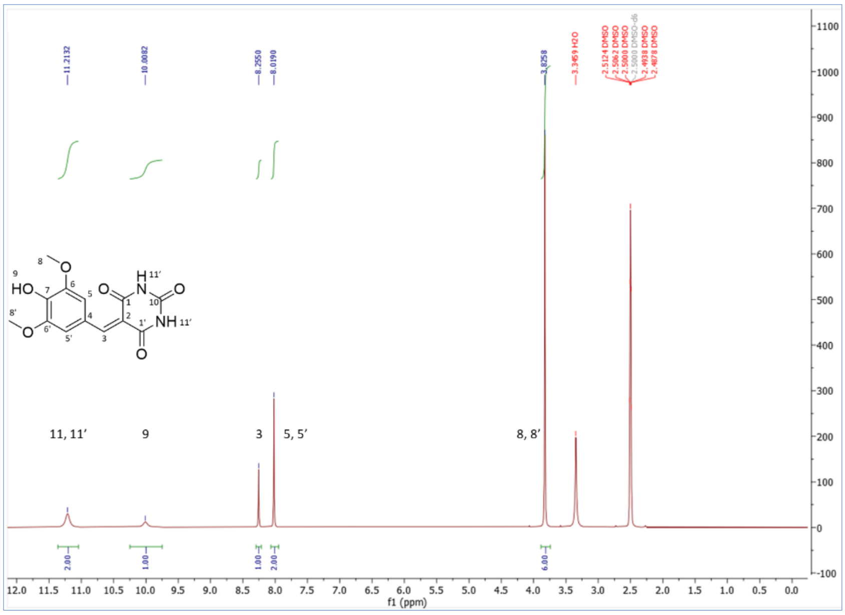

Fig. S10: ^1H NMR (300 MHz, 25 °C, DMSO-d_6) δ: 11.21 (2H, s, H-11 and H-11'), 10.01 (1H, 1s, H-9), 8.26 (1H, s, H-3), 8.02 (2H, s, H-5 and H-5'), 3.82 (6H, s, H-8 and H-8').

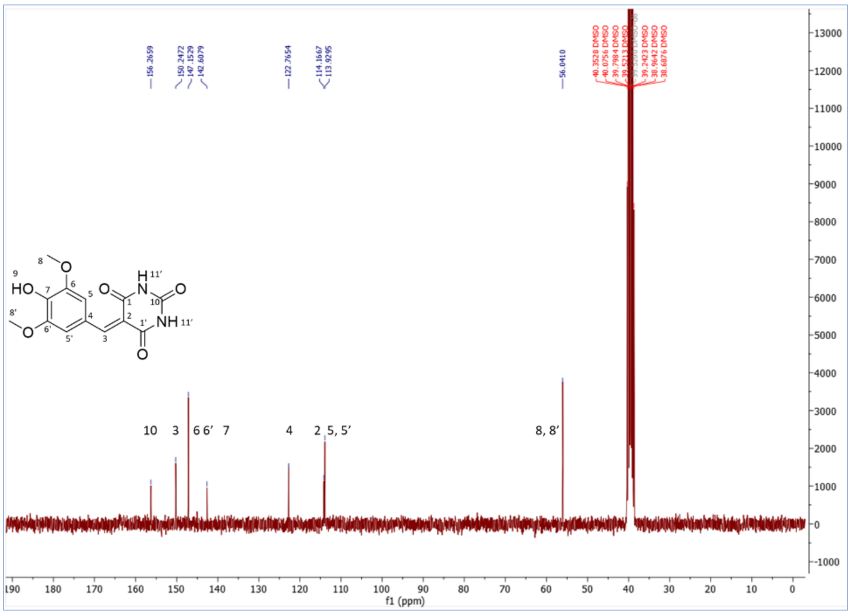

Fig. S11: ^{13}C NMR (75 MHz, 25 °C, DMSO-d_6) δ: 165.2 (C-1), 162.3 (C-1'), 156.3 (C-3), 150.2 (C-10), 147.2 (C-6 and C-6'), 142.6 (C-7), 122.8 (C-4), 114.2 (C-2), 113.9 (C-5 and C-5'), 56.0 (C-8 and C-8').

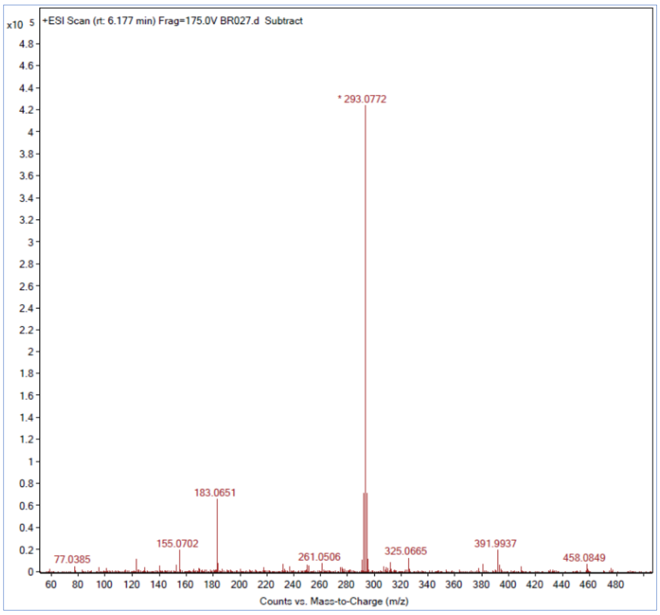

Fig. S12: TOF MS ES+: $[M+H]^+$ for $C_{13}H_{13}N_2O_6$: *m/z* 293.0774; found: *m/z* 293.0772.

4-methoxycoumaryl barbituric acid (MeCBA): filtration led to a pale-yellow powder (99%); m.p. 265 – 267 °C; UV: λ_{max} (EtOH, nm) 374, ε (L.mol^{-1}.cm^{-1}) 16500.

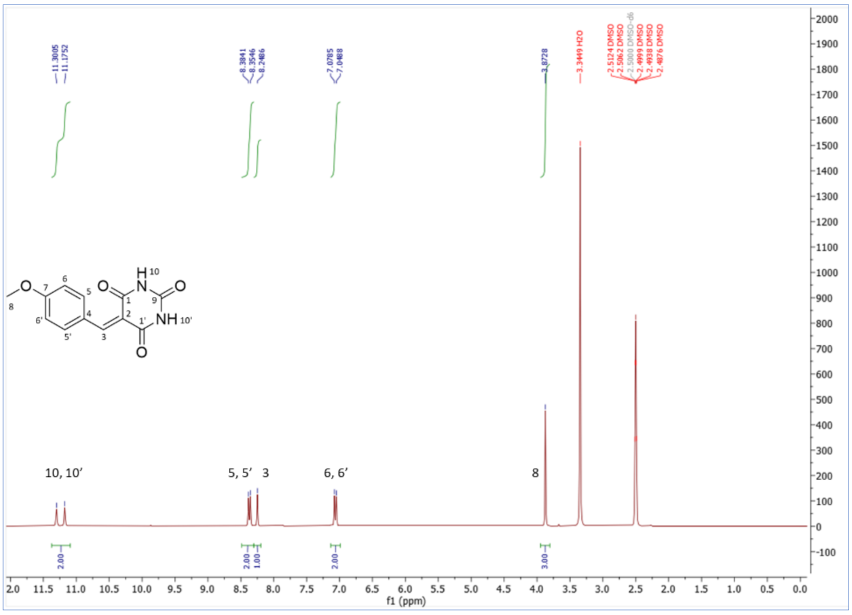

Fig. S13: ^{1}H NMR (300 MHz, 25 °C, DMSO-d$_6$) δ: 11.30 and 11.18 (2H, 2s, H-10 and H-10'), 8.37 (2H, d, *J* = 8.8 Hz, H-5 and H-5'), 8.25 (1H, s, H-3), 7.06 (2H, d, *J* = 8.8 Hz, H-6 and H-6'), 3.87 (3H, s, H-8).

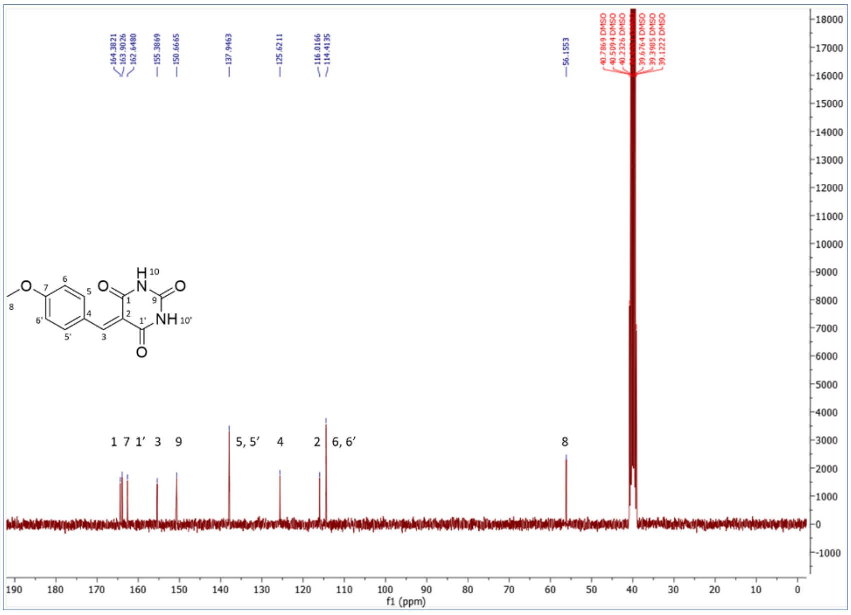

Fig. S14: ^{13}C NMR (75 MHz, 25 °C, DMSO-d$_6$) δ: 164.0 (C-1), 163.5 (C-7), 162.2 (C-1'), 155.0 (C-3), 150.2 (C-9), 137.5 (C-5 and C-5'), 125.2 (C-4), 115.6 (C-2), 114.0 (C-6 and C-6'), 55.7 (C-8).

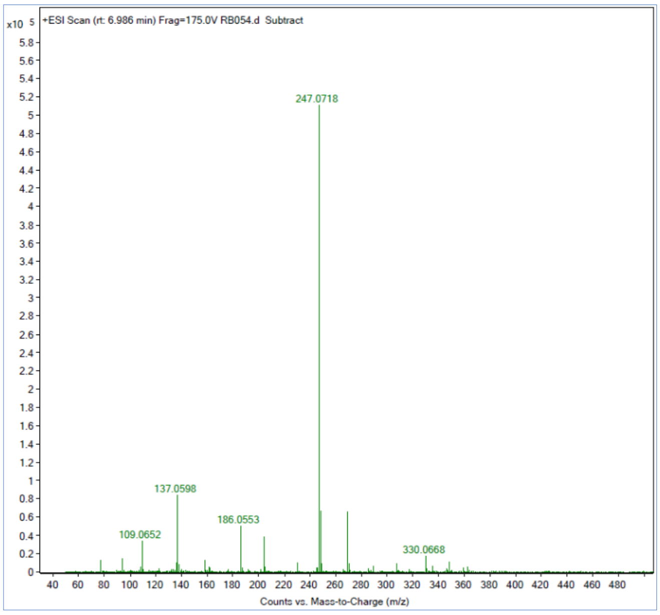

Fig. S15: TOF MS ES+: $[M+H]^+$ for $C_{12}H_{11}N_2O_4$: *m/z* 247.0719; found: *m/z* 247.0718.

ii. Dimethyl barbituric acid series:

Coumaryl dimethyl barbituric acid (CDBA): filtration led to a pale-yellow powder (94%); m.p. not-determined, 5% thermo-degradation: $T_{d5\%}$ = 245 °C; UV: λ_{max} (EtOH, nm) 387, ε ($L.mol^{-1}.cm^{-1}$) 21100.

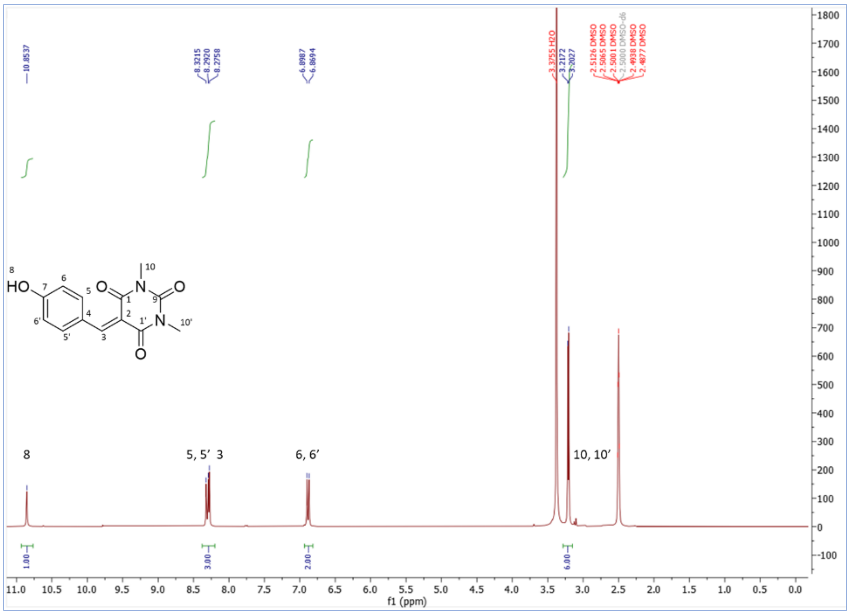

Fig. S16: ^{1}H NMR (300 MHz, 25 °C, DMSO-d_6) δ: 10.85 (1H, s, H-8), 8.31 (2H, d, *J* = 8.8 Hz, H-5 and H-5'), 8.28 (1H, s, H-3), 6.88 (2H, d, *J* = 8.8 Hz, H-6 and H-6'), 3.22 and 3.20 (6H, 2s, H-10 and H-10').

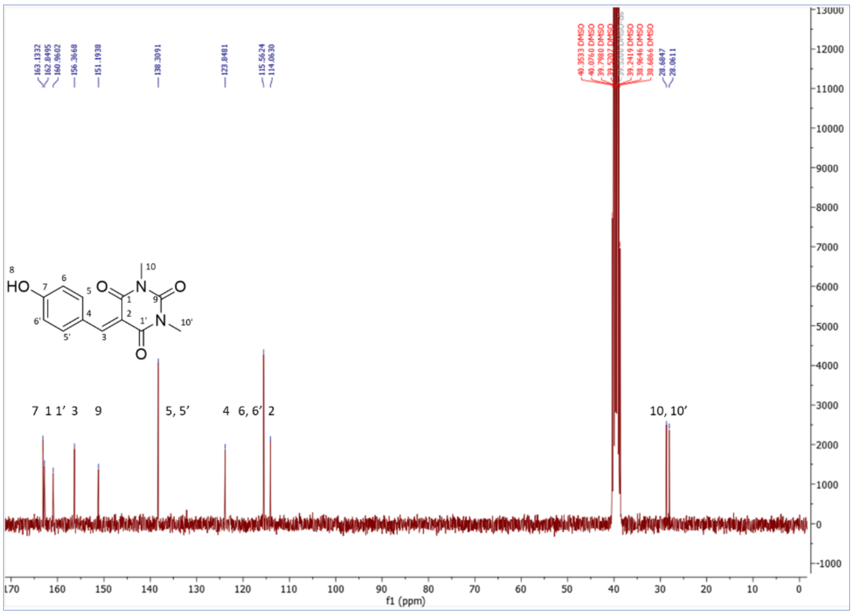

Fig. S17: ^{13}C NMR (75 MHz, 25 °C, DMSO-d_6) δ: 163.1 (C-7), 162.8 and 161.0 (C-1 and C-1'), 156.4 (C-3), 151.2 (C-9), 138.3 (C-5 and C-5'), 123.8 (C-4), 115.6 (C-6 and C-6'), 114.1 (C-2), 28.7 and 28.1 (C-10 and C-10').

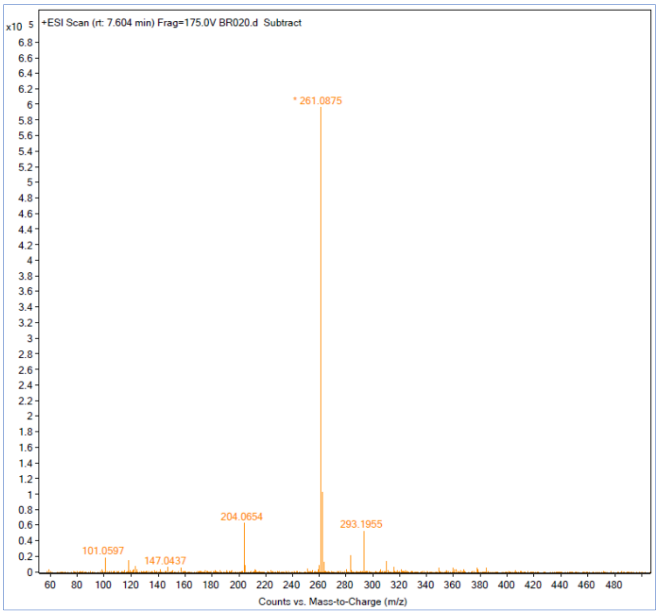

Fig. S18: TOF MS ES+: $[M+H]^+$ for $C_{13}H_{13}N_2O_4$: *m/z* 261.0875; found: *m/z* 261.0875.

Ferulyl dimethyl barbituric acid (FDBA): filtration led to a yellow powder (99%); m.p. 227 – 229 °C; UV: λ_{max} (EtOH, nm) 406, ε ($L.mol^{-1}.cm^{-1}$) 29700.

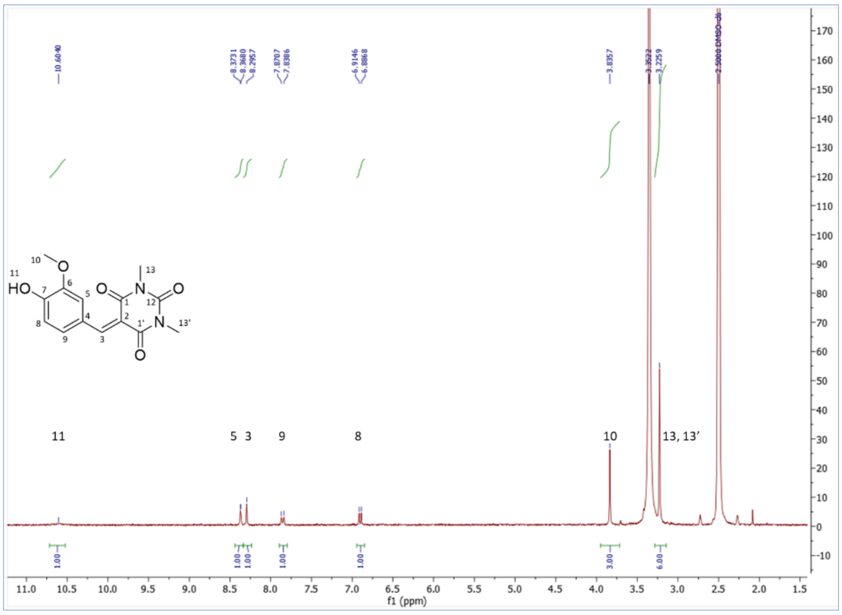

Fig. S19: ^1H NMR (300 MHz, 25 °C, DMSO-d_6) δ: 10.60 (1H, s, H-11), 8.37 (1H, d, *J* = 1.5 Hz, H-5), 8.29 (1H, s, H-3), 7.85 (1H, dd, *J* = 1.5 Hz and *J* = 8.3 Hz, H-9), 6.89 (1H, d, *J* = 8.3 Hz, H-8), 3.83 (3H, s, H-10), 3.22 (6H, s, H-13 and H-13').

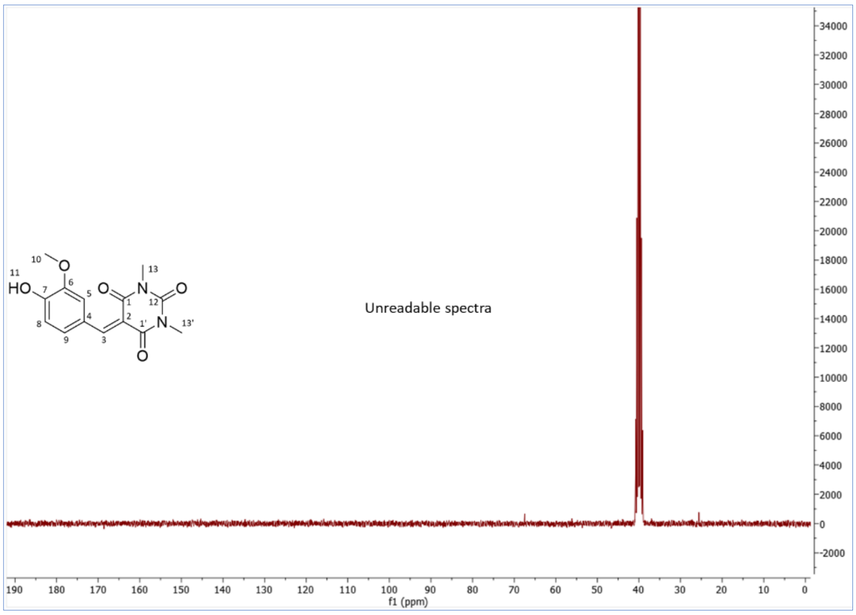

Fig. S20: ^{13}C NMR analysis not possible due to poor solubility resulting into low concentration: unreadable spectra.

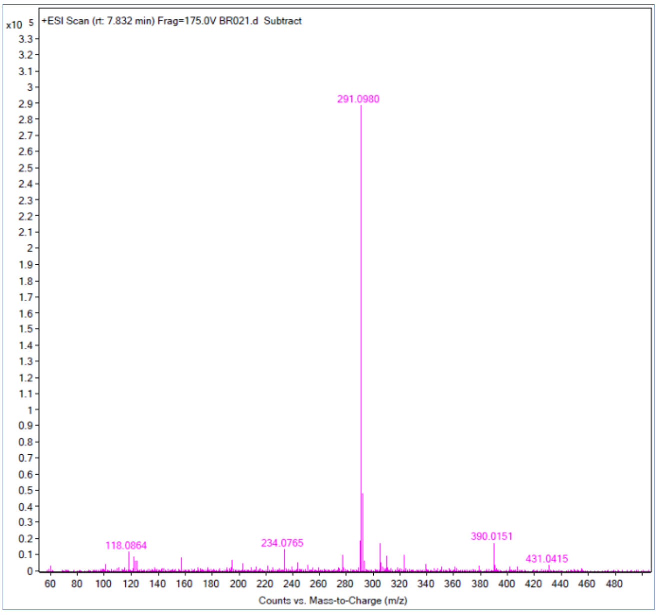

Fig. S21: TOF MS ES+: $[M+H]^+$ for $C_{14}H_{15}N_2O_5$: *m/z* 291.0981; found: *m/z* 291.0980.

Caffeyl dimethyl barbituric acid (CafDBA): filtration led to a yellow powder (88%); m.p. not-determined, 5% thermo-degradation: $T_{d5\%}$ = 257 °C; UV: λ_{max} (EtOH, nm) 411, ε (L.mol^{-1}.cm^{-1}) 21100.

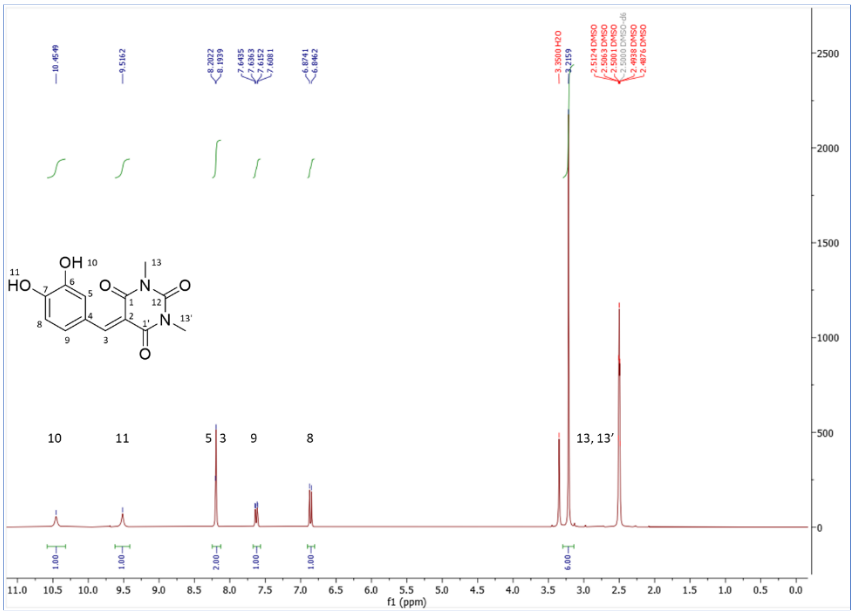

Fig. S22: ^{1}H NMR (300 MHz, 25 °C, DMSO) δ: 10.44 and 9.51 (2H, 2s, H-10 and H-11), 8.19 (2H, s and d, H-3 and H-5), 7.62 (1H, dd, *J* = 1.9 Hz and *J* = 8.6 Hz, H-9), 6.85 (1H, d, *J* = 8.4 Hz, H-8).

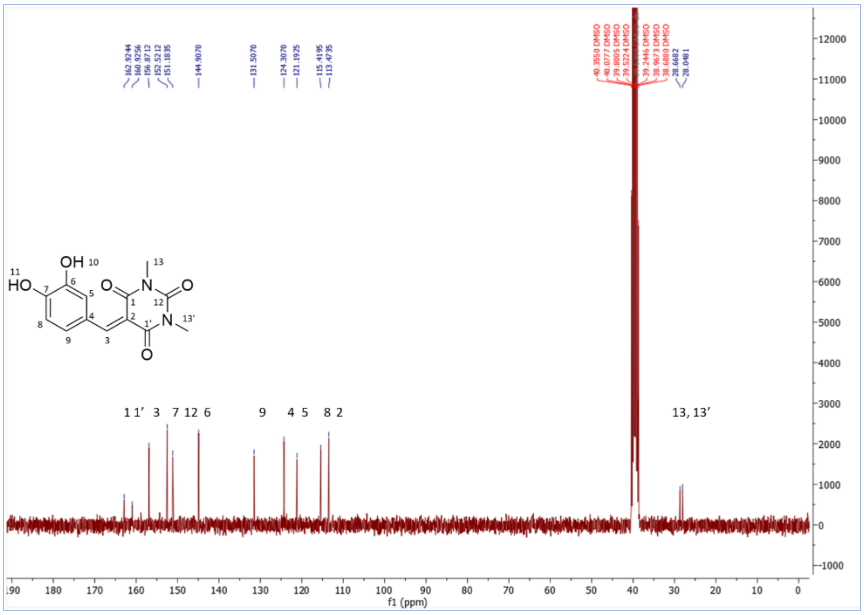

Fig. S23: ^{13}C NMR (75 MHz, 25 °C, DMSO) δ: 162.9 and 160.9 (C-1 and C-1'), 156.9 (C-3), 152.5 (C-7), 151.2 (C-12), 144.9 (C-6), 131.5 (C-9), 124.3 (C-4), 121.2 (C-5), 115.4 (C-8), 113.5 (C-2), 28.7 and 28.1 (C-13 and C-13').

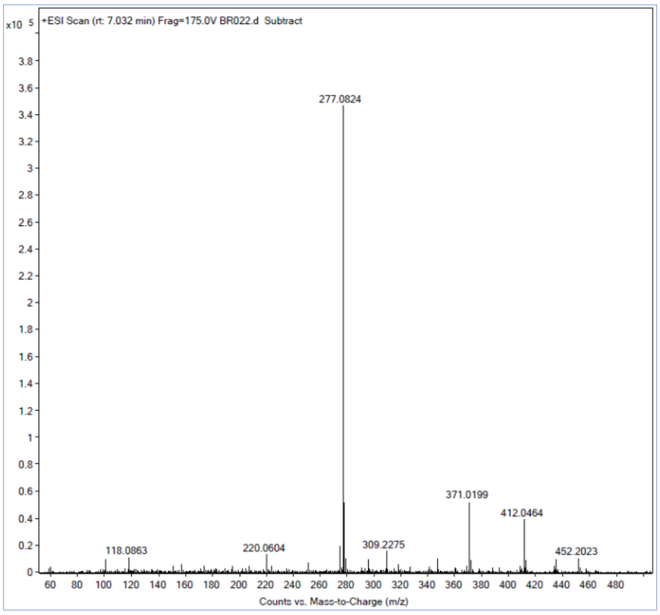

Fig. S24: TOF MS ES+: $[M + H]^+$ for $C_{13}H_{13}N_2O_5$: *m/z* 277.0824; found: *m/z* 277.0824.

Sinapyl dimethyl barbituric acid (SDBA): filtration led to a yellow orange powder (100%); m.p. 250 – 252 °C; UV: λ_{max} (EtOH, nm) 419 and 502, ε (L.mol^{-1}.cm^{-1}) 22993 and 21100.

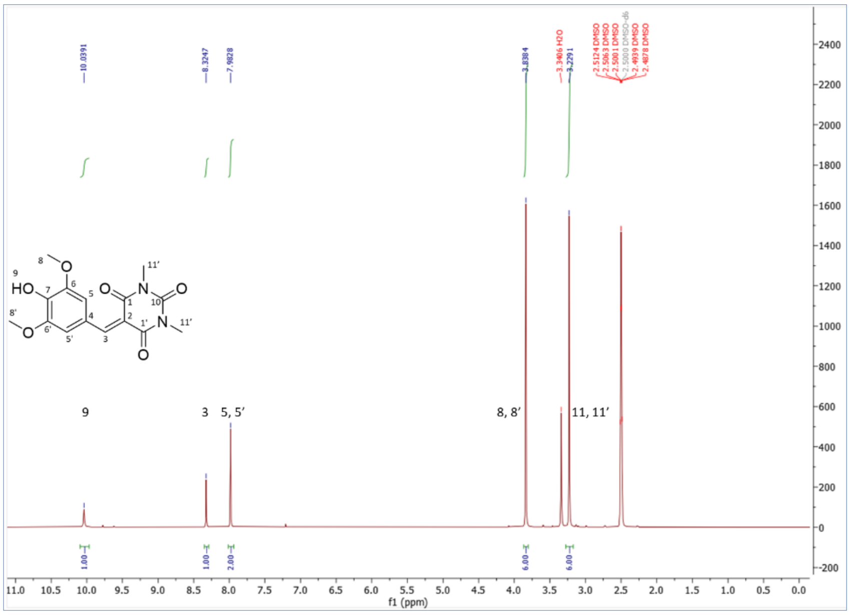

Fig. S25: ^{1}H NMR (300 MHz, 25 °C, DMSO-d$_6$) δ: 10.03 (1H, 1s, H-9), 8.32 (1H, s, H-3), 7.98 (2H, s, H-5 and H-5'), 3.83 (6H, s, H-8 and H-8'), 3.22 (6H, s, H-11 and H-11').

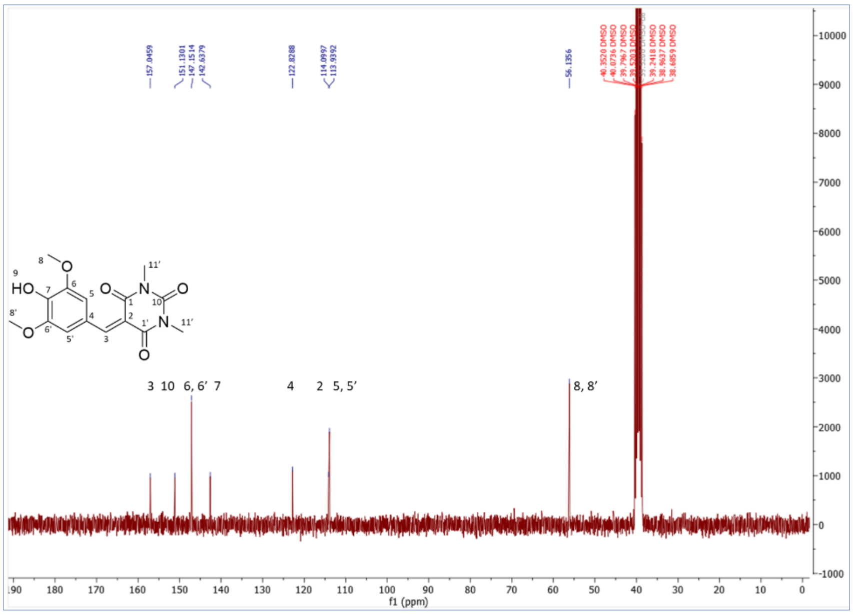

Fig. S26: ^{13}C NMR (75 MHz, 25 °C, DMSO-d_6) δ: 161.9 (C-1 and C-1'), 157.0 (C-3), 151.1 (C-10), 147.2 (C-6 and C-6'), 142.6 (C-7), 122.8 (C-4), 114.1 (C-2), 113.9 (C-5 and C-5'), 56.1 (C-8 and C-8'), 28.1 (C-11 and C-11').

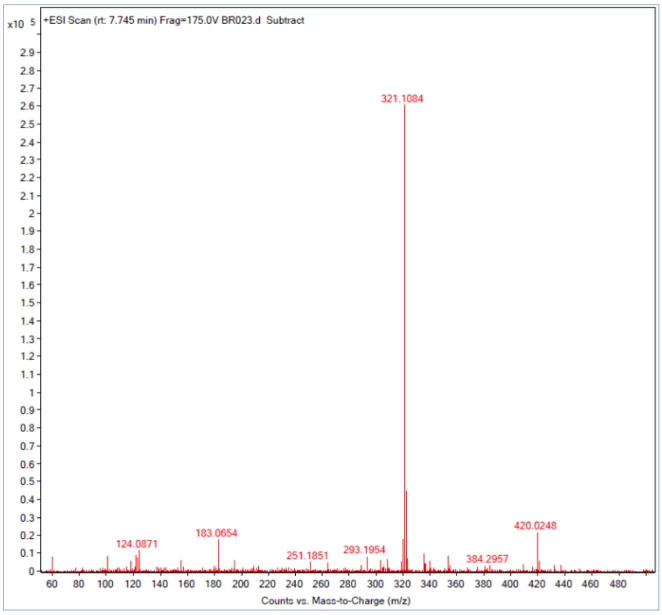

Fig. S27: TOF MS ES+: $[M+H]^+$ for $C_{15}H_{17}N_2O_6$: *m/z* 321.1087; found: *m/z* 321.1084.

4-methoxycoumaryl dimethyl barbituric acid (MeCDBA): filtration led to a pale-yellow powder (99%); m.p. 148 – 150 °C; UV: λ_{max} (EtOH, nm) 375, ε (L.mol^{-1}.cm^{-1}) 23600.

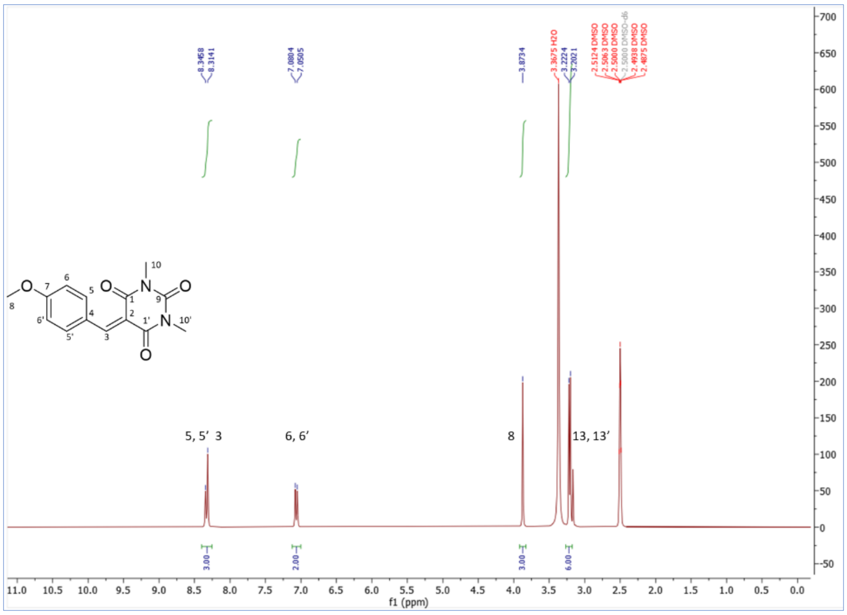

Fig. S28: ^{1}H NMR (300 MHz, 25 °C, DMSO-d$_6$) δ: 8.33 (2H, d, *J* = 9.5 Hz, H-5 and H-5'), 8.31 (1H, s, H-3), 7.07 (2H, d, *J* = 9.0 Hz, H-6 and H-6'), 3.87 (3H, s, H-8), 3.22 and 3.20 (6H, 2s, H-10 and H-10').

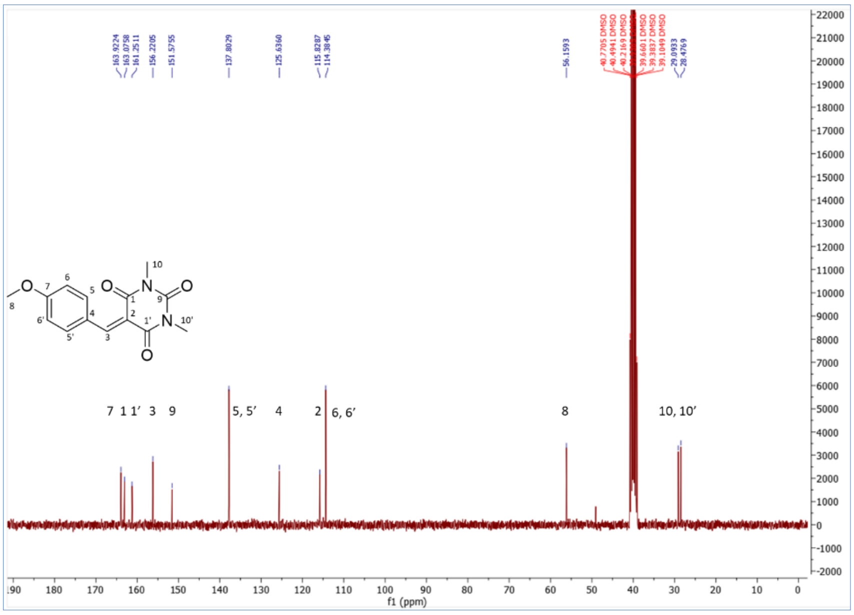

Fig. S29: ^{13}C NMR (75 MHz, 25 °C, DMSO-d$_6$) δ: 163.5 (C-7), 162.5 and 160.8 (C-1 and C-1'), 155.8 (C-3), 151.3 (C-9), 137.4 (C-5 and C-5'), 125.2 (C-4), 115.4 (C-2), 114.0 (C-6 and C-6'), 55.7 (C-8), 28.7 and 28.1 (C-10 and C-10').

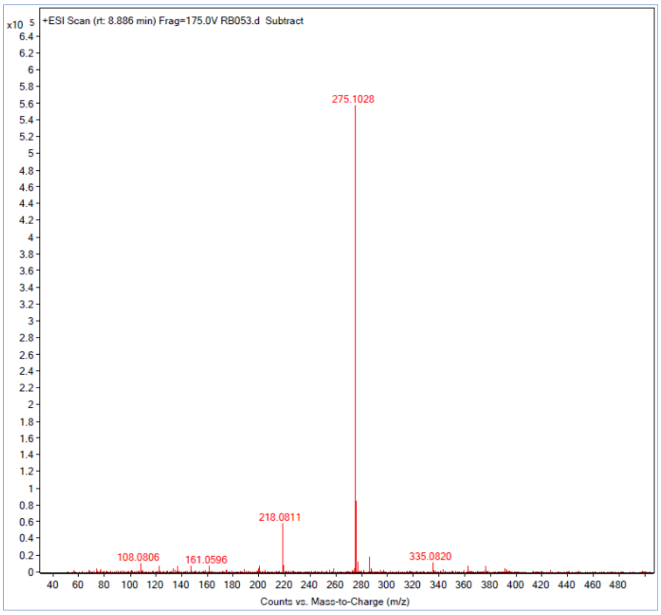

Fig. S30: TOF MS ES+: $[M+H]^+$ for $C_{14}H_{15}N_2O_4$: *m/z* 275.1032; found: *m/z* 275.1028.

C. Additional steady-state measurements.

The second absorption peak observed in the UV-visible spectra of the barbiturics in DMSO reported in Fig. 1 within the paper is attributed to the deprotonated species (anion) formed in solution as a result of proton transfer from the phenolic hydrogen to the solvent, since DMSO can act as a proton acceptor.[1,2] Further experiments were conducted to validate this anion formation hypothesis. First, we prepared a solution of 10 µM FDBA in DMSO and recorded the UV-vis spectrum revealing both features at 405 nm and 485 nm as shown in Fig. S31(a). Only the red-shifted absorption feature at 485 nm was observed with the addition of an organic base (triethylamine) to the solution, whereas subsequent addition of acetic acid to the solution led to a blue-shifted spectrum exhibiting only the 405 nm feature. This confirms our assignment of the 485 nm absorption peak to the formation of an anionic species in DMSO solution due to a proton transfer to the solvent. The absorption spectra in DMSO are also dependent on the barbituric concentration. At a very low concentration (5 µM), only the 485 nm peak attributed to the anion is present, while both peaks are present at higher concentrations. Such spectral behaviour in DMSO has been reported in the literature.[1] The absence of an analogous 485 nm feature in the UV-vis spectrum of MeCDBA shown in Fig. S31(b), where the hydroxy group is substituted with methoxy group, lends further support to our assignment of this feature to the deprotonated parent.

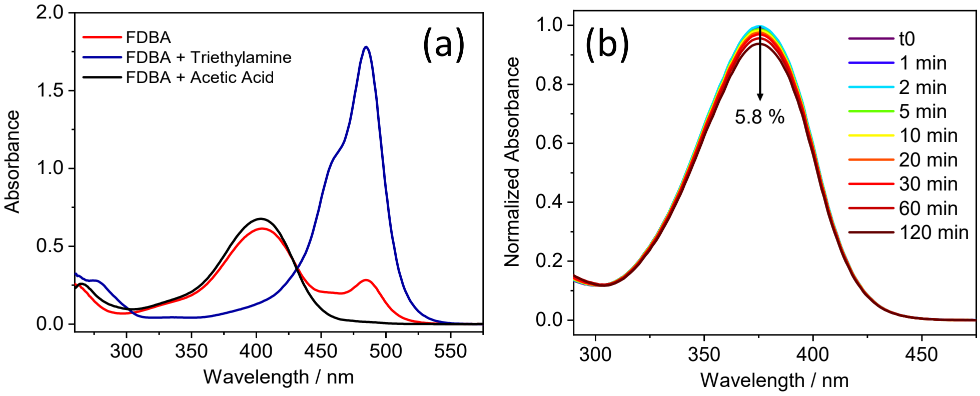

Fig. S31: UV-visible spectra of (a) 10 µM FDBA at different pH, (b) MeCDBA at varying time delay during irradiation with arc lamp.

The summary of the long-term photostability for all the barbituric derivatives obtained following irradiation at the λ_{max} of each sample with a Fluorolog are reported in Table S2. The results show that, following irradiation for 2 hrs in both solvents, all the barbiturics are photostable with only a mild reduction (*i.e.* < 6 %) in absorbance at λ_{max} in both solvents.

In order to investigate the possible formation of any long-lived photoproducts upon UV excitation of the barbiturics studied herein, ^{1}H NMR spectra were recorded in deuterated DMSO before and after two hours of continuous irradiation under a solar simulator with irradiance equivalent to 1 sun (1000 W/m^2). As shown in Fig. S32, the data did not reveal any new peaks after sample irradiation, suggesting little or no photoproduct formation.

Table S2. Summary of the photostability analysis of the barbituric derivatives in DMSO and dioxane

Molecule	DMSO		DIOXANE	
	Peak Absorption (nm)	Absorbance loss (%)	Peak Absorption (nm)	Absorbance loss (%)
CBA	384	1.2	369	0.2
FBA	404	0.9	391	0.2
CafBA	406	0.6	391	0.2
SBA	414	0.7	406	0.3
MeCBA	375	5.1	369	0.3
CDBA	385	1.8	369	0.2
FDBA	405	1.7	391	0.2
CafDBA	410	2.3	391	0.2
SDBA	417	1.0	407	0.3
MeCDBA	375	5.8	369	0.3

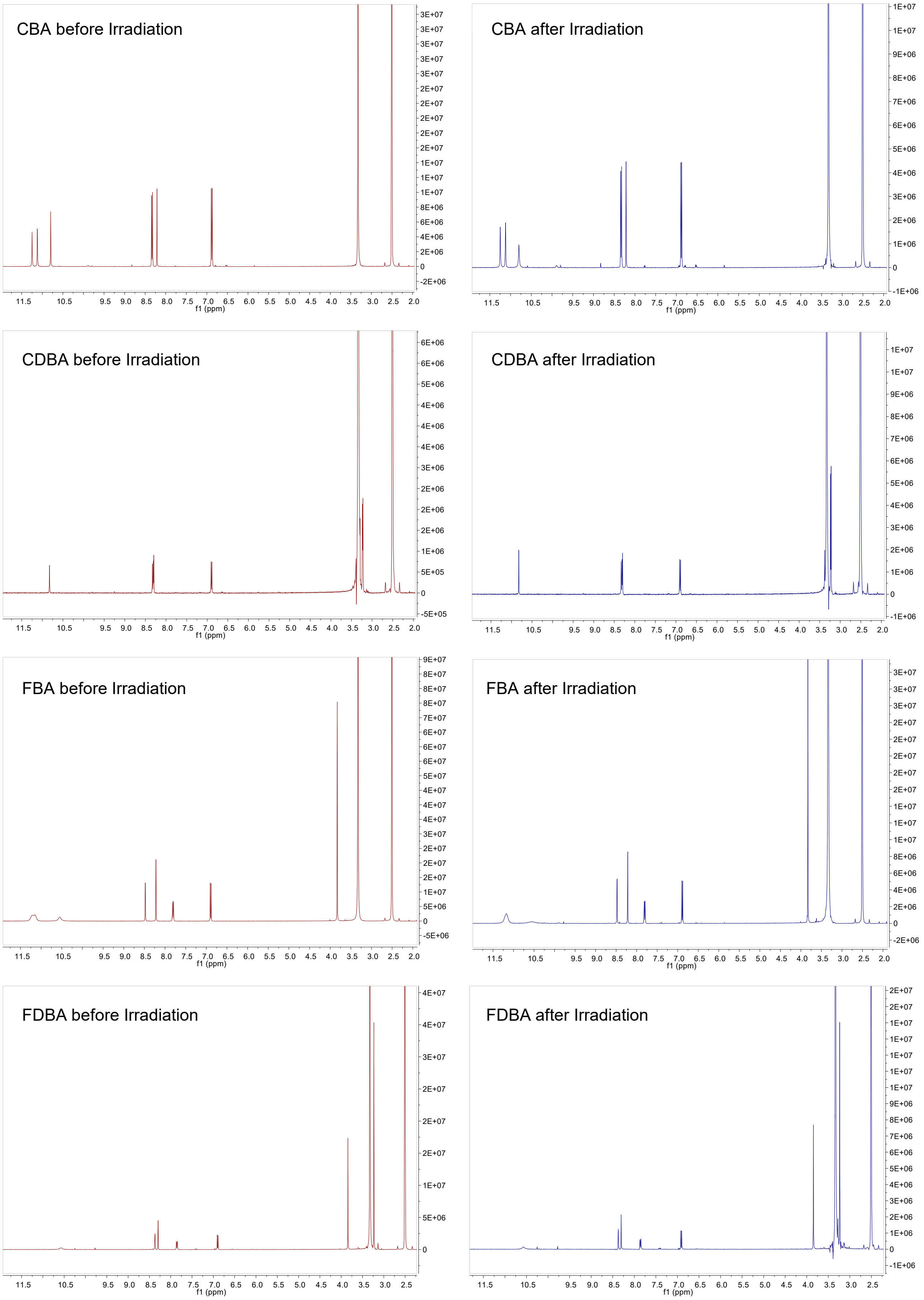

Fig. S32: ^{1}H NMR spectra of before (dark red line) and after irradiation (dark blue line) for CBA, CDBA, FBA and FDBA in DMSO solution. For complete assignment of the NMR peaks refer to ESI section B.

D. Additional TEAS measurements

In addition to the transient electronic absorption spectroscopy (TEAS) measurements of CDBA, MeCDBA, and FDBA in DMSO reported and discussed in the paper, TEAS measurements were also carried out for CBA and FBA in DMSO, and the acquired data are reported in Fig. S33. These data have the same features and dynamics as those reported for CDBA and FDBA in the paper, indicating that the substitution of the hydrogen atom bonded to the nitrogen in the barbituric unit with a methyl group does not significantly alter the photodynamics of this class of molecules. We now discuss the evolution associated difference spectra (EADS) obtained from the sequential fittings for data presented in Fig. 2 within the manuscript and in the Fig. S33. The EADS of CBA, CDBA, and MeCDBA (coumaryl series) are similar, while those of FBA and FDBA (ferulyl) are also mutually comparable. Hence, where appropriate, we discuss the EADS of the coumaryl and ferulyl derivatives separately. In both series, EADS1 shows ESA and SE indicating that, on this time scale, the system is in the excited state. For the coumaryl series, EADS2 shows GSB, the absence of SE and an ESA in the SE region that sensibly corresponds with a hot ground state, indicating that the system is back in the ground state. For the ferulyl series, in contrast, EADS2 shows ESA, SE, and GSB features, indicating that both excited-state and ground-state species are present. This accords with the discussion within the manuscript on the role of the $S_{1\text{-}LE}$ state. In both series, EADS3 shows a GSB and a blue-shifted ESA relative to the ESA in EADS2; this is a signature of vibrational cooling in the ground state. Finally, EADS4 in all systems reveals a minor incomplete recovery of the GSB, the origin of which was

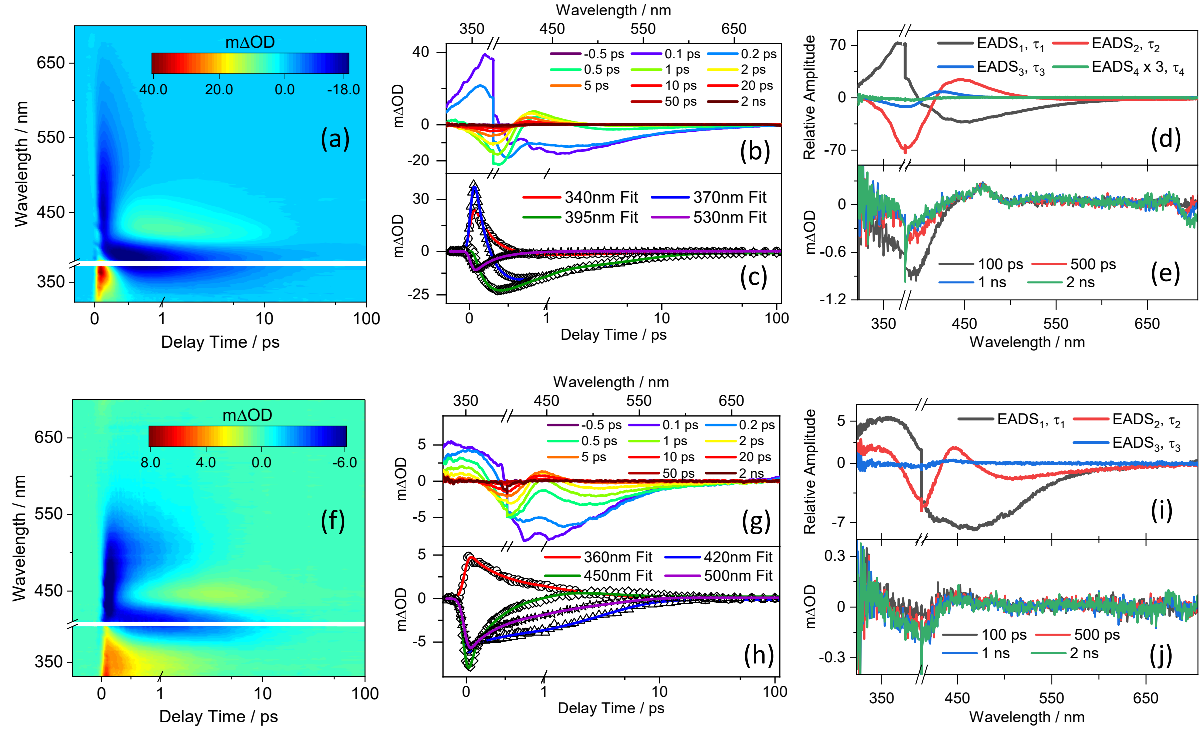

Fig. S33: TEA spectra, obtained in DMSO for 1 mM solutions of (a) CBA photoexcited at 385 nm, and (f) FBA photoexcited at 404 nm, shown as false colour maps. In all cases, the delay times are presented as a linear plot until 1 ps and then on a logarithmic scale between 1 and 100 ps. The same data are represented as line plots of mΔOD *vs* probe wavelength at selected pump-probe time delays in panels (b) and (g) for CBA and FBA, respectively. Panels (c) and (h) show transients (raw data as symbol and fits as solid lines) at selected probe wavelengths for CBA and FBA, respectively. The EADS produced by the fitting procedure are shown in panels (d) CBA and (i) FBA. Where applicable, $EADS_4$ is multiplied by three as a visual aid. The high average, longer delay time, TEA spectra are shown in panels (e) and (j) for CBA and FBA, respectively.

discussed within the manuscript. We note the absence of EADS4 in the fit of FBA spectra obtained in DMSO; this is attributed to the poor signal-to-noise ratio at longer time delays. However, the obtained high average (100 scans) 2 ns TEAS measurement for this sample reported in Fig. S33j revealed incomplete GSB recovery, indicating that the long-lived species observed in other barbiturics is also present in FBA.

To further investigate the potential influence of solute-solvent interaction on the photochemical process in these series of molecules following photoexcitation, TEAS measurements were also carried out in dioxane (a weakly interacting solvent) and the results compared with those obtained in DMSO (a strongly interacting polar solvent). The TEA spectra obtained in dioxane are presented in Fig. S34 for CBA, CDBA, and MeCDBA, and in Fig. S35 for FBA and FDBA. The extracted time constants obtained from the global fit of these data are reported in Table 1 in the paper and Table S3. The observed features in the TEA spectra and the extracted time constants are similar to those observed in DMSO solution. Briefly, the negative feature in the TEA spectra of CBA, CDBA, and MeCDBA, centered at ~370 nm and at ~400 nm in FBA and FDBA, corresponds to the GSB, as was the case in DMSO (see the paper). A second negative feature centered at ~450 nm in all the molecules is assigned to stimulated emission (SE). This feature is more visible in the line plot representation of the data in Figs. S34 and S35. An intense positive feature centered at ~360 nm in CBA, CDBA, FBA and FDBA, and at ~370 nm in MeCDBA is attributed to ESA from the S_1 state. Finally, a second ESA centered at ~420 nm is observed in all the systems. As explained in the main paper, this feature is attributable to the formation of a vibrationally hot electronic ground state following internal conversion from the S_1 state. While the features in the TEA spectra of the barbiturics are similar in both DMSO and dioxane, slight differences are observed in the extracted time constants, which are discussed in the paper. Taken together, these data revealed that the dynamics of the barbiturics studied in this work are, at most, only mildly influenced by the solvent environment.

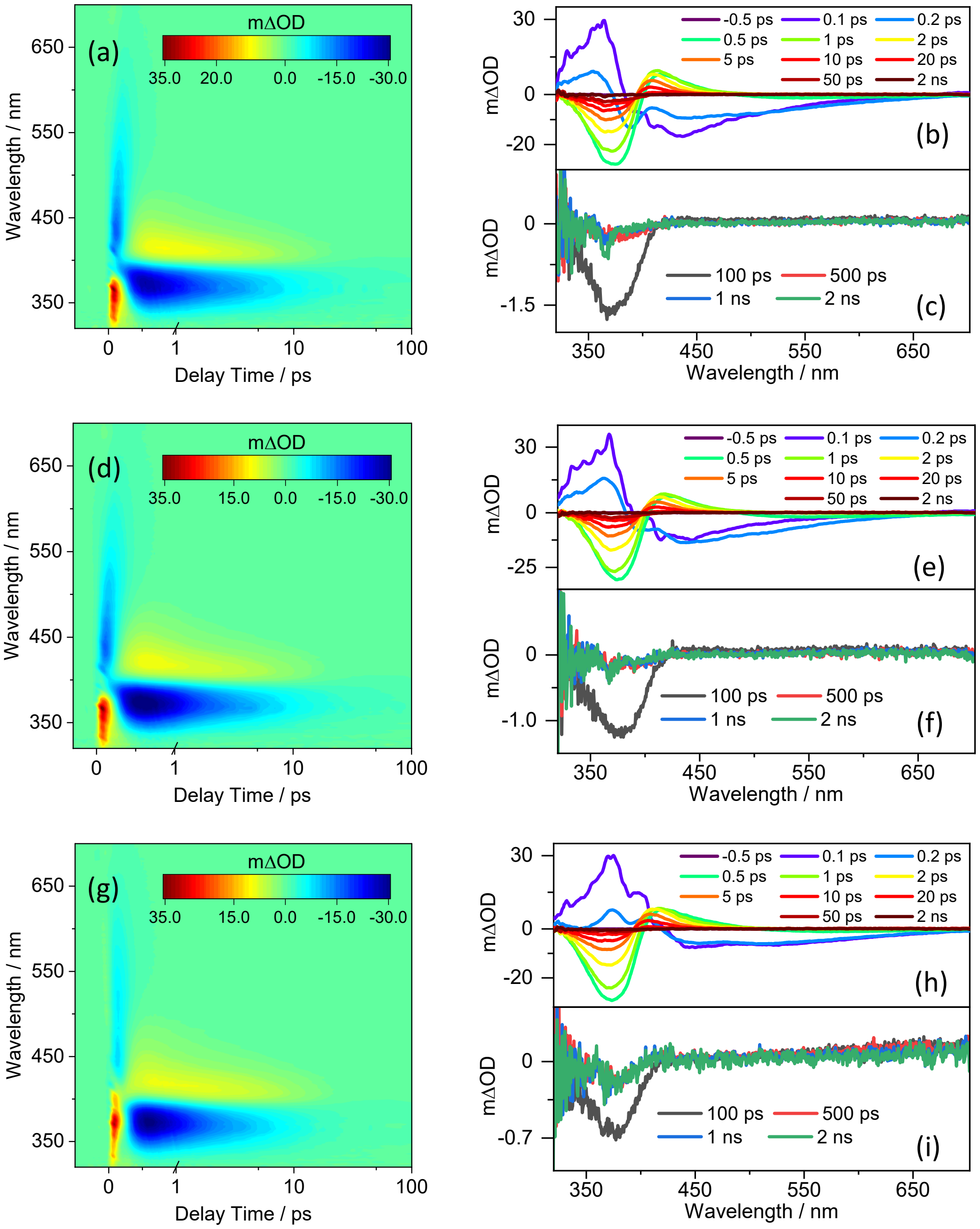

Fig. S34: TEA spectra, obtained in dioxane for 1 mM solutions of (a) CBA, (d) CDBA, and (g) MeCDBA, photoexcited at 369 nm, shown as false colour maps. In all cases, the delay time is presented as a linear plot until 1 ps and then on a logarithmic scale between 1 and 100 ps. The same data are presented as line plots of mΔOD *vs* probe wavelength at selected pump-probe time delays in panels (b), (e), and (h) for CBA, CDBA, and MeCDBA, respectively. The high average, longer delay time, TEA spectra are shown in panels (c), (f), and (i) for CBA, CDBA, and MeCDBA, respectively.

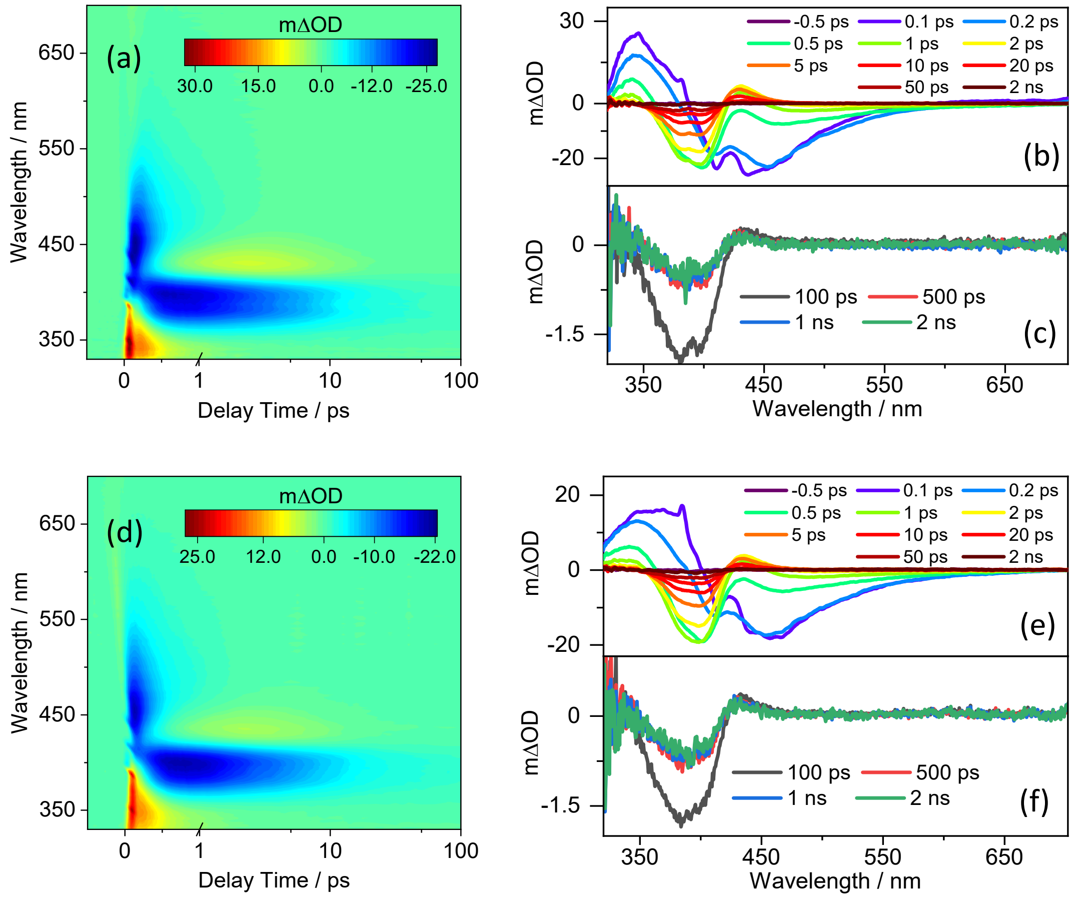

Fig. S35: TEA spectra, obtained in dioxane for 1 mM of (a) FBA and (d) FDBA, photoexcited at 390 nm, shown as false colour maps. In both cases, the delay time is presented as a linear plot until 1 ps and then on a logarithmic scale between 1 and 100 ps. The same data are presented as line plots of mΔOD *vs* probe wavelength at selected pump-probe time delays in panels (b) and (e) for FBA and FDBA, respectively. The high average, longer delay time, TEA spectra are shown in panels (c) and (f) for FBA and FDBA, respectively.

Since TVA spectra (discussed in the paper and Section G herein) were obtained at 30 mM concentration, TEA spectra for CDBA have also been recorded in DMSO at a concentration of 30 mM (but with a reduced optical path length of 6 μm of liquid to minimise white light absorption) to monitor any potential concentration-induced dynamics. The resulting TEA spectra reported in Fig. S36 and the time constant reported in Table S3 revealed that there is little or no concentration effect on the dynamics of the barbiturics between 1 mM and 30 mM.

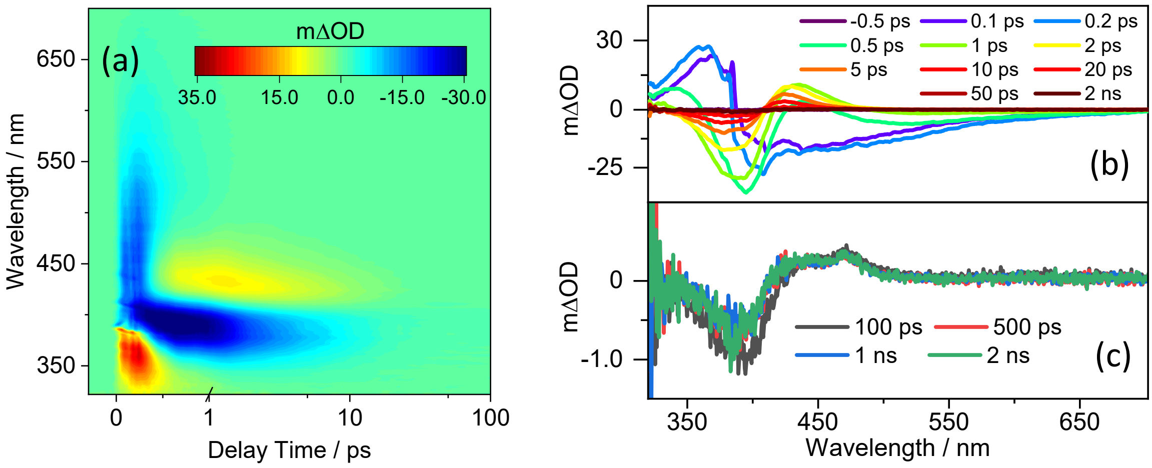

Fig. S36: TEA spectra, obtained for CDBA in DMSO at 30 mM photoexcited at 385 nm, shown as (a) false colour map with the delay time presented as a linear plot until 1 ps and then on a logarithmic scale between 1 and 100 ps and (b) line plot at selected pump-probe time delays. (c) High average, longer time delay, TEA spectra.

E. TEA spectra of FDBA in DMSO photoexcited at 485 nm

In addition to the TEAS measurements obtained in DMSO and dioxane following photoexcitation at the peak absorption (λ_{max}), the dynamics of the FDBA/DMSO red-shifted absorption feature at 485 nm shown in Fig. 1 of the paper has also been investigated to explore the potential influence of the anion species on the overall dynamics when these barbiturics are exposed to broadband UV-visible radiation. As shown in Fig. S37, the TEA spectra revealed similar features as the TEA spectra of FDBA/DMSO photoexcited at 404 nm. Briefly, the spectra show a strong negative feature centred at ~480 nm, which corresponds to the GSB as can be concluded from a comparison with the steady-state UV absorption spectrum. A second negative feature centred at ~600 nm, which further extends to the red-end of the probe window in the TEA spectra, is assigned to SE. Third, an intense positive feature centred at ~400 nm is attributed to ESA. Finally, there is a second ESA centred at ~500 nm. This feature is attributed to the formation of a vibrationally hot electronic ground state molecules following internal conversion. The extracted time constants from the fit of these TEA spectra are given in Table S3. Clearly, the time constants and the EADS extracted from the fit of this spectra are comparable to those of neutral FDBA/DMSO.

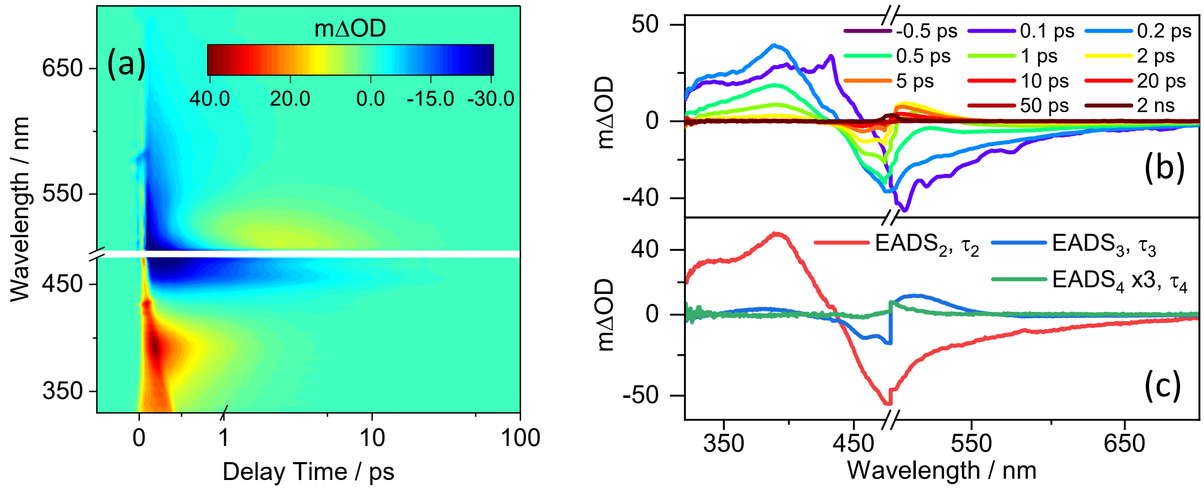

Fig. S37: (a) TEA spectra of 10 μM FDBA in DMSO photoexcited at 485 nm shown as a false colour map plotted as a linear plot until 1 ps and then on a logarithmic scale between 1 and 100 ps. (b) The same data are presented as line plots of mΔOD *vs* probe wavelength at selected pump-probe time delays. (c) EADS produced by the fitting procedure with $EADS_4$ multiplied by three as a visual aid.

While the overall photochemistry picture appears similar, the differences in the dynamics reflected in the associated time-constants warrant discussion. We choose to focus our discussion on the differences between FDBA/DMSO photoexcited at 404 nm and 485 nm (henceforth, referred to as neutral and anion FDBA, respectively). First, we note that τ_1, assigned to the formation of the twisted intramolecular charge transfer (TICT) state for the neutral FDBA, is shorter for the anion FDBA and within the present instrument response. This time-constant will also encompass initial processes such as solvent response, precluding definitive assignment. However, we note that the negative charge on the phenolate oxygen would increase the charge-transfer character during the excitation, and plausibly facilitate a faster population of the TICT state than in neutral FDBA. Second, the returned τ_2 value for neutral FDBA is almost three-times larger than for the anion FDBA. This may be

reconciled by the relatively higher energy barrier that needs to be overcome to access the S_1/S_0 conical intersection in the neutral species. Finally, τ_3 (assigned to vibrational cooling in both cases) is relatively shorter for the anion FDBA than for neutral FDBA. As explained in the paper, this is in line with prior expectations that the interaction of samples with a more strongly interacting (polar) solvent will promote a higher rate of energy transfer.[3] In this case, although the solvent is the same, the anion FDBA presents a stronger solute-solvent interaction compared to the neutral FDBA. Hence, we conclude that the anionic species results in faster dynamics in strongly interacting solvents. Notably, the absence of GSB corresponding to the anion in the neutral molecule TEA spectra (see e.g. Fig. 2) and vice versa implies that only one species (i.e. the neutral or the anion) is excited by the pump-pulse at any selected excitation wavelength, and that the dynamics are independent of one another.

Table S3: Summary of the extracted time-constants and associated errors from the sequential fits of TEA spectra collected for 1 mM solutions of CBA, CDBA, MeCDBA, FBA, and FDBA in DMSO and in dioxane following photoexcitation at the respective maximum absorption wavelengths, and of FDBA in DMSO when photoexcited at 485 nm (corresponding to the deprotonated species). The quoted errors are those produced by the fitting software.

**Solvent**		**CBA**	**CDBA**	**CDBA 30 mM**	**MeCDBA**	**FBA**	**FDBA**	**FDBA ANION**
DMSO	τ_1 /fs	210 ± 40	230 ± 40	220 ± 40	200 ± 40	170 ± 60	180 ± 60	60 ± 40
	τ_2 /fs	390 ± 40	480 ± 40	510 ± 40	440 ± 40	1050 ± 60	1130 ± 60	400 ± 40
	τ_3 /ps	6.40 ± 0.10	6.80 ± 0.20	6.55 ± 0.10	7.68 ± 0.10	6.52 ± 0.12	6.00 ± 0.10	4.63 ± 0.1
	τ_4 /ns	>2	>2	> 2	>2	> 2	> 2	> 2
Dioxane	τ_1 /fs	200± 40	220 ± 40		210± 40	230 ± 40	270 ± 40	--
	τ_2 /fs	330 ± 40	380 ± 40		410 ± 40	710 ± 40	820 ± 40	--
	τ_3 / ps	6.82 ± 0.10	7.10 ± 0.10		8.10 ± 0.04	8.16 ± 0.13	8.43 ± 0.14	--
	τ_4 /ns	> 2	> 2		> 2	> 2	> 2	--

F. Fluorescence emission spectra

Fluorescence emission spectra of CBA, CDBA, MeCDBA, FBA, and FDBA in dioxane at ~3 µM concentration are shown in Fig. S38. The excitation wavelength for the fluorescence measurement is set at the λ_{max} of each molecule. The dips in the emission spectra are artifacts, and arise from subtracting the Raman scattered spectrum of the dioxane solvent from the sample emission spectrum. This effect is exacerbated by the weakness of the emission signal and results in relatively poor signal-to-noise ratio. The stronger fluorescence intensity of FDBA and FBA is in agreement with previous studies and attributed to the relative stabilization of the S_1 state by the *ortho*-methoxy moieties on the benzene ring.[4] The fluorescence quantum yield was estimated at < 1% in all systems studied herein.

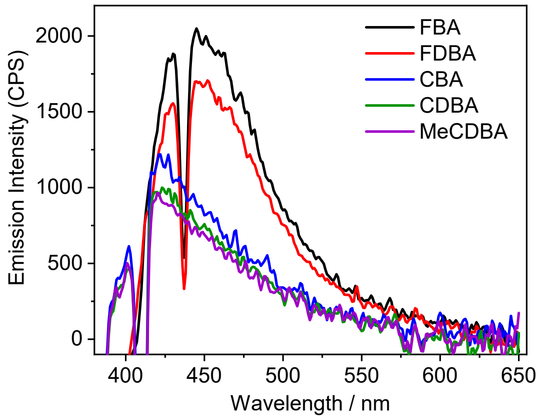

Fig. S38: Fluorescence emission spectra of CBA, CDBA, MeCDBA, FBA, and FDBA.

G. Residuals for the sequential fit to the TEA spectra

The residuals from the sequential global fitting with respect to the raw TEA spectra data (*i.e.,* the difference between the fit and the raw data at each data point) are shown in Fig. S39. The small-signal intensities of the residual compared to the raw TEA spectra demonstrate the quality of the fits.

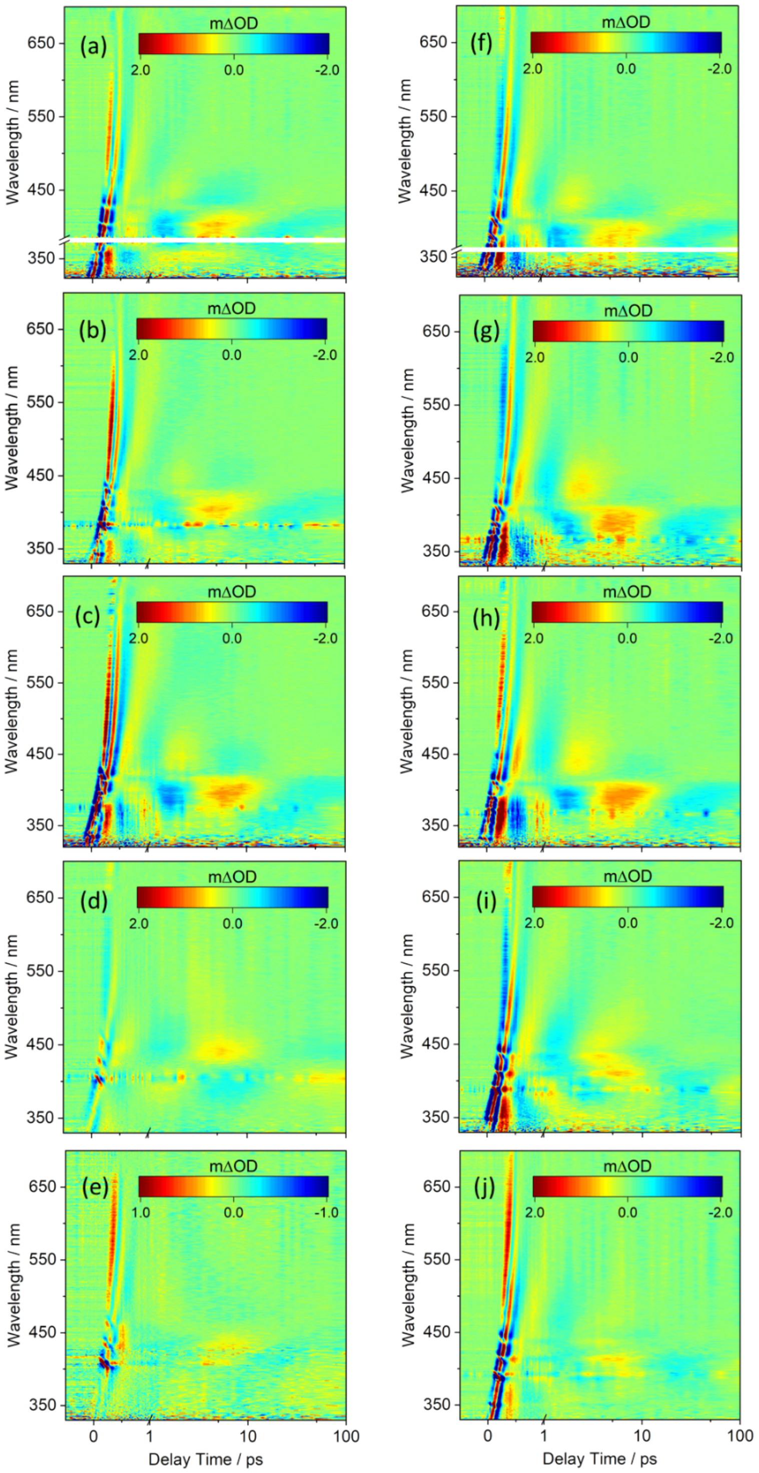

Fig. S39: False colour maps of the residuals of the fit for the phenolic barbiturics. LHS panels (a-e) are the residuals obtained from TEA spectra of CBA, CDBA, MeCDBA, FBA, and FDBA in DMSO, from top to bottom, respectively. RHS panels (f-j) are the analogous residuals obtained from the TEA spectra of the same set of solutes in dioxane.

H. Instrument response

The TEAS measurements of the time zero solvent-only scan were recorded to obtain the instrument response function (IRF), which determines the limiting temporal resolution of the present experiments. The value of the temporal resolution was obtained by fitting a Gaussian profile to the time-zero artefact from the solvent-only time zero response, as shown in Fig. S40.

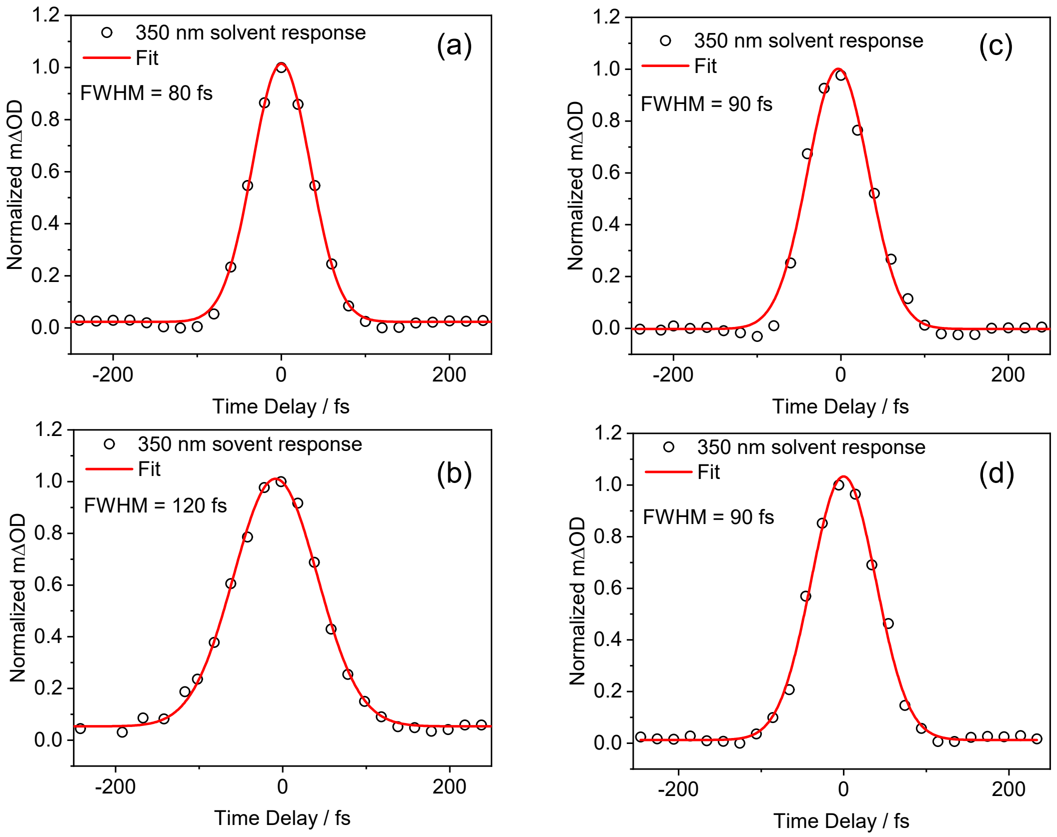

Fig. S40: Solvent-only time-zero response at a probe wavelength of 350 nm for (a-b) DMSO photoexcited at 385 and 404 nm, respectively. (c-d) dioxane photoexcited at 369 and 391 nm, respectively. The excitation wavelengths for the solvents presented herein correspond to the wavelength at which the samples were excited in each solvent. The extracted full-width half maxima are shown in each panel. These values are used as the instrument response in the corresponding global fit analysis of TEA spectra.

I. FTIR measurements and additional TVAS data

The FTIR measurements of CBA and CDBA in DMSO are shown in Fig. S41 and overlaid with the computed wavenumbers reported in Section J. In addition to the TVA spectra collected for CBA and for CDBA in DMSO using a probe pulse centered at 1528 cm^{-1} (reported in the paper), TVA spectra were also measured for CBA and for CDBA in DMSO with probe pulses centred at, respectively, 1704 and 1684 cm^{-1}. These spectra are reported in Fig. S42. The GSB features observed in the TVA spectra of CBA at 1675 and 1737 cm^{-1} and the GSB feature of CDBA at 1667 cm^{-1} were fitted with mono-exponential decay functions, yielding the time constants reported in Table S4. These time constants compare well with the lifetimes assigned to vibrational cooling of S_0 molecules following IC from the S_1 state derived from analysing the corresponding TEA spectra.

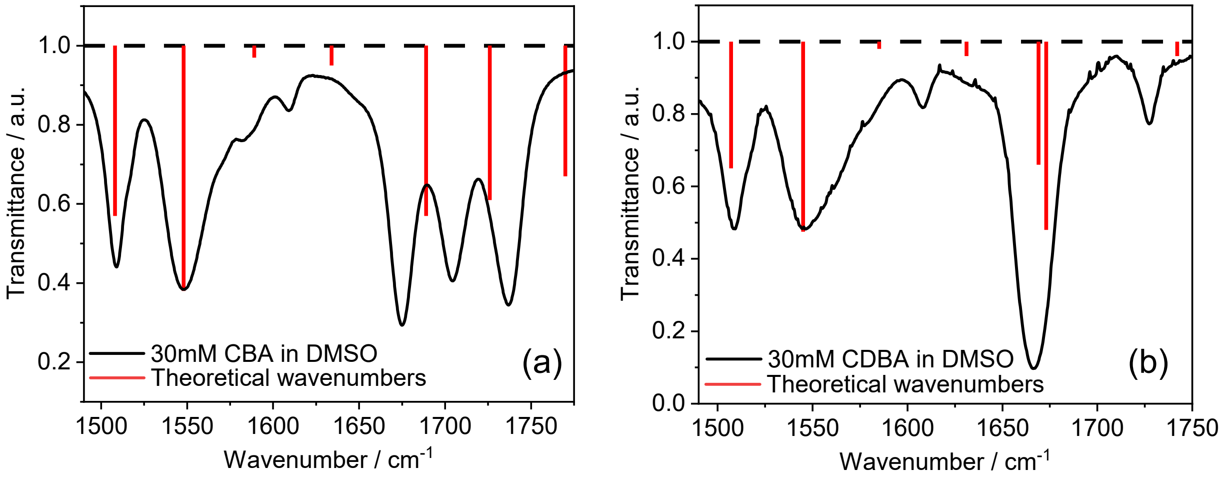

Fig. S41: Steady-state FTIR spectra of 30 mM solutions of (a) CBA and (b) CDBA in DMSO over 1480 to 1760 cm^{-1} region shown as solid black lines. Overlaying the FTIR spectra are wavenumbers (red vertical lines) predicted at the B3LYP/cc-pVDZ level of theory with an implicit DMSO solvent model that are scaled with a scaling factor of 0.985 for CBA and 0.984 for CDBA. The amplitudes of the red vertical lines reflect the IR transition intensities returned by the calculation and scaled to the ~1550 cm^{-1} peak in both molecules.

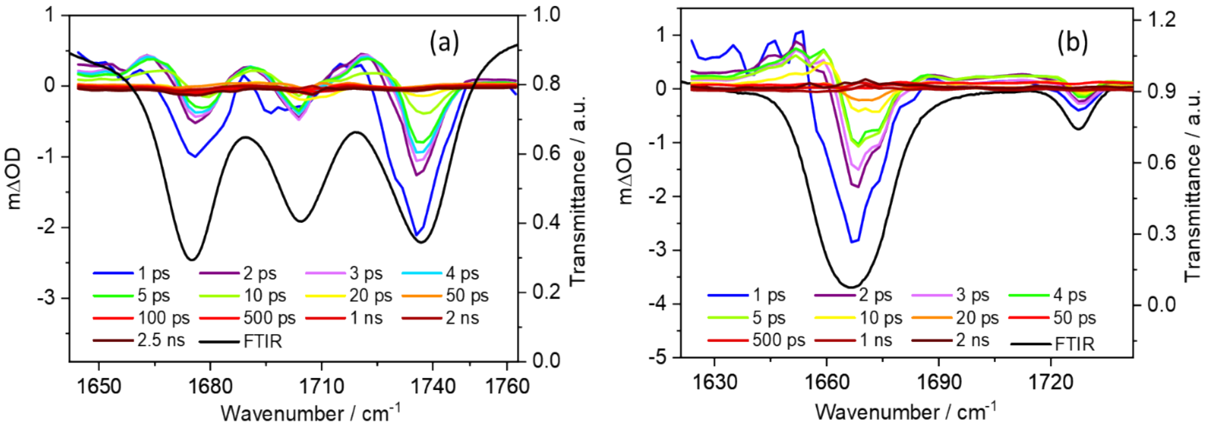

Fig. S42: TVA spectra obtained for 30 mM solutions of (a) CBA and (b) CDBA in DMSO both photoexcited at 385 nm, using probe pulses centred on 1704 and 1682 cm^{-1}, respectively. The TVA spectra are presented as smoothed line plots of mΔOD *vs.* probe wavenumber at selected pump-probe delay times. The steady-state FTIR spectra of CBA/DMSO and CDBA/DMSO are shown as black lines in panels (a) and (b). The scales on the left sides of panels (a) and (b) correspond to the change in optical density for the TVA spectra (coloured lines), while the scale of the right corresponds to the transmittance for the FTIR spectra (black lines).

Table S4: Summary of the time constants and associated errors extracted from TVA spectra collected for CBA and CDBA in DMSO at different wavenumbers.

		**1509 cm^{-1}**	**1548 cm^{-1}**	**1675 cm^{-1}**	**1736 cm^{-1}**
CBA	τ / ps	8.89 ± 0.64	6.27 ± 0.27	6.47 ± 0.79	6.57 ± 0.87
		**1509 cm^{-1}**	**1545 cm^{-1}**	**1667 cm^{-1}**	
CDBA	τ / ps	6.13 ± 0.54	6.10 ± 0.46	5.40 ± 1.04	

J. Computational studies

i. Wavenumber calculations for assigning the FTIR spectra

Wavenumber calculations were carried out at the B3LYP/cc-pVDZ (PCM/DMSO) optimised S_0 geometry, in order to assign vibrational modes to the experimentally observed FTIR bands. The calculated S_0 wavenumbers and the associated vibrational modes are shown in Table S5. A scaling factor was calculated for each predicted spectrum by using one experimental peak as a reference. This scaling was then applied to the calculated S_0 and S_1 wavenumbers so that the calculated wavenumber accurately matched the reference experimental peak. This method has previously been employed for similar systems.[5,6] The selected reference peaks for CBA and CDBA were the 1547 and 1545 cm^{-1} features, respectively, selected because they were peaks of interest in the present study and well characterised computationally. The resultant scaling factors of 0.985 and 0.984 were applied to the S_0 calculated wavenumbers for CBA and CDBA, respectively. As shown in Fig. S41, the calculated strengths of these transitions have also been scaled to match the intensity of the peak at ~1545 cm^{-1}. This confirmed that the calculated peak positions are in good agreement with the experimental peaks.

We highlight here that the FTIR spectra of CBA and CDBA in DMSO show many similarities, but also some variations reflecting the structural difference of the molecules. As shown in Fig. S41, the spectra of CBA revealed three distinct features at higher wavenumbers attributable to C=O stretch and N-H bend modes (see Table S5) while that of CDBA revealed only two features in this region, attributable to the C=O stretching modes. We also note the difference between the calculated and experimental wavenumbers for CDBA in this region. The experimental spectrum shows an intense single feature at 1666 cm^{-1}, while the calculations return two transitions with lower relative intensities at 1676 and 1683 cm^{-1}. We suggest that the more intense experimental peak results from the overlap of the two C=O stretching bands. The FTIR spectra of CBA and CDBA are generally more similar at lower wavenumbers. Both spectra reveal two distinct features associated with a C-H in-plane bending mode of the benzene ring at ~1508 cm^{-1} and the C_2=C_3 stretch of the allylic bond at ~1547 cm^{-1} (see Table S5 for atom numbering and additional vibrational mode assignments for both molecules).

To gain further insight into the relaxation processes of CBA and CDBA in DMSO we also report the S_1 wavenumbers calculated at the ωB97XD/cc-pVDZ (PCM/DMSO) level of theory and the associated vibrational modes in Table S5. Again, scaling factors of 0.977 and 0.970 were calculated and applied to the calculated S_1 wavenumbers using the peaks at 1530 and 1526 cm^{-1} as the reference peaks for CBA and CDBA, respectively. For both molecules, the calculations return a significant shift in the C_2=C_3 stretch wavenumber, from 1547 and 1545 cm^{-1}, respectively, in the S_0 state to 1492 and 1488 cm^{-1}, respectively, in the S_1 state. This suggests that the allylic bond in the excited state has a different character, in line with the conclusion that the S_1 state has TICT character. For the aromatic C-H in-plane bend vibration, the calculated wavenumbers in the S_0 state (1508 and 1507 cm^{-1} for CBA and CDBA, respectively) increase to 1530 and 1526 cm^{-1} in the S_1 state. Therefore, following

$S_1 \leftarrow S_0$ excitation, theory predicts that aromatic C-H in-plane bending mode is shifted to higher wavenumber by 22 cm^{-1} in CBA and by 19 cm^{-1} in CDBA.

Table S5: Computed S_0 and S_1 vibrational wavenumbers (and transition intensities) for CBA and CDBA in DMSO, together with descriptions of the associated modes between 1480 and 1750 cm^{-1}, listed in order of increasing wavenumber. The structures of CBA and CDBA with the atoms numbered accordingly are shown at the head of the Table.

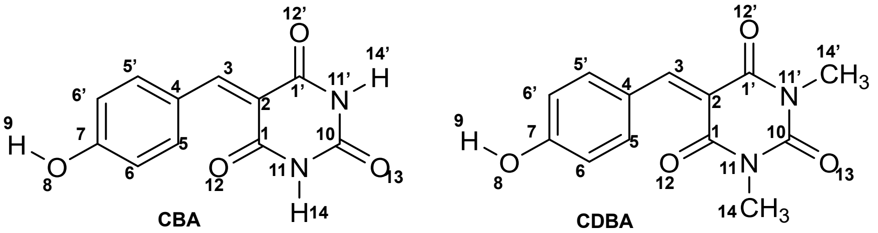

	S_0 wavenumbers /cm^{-1} (IR Int.)	Vibrational mode S_0	S_1 wavenumbers /cm^{-1} (IR Int.)	Vibrational mode S_1
CBA	1508 (926)	C-H in-plane bend (ar) + C=C stretch (ar)	1492 (199)	C_2=C_3 stretch
	1547 (1346)	C_2=C_3 + C_3-C_4 asymmetric stretch	1530 (97)	C-H in-plane bend (ar) + C=C stretch (ar)
	1589 (69)	$C_{6'}$=C_7 + C_4-C_5 symmetric stretch + H_9-O_8-C_7 scissor	1611 (57)	$C_{6'}$=C_7 + C_4-C_5 symmetric stretch + H_9-O_8-C_7 scissor
	1634 (99)	$C_{6'}$-$C_{5'}$ + C_6-C_5 symmetric stretch	1641 (279)	$C_{6'}$-$C_{5'}$ + C_6-C_5 symmetric stretch
	1689 (937)	C_1=O_{12} + $C_{1'}$=$O_{12'}$ asymmetric stretch + N_{11}-H_{14} bend + $N_{11'}$-$H_{14'}$ bend	1699 (560)	C_1=O_{12} + $C_{1'}$=$O_{12'}$ asymmetric stretch + N_{11}-H_{14} bend + $N_{11'}$-$H_{14'}$ bend
	1726 (840)	C_1=O_{12} + $C_{1'}$=$O_{12'}$ symmetric stretch + C_{10}=O_{13} stretch + N_{11}-H_{14} bend + $N_{11'}$-$H_{14'}$ bend	1737 (694)	C_1=O_{12} + $C_{1'}$=$O_{12'}$ symmetric stretch + C_{10}=O_{13} stretch + N_{11}-H_{14} bend + $N_{11'}$-$H_{14'}$ bend
	1770 (711)	C_1=O_{12} + $C_{1'}$=$O_{12'}$ + C_{10}=O_{13} symmetric stretch + N_{11}-H_{14} bend + $N_{11'}$-$H_{14'}$ bend	1800 (843)	C_1=O_{12} + $C_{1'}$=$O_{12'}$ + C_{10}=O_{13} symmetric stretch + N_{11}-H_{14} bend + $N_{11'}$-$H_{14'}$ bend
CDBA	1507 (857)	C-H in-plane bend (ar) + C=C stretch (ar)	1488 (296)	C_2=C_3 stretch
	1545 (1306)	C_2=C_3 + C_3-C_4 asymmetric stretch	1526 (109)	C-H in-plane bend (ar) + C=C stretch (ar)
	1585 (55)	$C_{6'}$=C_7 + C_4-C_5 symmetric stretch + H_9-O_8-C_7 scissor	1604 (37)	$C_{6'}$=C_7 + C_4-C_5 symmetric stretch + H_9-O_8-C_7 scissor
	1631 (98)	$C_{6'}$-$C_{5'}$ + C_6-C_5 symmetric stretch	1631 (288)	$C_{6'}$-$C_{5'}$ + C_6-C_5 symmetric stretch
	1669 (836)	C_1=O_{12} + $C_{1'}$=$O_{12'}$ asymmetric stretch	1665 (454)	C_1=O_{12} + $C_{1'}$=$O_{12'}$ asymmetric stretch
	1673 (1281)	C_1=O_{12} + $C_{1'}$=$O_{12'}$ symmetric stretch	1686 (1054)	C_1=O_{12} + $C_{1'}$=$O_{12'}$ symmetric stretch
	1742 (109)	C_1=O_{12} + $C_{1'}$=$O_{12'}$ + C_{10}=O_{13} symmetric stretch	1757 (296)	C_1=O_{12} + $C_{1'}$=$O_{12'}$ + C_{10}=O_{13} symmetric stretch

ii. Geometries and energies

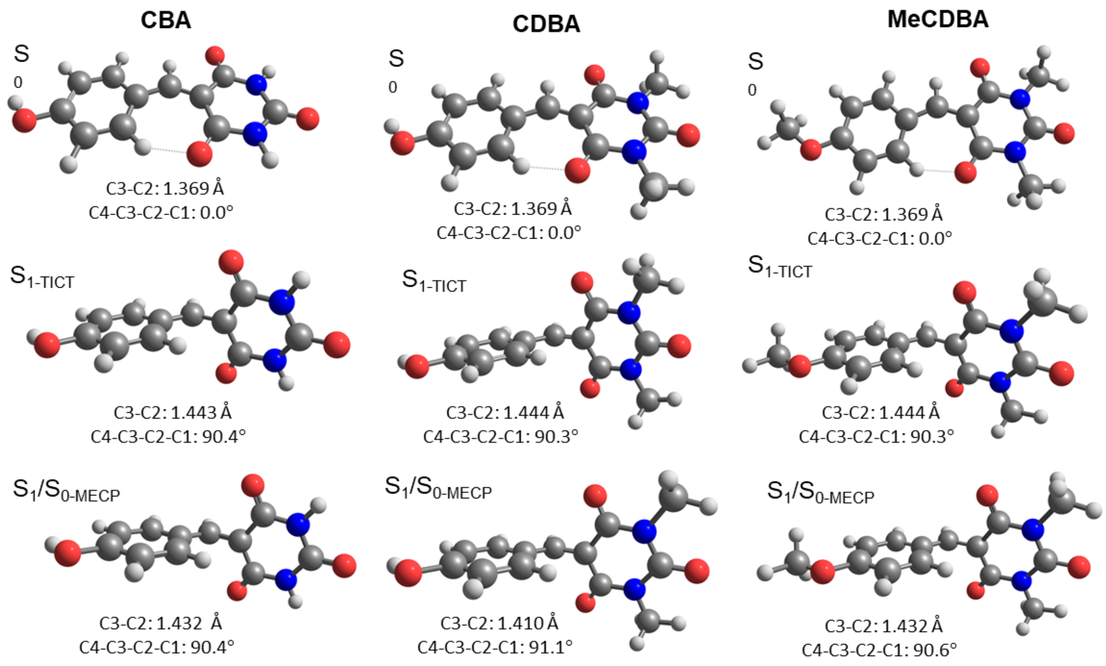

Fig. S43: Optimised geometries for CBA, CBDA, and MeCDBA in the S_0 and S_1 states and at the S_1/S_0 MECP calculated at the ωB97XD/cc-pVDZ level of theory and using a PCM/DMSO solvation model. The main structural differences between the ground and excited state are also indicated (C3–C2 bond lengths in Å and C4–C3–C2–C1 dihedral angle in degrees).

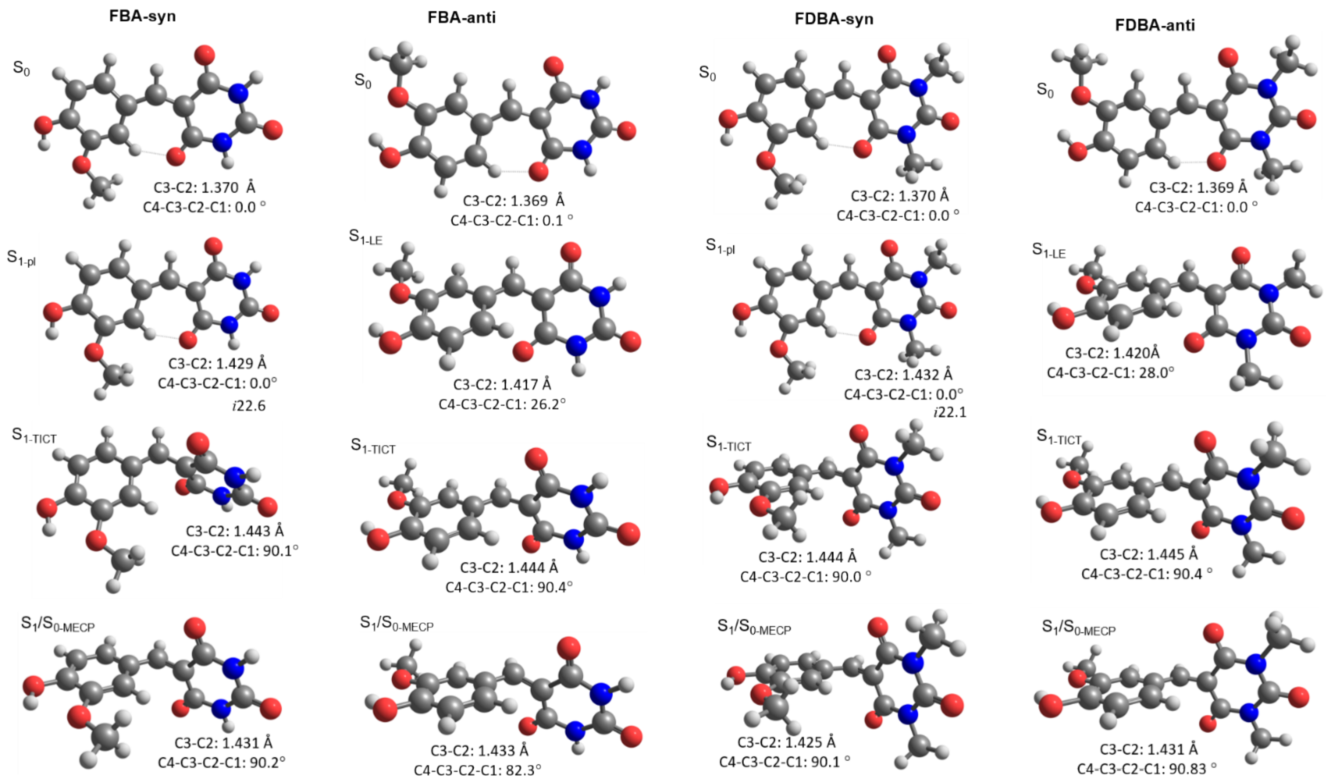

Fig. S44: Optimised geometries for FBA and FBDA in the S_0 and S_1 states and at the S_1/S_0 MECP calculated at the ωB97XD/cc-pVDZ level of theory and using a PCM/DMSO solvation model. The main structural differences between the ground and excited state are also indicated (C3–C2 bond lengths in Å and C4–C3–C2–C1 dihedral angle in degrees). For both FBA and FDBA, the syn isomer is more stable in the ground state by 0.05 eV (1.1 kcal/mol).

Table S6: Vertical excitation energy of S_n state (ΔE_{vert}), vertical emission energy (ΔE_{em}), and adiabatic excitation energy (ΔE_{ad}) calculated with TDDFT (ωB97XD/aug-cc-pVDZ//ωB97XD/cc-pVDZ; PCM/DMSO). Oscillator strengths are given in parentheses.

		Vertical absorption $S_0 \rightarrow S_n$		Vertical emission a) $S_1 \rightarrow S_0$		Adiabatic energy difference b)	
	**State**	**State character**	**ΔE_{vert} / eV**	**State character**	**ΔE_{em} / eV**	**State character**	**ΔE_{ad} / eV**
**CBA**	S_1	$\pi\pi^*$	3.71 (0.963)	$\pi\pi^*_{TICT}$	0.84 (0.000)	$\pi\pi^*_{TICT}$	2.45 (0.000)
	S_2	$n\pi^*$	4.29 (0.000)			$n\pi^*$	4.03 (0.000)
	S_3	$\pi\pi^*$	4.57 (0.008)			$\pi\pi^*$	4.31 (0.019)
						$S_1/S_{0\text{-MECP}}$	2.70
**CBDA**	S_1	$\pi\pi^*$	3.71 (1.014)	$\pi\pi^*_{TICT}$	0.75 (0.000)	$\pi\pi^*_{TICT}$	2.38 (0.000)
	S_2	$n\pi^*$	4.22 (0.000)			$n\pi^*$	3.93 (0.000)
	S_3	$\pi\pi^*$	4.60 (0.007)			$\pi\pi^*$	4.37 (0.018)
						$S_1/S_{0\text{-MECP}}$	2.70
**MeCDBA**	S_1	$\pi\pi^*$	3.67 (1.096)	$\pi\pi^*_{TICT}$	0.77 (0.000)	$\pi\pi^*_{TICT}$	2.40 (0.000)
	S_2	$n\pi^*$	4.23 (0.000)			$n\pi^*$	3.91 (0.104)
	S_3	$\pi\pi^*$	4.59 (0.006)			$\pi\pi^*$	4.34 (0.014)
						$S_1/S_{0\text{-MECP}}$	2.60
**FBA-anti**	S_1	$\pi\pi^*$	3.52 (0.783)	$\pi\pi^*_{LE}$	2.51 (0.327)	$\pi\pi^*_{LE}$	3.00 (0.327)
				$\pi\pi^*_{TICT}$	0.85 (0.000)	$\pi\pi^*_{TICT}$	2.42 (0.000)
	S_2	$\pi\pi^*$	4.21 (0.164)			$\pi\pi^*$	3.93 (0.318)
	S_3	$n\pi^*$	4.28 (0.000)			$n\pi^*$	4.01 (0.000)
						$S_1/S_{0\text{-MECP}}$	2.64
**FBA-syn**	S_1	$\pi\pi^*$	3.53 (0.835)	$\pi\pi^*_{LE}$	3.18 (0.843)	$\pi\pi^*_{LE}$	3.34 (0.843)
				$\pi\pi^*_{TICT}$	0.89 (0.000)	$\pi\pi^*_{TICT}$	2.48 (0.000)
	S_2	$\pi\pi^*$	4.17 (0.094)				
	S_3	$n\pi^*$	4.31 (0.000)				
						$S_1/S_{0\text{-MECP}}$	2.77
**FDBA-anti**	S_1	$\pi\pi^*$	3.54 (0.842)	$\pi\pi^*_{LE}$	2.49 (0.347)	$\pi\pi^*_{LE}$	2.99 (0.347)
				$\pi\pi^*_{TICT}$	0.75 (0.000)	$\pi\pi^*_{TICT}$	2.35 (0.000)
	S_2	$n\pi^*$	4.21 (0.000)			$n\pi^*$	3.94 (0.000)
	S_3	$\pi\pi^*$	4.24 (0.154)			$\pi\pi^*$	3.98 (0.293)
						$S_1/S_{0\text{-MECP}}$	2.56
**FDBA-syn**	S_1	$\pi\pi^*$	3.55 (0.893)	$\pi\pi^*_{LE}$	3.18 (0.908)	$\pi\pi^*_{LE}$	3.35 (0.908)
				$\pi\pi^*_{TICT}$	0.79 (0.000)	$\pi\pi^*_{TICT}$	2.41 (0.000)
	S_2	$\pi\pi^*$	4.21 (0.086)				
	S_3	$n\pi^*$	4.24 (0.000)				
						$S_1/S_{0\text{-MECP}}$	2.70

a) Vertical emission energy: energy difference between the optimised S_1 state and the S_0 energy at the optimised S_1 geometry.
b) Adiabatic energy: energy difference between the optimised S_n state and the optimised S_0 state.

We note in Table S6 that for the coumaryl series, S_2 is a $^1n\pi^*$ state, and S_3 the $2^1\pi\pi^*$ state with a small oscillator strength. For the ferulyl series, the $2^1\pi\pi^*$ state is stabilised due to the increase of the conjugation, which makes the S_2 and S_3 states near-degenerate. They can therefore appear in reverse order (i.e. S_2 as $2^1\pi\pi^*$ and S_3 as $^1n\pi^*$).

Additional calculations were carried out to evaluate how phenyl ring substitution might affect the absorption spectra. Starting from $R_1 = R_2 = R_3 = H$ (See Scheme 1 within manuscript for the positions of the various R groups) here called CinBA, progressive –OH and/or –OCH_3 additions cause a relative stabilisation of the S_1 $\pi\pi^*$ state due to the increase of the conjugation, which explains the observed red-shifts in the absorption spectra. The ΔE_{vert} values computed for the series CinBA – CBA – FBA – SBA are, respectively, 3.98 – 3.71 – 3.53 – 3.44 eV.

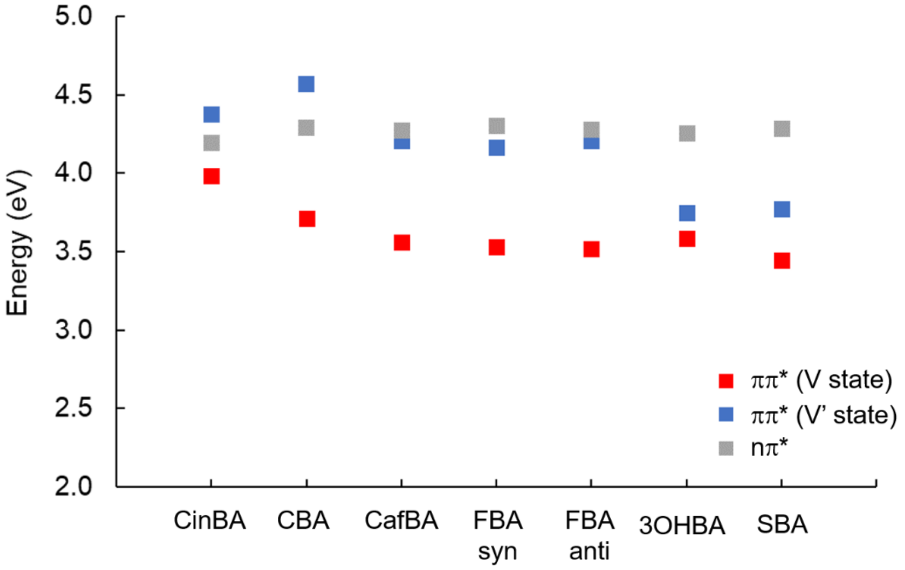

Derivatives	R_1	R_2	R_3
**CinBA**	H	H	H
**CBA**	OH	H	H
**CafBA**	OH	OH	H
**FBA-syn**	OH	OCH_3	H
**FBA-anti**	OH	H	OCH_3
**3-OHBA**	OH	OH	OH
**SBA**	OH	OCH_3	OCH_3

Fig. S45: Vertical excitation energies showing how different substituents to the phenyl ring affect the electronic absorption spectra, calculated with TDDFT (ωB97XD/aug-cc-pVDZ//ωB97XD/cc-pVDZ; PCM/DMSO).

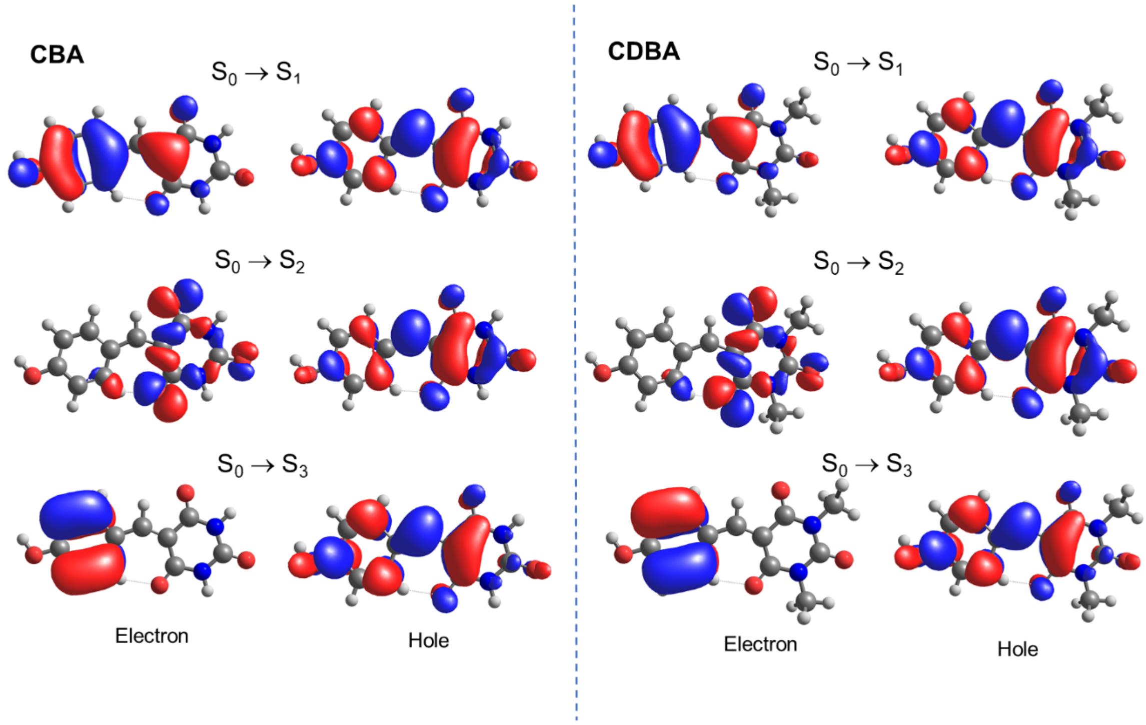

Fig. S46: Natural transition orbitals (NTOs) for the three lowest excitations of CBA (left) and CDBA (right) calculated at ωB97XD/aug-cc-pVDZ//ωB97XD/cc-pVDZ using PCM/DMSO. The NTOs for MeCDBA are analogous.

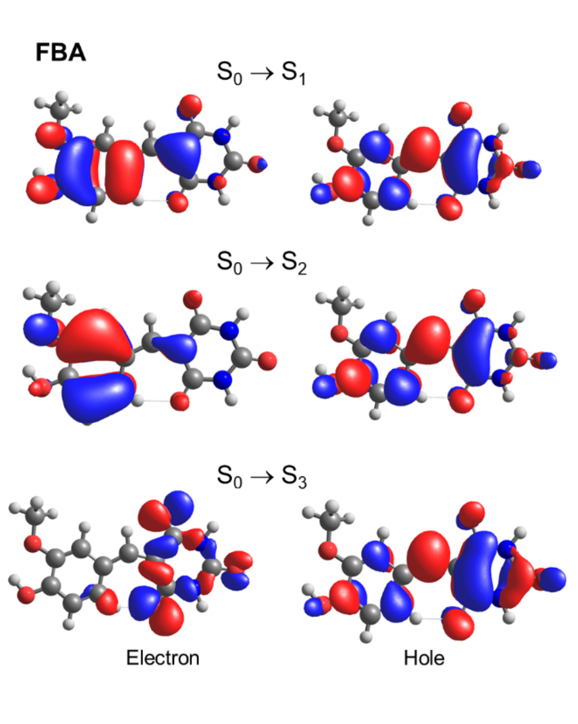

Fig. S47: Natural transition orbitals for the three lowest excitations of FBA (left) and FDBA (right) calculated at ωB97XD/aug-cc-pVDZ//ωB97XD/cc-pVDZ using PCM/DMSO. The NTOs for the *anti* conformer are shown, but those for *syn*-FBA are analogous.

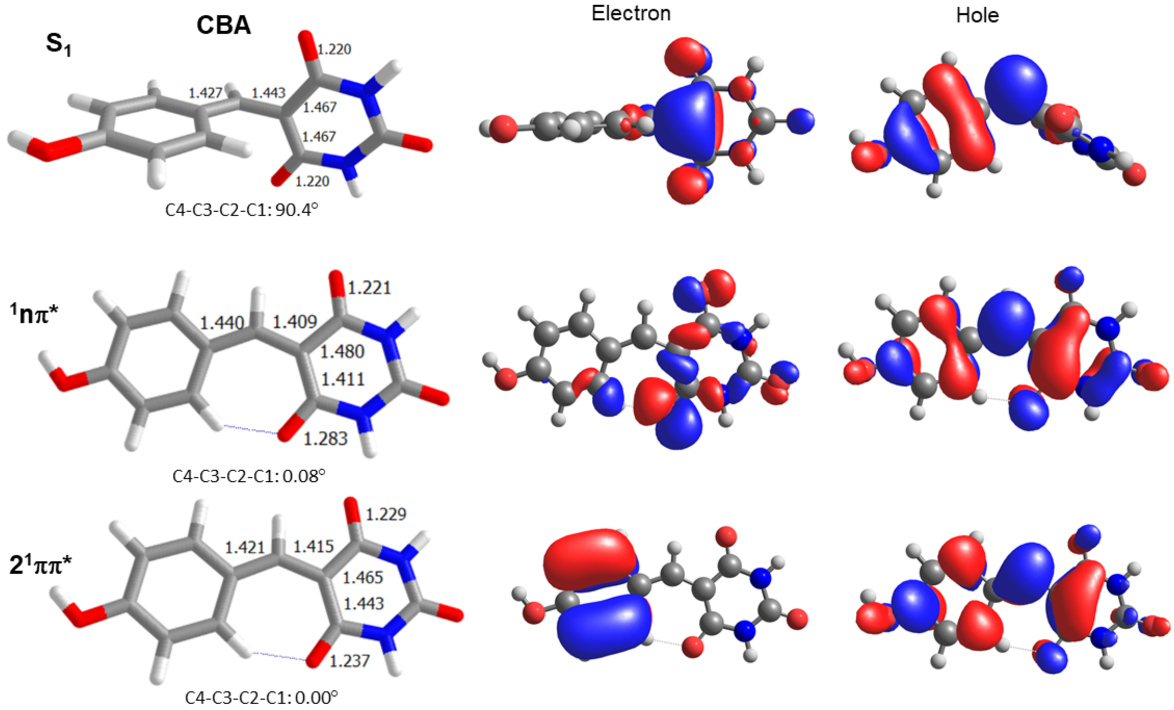

Fig. S48: Geometries and natural transition orbitals which characterise the S_1, S_2 and S_3 states of CBA calculated at ωB97XD/aug-cc-pVDZ//ωB97XD/cc-pVDZ using PCM/DMSO. Geometries and NTOs for CDBA are analogous.

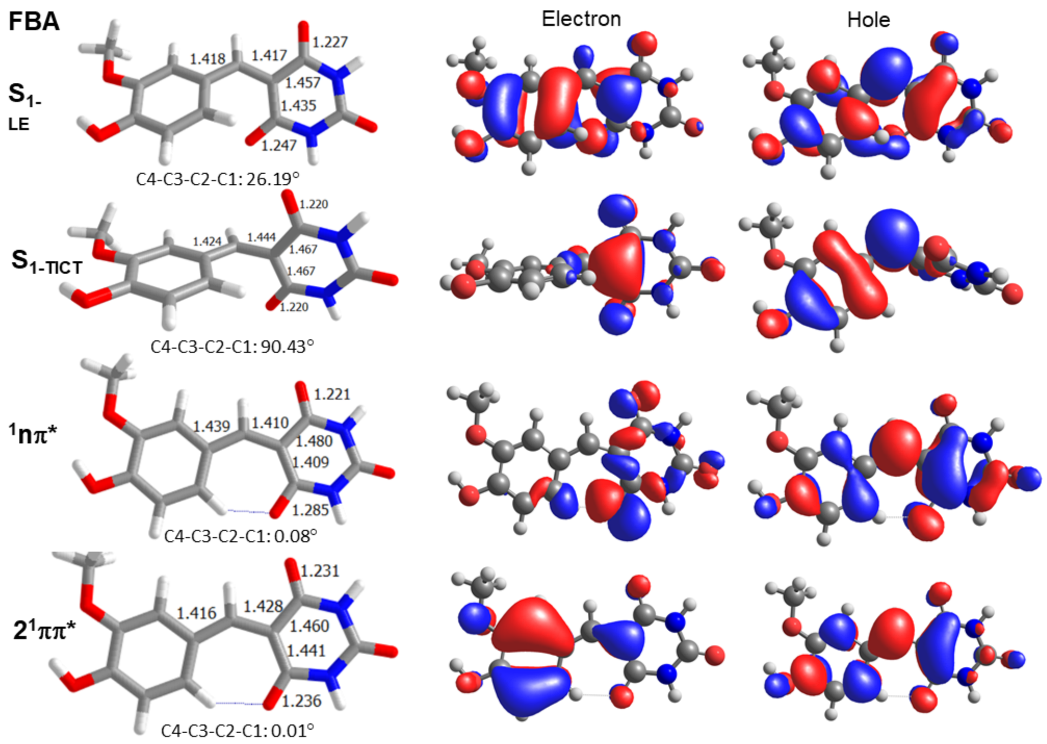

Fig. S49: Geometries and natural transition orbitals characterising the S_1, S_2 and S_3 states of FBA calculated at ωB97XD/aug-cc-pVDZ//ωB97XD/cc-pVDZ using PCM/DMSO. Geometries and NTOs for FDBA are analogous.

iii. Characterisation of the states in terms of charge transfer character

To evaluate the charge-transfer (CT) character of the three lowest singlet excited states, *q*(CT) numbers were computed. These are shown in Table S7. *q*(CT) numbers are calculated as partial summations over squared transition density matrix elements of molecular fragments and range from 0 to 1. The closer to one, the larger the charge-transfer character of the state; the closer to zero, the smaller the CT character (*i.e.*, such a state has a locally-excited (LE) state character).

The vertically excited S_1 state has a *q*(CT) number ~0.4, while *q*(CT) for the $S_{1\text{-LE}}$ state is slightly larger. This means that at these points of the potential energy surface, the S_1 state is better descrbed as having localised excited (LE) state character (though, clearly with some CT character). The $S_{1\text{-TICT}}$ configuration, in contrast, has a *q*(CT) value > 0.8, which taken together with the ~90° twist, is compelling evidence of strong charge-transfer character. The plot of the electronic density difference between the S_0 and S_1 states in the S_0 and $S_{1\text{-TICT}}$ geometries (Fig. S50) clearly shows that, after the vertical excitation, the higher electronic density is located predominantly at the donor region (the phenyl ring). Upon S_1 relaxation to the twisted minimum, however, a clear charge separation between the donor and acceptor groups is evident, with the electronic density now mostly located in the acceptor part (the barbituric ring) of the molecule.

Table S7: *q*(CT) numbers obtained at TDDFT/ωB97XD/cc-pVDZ PCM/DMSO. The figures above the table show how the molecules were divided in fragments for computing the CT number.

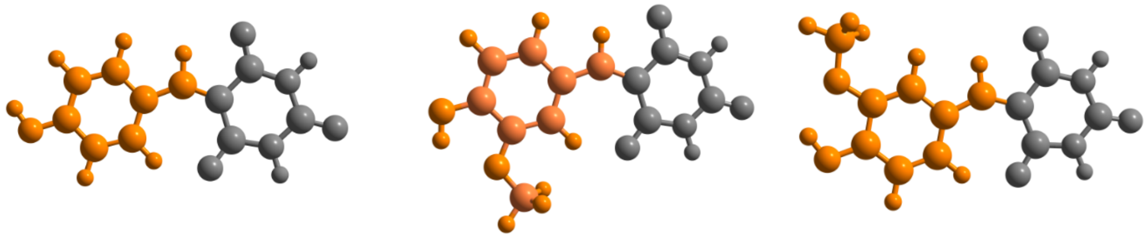

	CBA			FBA-syn				FBA-anti			
	Geom S_0	Geom $S_{1\text{-TICT}}$	Geom S_1/S_0	Geom S_0	Geom $S_{1\text{-LE}}$	Geom $S_{1\text{-TICT}}$	Geom S_1/S_0	Geom S_0	Geom $S_{1\text{-LE}}$	Geom $S_{1\text{-TICT}}$	Geom S_1/S_0
S_1	0.423	0.832	0.804	0.396	0.441	0.836	0.802	0.397	0.391	0.832	0.810
S_2	0.471	0.902	0.860	0.475	0.499	0.902	0.853	0.313	0.298	0.898	0.866
S_3	0.262	0.901	0.869	0.309	0.368	0.074	0.099	0.473	0.542	0.073	0.103

S_0 geometry $S_{1\text{-TICT}}$ geometry

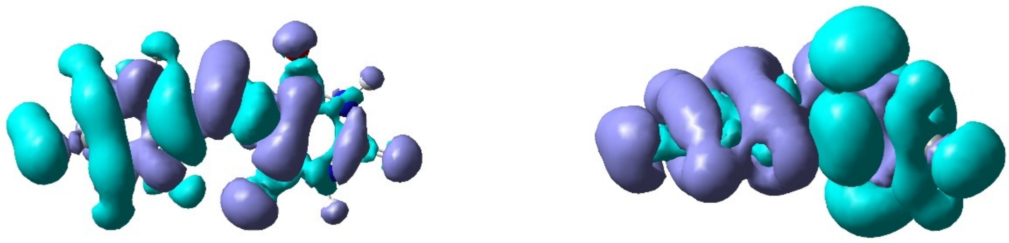

Fig. S50: Electronic density difference between S_0 and S_1 states in the S_0 and $S_{1\text{-TICT}}$ geometries for CBA. Green: negative; Purple: positive. For clarification, the CBA structure (and orientation) is reproduced as ball and stick in Table S7.

iv. Linear interpolations

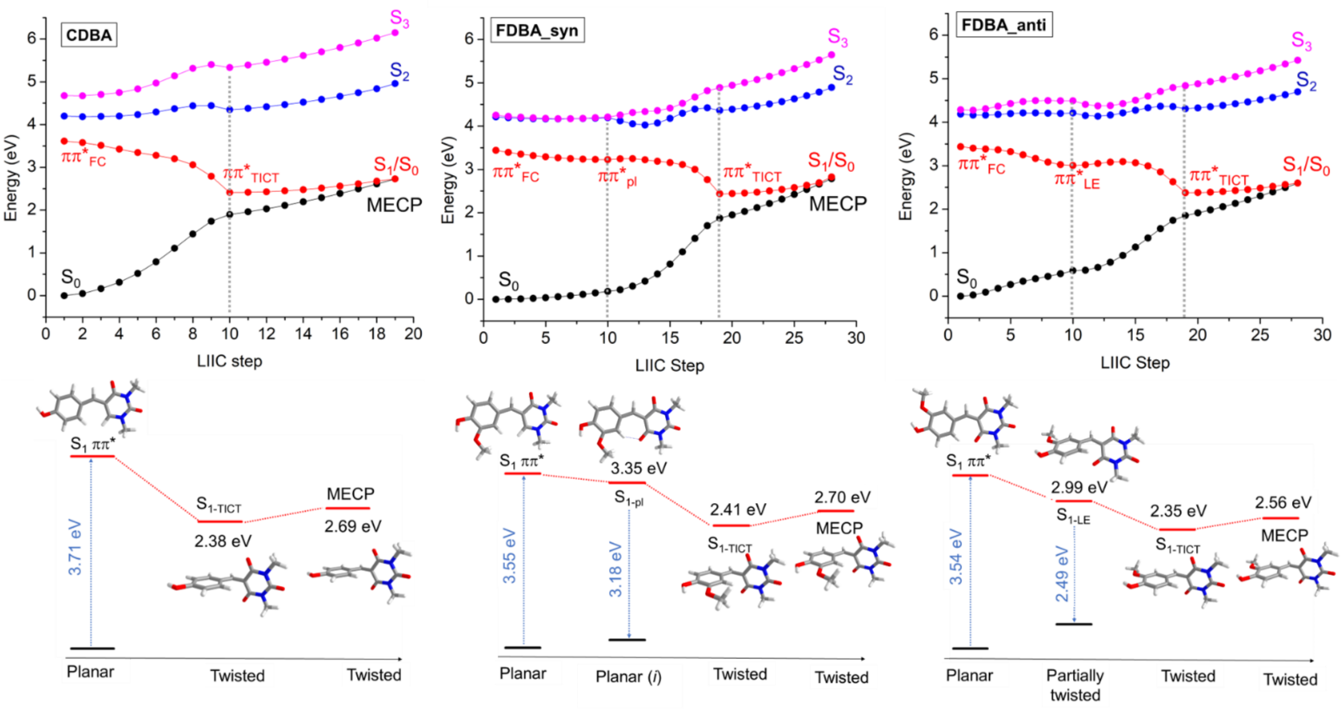

Fig. S51: Potential energy curves (PECs) calculated at TD-ωB97XD/cc-pVDZ level using the PCM/DMSO implicit solvation model for CDBA, *anti*-FDBA and *syn*-FDBA. The *syn*-FDBA isomer is 0.05 eV (1.1 kcal/mol more stable than *anti*-FDBA in the S_0 state). Top: For CDBA: LIIC starting from S_1 state following vertical excitation from S_0 state equilibrium geometry to the $S_{1\text{-TICT}}$ min (shown up to the dashed grey line) and from $S_{1\text{-TICT}}$ to S_1/S_0 MECP. For FDBA: LIIC from S_1 vertically excited to the $S_{1\text{-LE}}$ (shown up to the first dashed grey line), from $S_{1\text{-LE}}$ to the $S_{1\text{-TICT}}$ (up to the second dashed grey line), and from $S_{1\text{-TICT}}$ to S_1/S_0 MECP. Bottom: Energy diagrams showing the stationary points and respective energies corrected using the aug-cc-pVDZ basis set. The steric interactions in the *syn* conformer seem to promote a “torque” in the S_1 excited state, enhancing the tendency of twisting around the central C=C bond. For MeCDBA, the PECs and energy diagrams are similar to CDBA. The energies of the stationary points are given in Table S6.

v. Solvent effects

The calculated adiabatic energies for CBA and FBA using 1,4-dioxane return energies that are smaller than those for DMSO, contrary to what might traditionally be expected for TICT states.[7] To obtain further insight, we have investigated the effect of adding one DMSO molecule to our PCM calculations and considered the effect of state-specific[8] (SS) solvation (Tables S8 and S9).

To check if the explicit hydrogen bond (HB) interaction of DMSO with the hydroxyl group in the chromophore would affect the positions of potential minima, we have performed additional LR-PCM/TDDFT calculations, including one DMSO molecule. Fig. S52 shows several hydrogen bonded FBA-DMSO conformers and their relative energies. The “open” conformers, where the intramolecular HB with the methoxy group is broken, and where the intermolecular HB with DMSO is favoured, are more stable. For these conformers, no minimum associated with a protonated DMSO was found in the ground or first excited singlet states. The *syn* conformer is again more stable than the *anti* conformer, by >1.0 kcal mol^{-1} in the S_0 state. Overall, the results reveal only very mild influences of HB interactions, although a small decrease in ΔE_{vert} and a small increase in the adiabatic energies of $S_{1\text{-TICT}}$ and S_1/S_0 were observed.

The state-specific (SS) solvation approach allows the dynamic component of the solvent polarisation to rearrange and become equilibrated with the new charge distribution of the solute in a specific excited state after excitation.[8] Using the SS solvation approach, the adiabatic energies of TICT and MECP geometries computed with DMSO dropped below those computed with 1,4-dioxane, while the $S_1 \leftarrow S_0$ and $S_{1\text{-LE}} \rightarrow S_0$ energies were almost unchanged (reflecting the localised character of these transitions). Significantly, the SS approach does not change the interpretation of the experimental time constants based on the previous PCM calculations (using the default Linear Response (LR) approach). In fact, the energy barriers towards the MECP computed within the LR and SS approaches are almost the same. As Table S9 shows, in all cases investigated here, the barrier to IC is smaller in 1,4-dioxane than in DMSO, consistent with the shorter τ_2 lifetimes found experimentally in the former solvent.

Table S8: Comparisons of the effects of different solvation schemes: LR-PCM or SS-PCM using DMSO as an implicit solvent; and microsolvated (1 DMSO molecule + LR-PCM/DMSO). DFT/MRCI energies (gas phase) computed on the top of LR-PCM/DMSO geometries are also shown. . All TDDFT calculations were performed using ωB97XD/aug-cc-pVDZ//ωB97XD/cc-pVDZ, while the DFT/MRCI calculations employed BHLYP/def2-TZVP.

	**TDDFT (LR-PCM)**			**DFT/MRCI**		**TDDFT (LR-PCM) + 1 DMSO**		**TDDFT (SS-PCM)**	
	**CBA**	**FBA anti**	**FBA syn**	**FBA anti**	**FBA syn**	**FBA anti**	**FBA syn**	**FBA anti**	**FBA syn**
**S_1 vert**	3.71	3.52	3.53	3.34	3.34	3.38	3.40	3.44	3.50
**$S_{1\text{-LE}}$**	NF	3.00	3.34	2.89	3.20	2.93	NF	2.95	3.26
**$S_{1\text{-TICT}}$**	2.45	2.42	2.48	2.28	2.30	2.44	2.51	2.02	2.03
**S_1/S_0**	2.70	2.64	2.77	2.58	2.65	2.88	2.89	2.31	2.40
**Barrier IC**	0.25	0.23	0.30	0.30	0.35	0.44	0.39	0.29	0.37

Table S9: Energies of selected critical points calculated using different solvation schemes: LR-PCM or SS-PCM, with 1,4-dioxane as implicit solvent. TDDFT calculations were performed using ωB97XD/aug-cc-pVDZ//ωB97XD/cc-pVDZ.

	**TDDFT (LR-PCM)**			**TDDFT (SS-PCM)**
	**CBA**	**FBA anti**	**FBA syn**	**FBA syn**
**S_1 vert**	3.75	3.55	3.56	3.56
**$S_{1\text{-LE}}$**	NF	3.05	3.38	3.42
**$S_{1\text{-TICT}}$**	2.30	2.28	2.36	2.20
**S_1/S_0**	2.34	2.36	2.44	2.25
**Barrier IC**	0.04	0.08	0.08	0.05

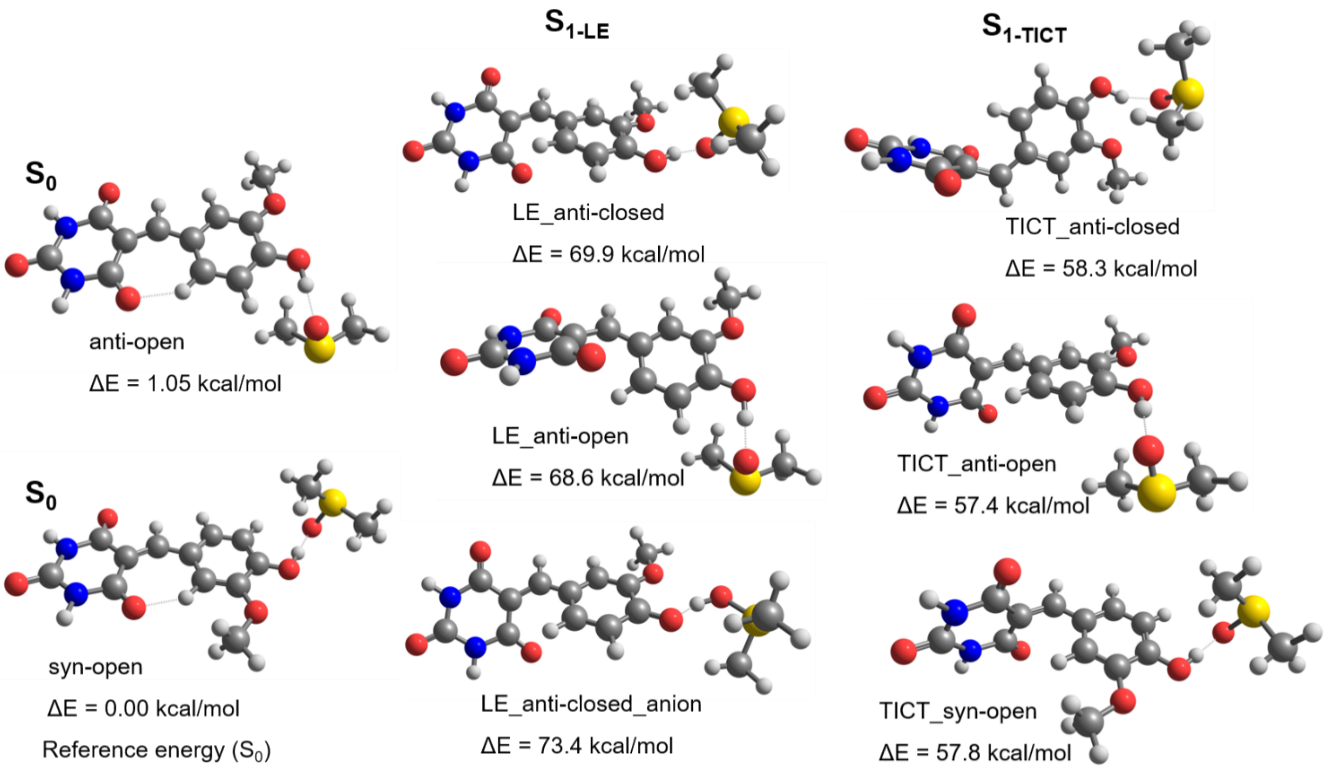

Fig. S52: Optimised geometries for FBA including one DMSO molecule in the S_0 and S_1 states calculated at ωB97XD/cc-pVDZ using PCM/DMSO. The energy differences (in kcal mol^{-1}) relative to the most stable conformer in the ground state (shown at the lower left) are also given.

vi. DFT/MRCI calculations

To obtain further insights into the ESA features observed in the TEAS experiments, the absorption spectrum from the S_1 state was computed using the combined density functional theory and multireference configuration interaction (DFT/MRCI) method.[9,10] This method accommodates a major part of the dynamic correlation coming from DFT while also including static correlation effects by considering a short configuration interaction (CI) expansion, and allows computation of transition dipole moments between excited states. Twenty roots were computed using the S_1 state density as a reference to obtain the oscillator strengths for transitions between this state and higher-lying singlet excited states. Since the DFT/MRCI program does not have energy gradients implemented, geometry optimisations are not available with this method. Single point calculations were performed in the gas phase for each of the LIIC points obtained using TDDFT (ωB97XD/cc-pVDZ, PCM/DMSO) – shown previously in Figure S51. These points connect the ground state geometry (termed the 'vertically excited' S_1 state), the various S_1 stationary points and the S_1/S_0 MECP for each of CBA, *syn*- and *anti*-FBA. The DFT/MRCI Hamiltonian was computed with the BH-LYP[11-13] functional using the modified set of parameters developed in Grimme's group[14] (p_1 = 0.629, p_2 = 0.611, p_J = 0.119, $p[0]$ = 8.000, α = 0.503) and the def2-TZVP[15] basis set. The CI space was selected using a threshold parameter (δE_{sel}) of 1.0 Hartree and a reference space that included all possible configurations generated by (up to) doubly exciting 10 electrons within 10 orbitals. The DFT/MRCI code was used along with the DFT wavefunctions obtained with Turbomole v.7.5.[16] Vertical excitations and adiabatic

energies for the first few low-lying excited states for CBA and FBA were also collected at the DFT/MRCI level at the optimised TDDFT geometries.

Transitions between the S_1 state and 18 states lying above it in energy were computed along the LIIC for CBA and both FBA conformers to identify higher excited states with non-negligible oscillator strengths for transitions that could coincide with the experimentally observed ESA features. These results are presented in Figs. S53 and S54, wherein each point represents an interpolated geometry on the S_1 surface and its colour intensity provides an indication of the magnitude of the respective $S_n \leftarrow S_1$ oscillator strengths. The S_1 state is represented by the dashed line at zero; positive values thus represent transitions from S_1 to higher excited states while the negative energies in each Figure represent the vertical $S_1 \leftrightarrow S_0$ energy gap at different geometries along the LIICs. Thus the first point in each plot is associated with vertical excitation to the S_1 state. As the dihedral angle starts to twist, the energy gap closes, and the $S_1 \leftrightarrow S_0$ oscillator strength vanishes by the geometry of the $S_{1\text{-TICT}}$ state.

The positive energies are associated with absorption from the S_1 state, and the grey points represent excited states with zero oscillator strength. For geometries associated with the first LIIC points, excited states are identified at energies ~3-3.5 eV with relatively large oscillator strengths. These could be associated with the ESA observed at λ~350 nm (shown as a dashed line at 3.5 eV). As the twisting angle tends towards 90 degrees, another state with smaller oscillator strength starts to appear at E~2.8 eV. For all molecules investigated, at the $S_{1\text{-TICT}}$ minimum (corresponding to point 10 for CBA, Fig. S53, and point 19 for FBA, Fig. S54), the calculations identify a state at E~2.8 eV above the S_1 state with oscillator strength f ~0.02-0.03. This could contribute to the weak ESA feature observed at λ~450 nm but the observed blue-shift of this feature favours assignment as a hot ground state absorption (HGSA). Note that, at the DFT/MRCI level, the S_1/S_0 MECP is reached before the last point of the LIIC (for CBA, for example, the MECP falls at point 13), as indicated by the overlap of the dashed line at 0 eV (S_1 state) and the grey bullet point representing the S_0 state. This meansthat, after this point, the oscillator strengths and energies returned by the calculations represent absorption from the highly distorted ground state due to the switch of root 1 (S_0) and root 2 (S_1) in DFT/MRCI calculations.

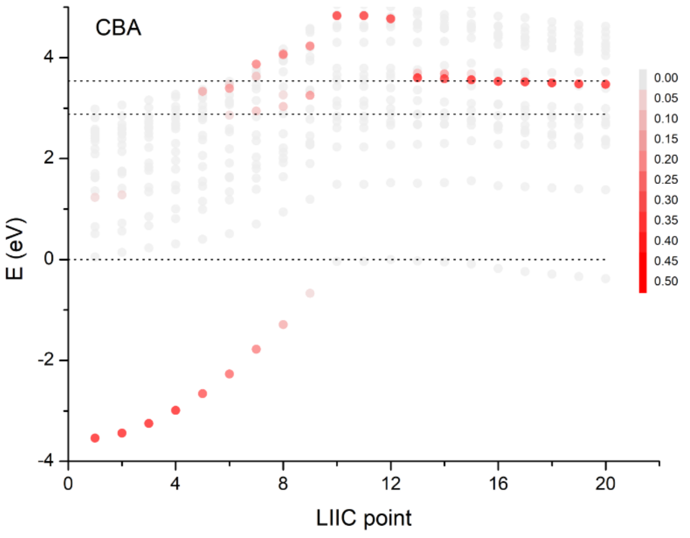

Fig. S53: Single point energies and oscillator strengths computed for CBA using DFT/MRCI along the LIIC points shown in Fig. 4 of the manuscript. The dashed line at 0 eV represents the S_1 state. The energies of all other states is given taking S_1 state as reference. Therefore,negative energies represent $S_1 \rightarrow S_0$ vertical energy gaps and associated oscillator strengths (shown using an intensity scale such that the most intensely coloured points represent transitions of highest oscillator strength) and positive energies represent $S_n \leftarrow S_1$ vertical energy gaps. The dashed lines at E = 2.8 and 3.5 eV indicate the experimentally observed ESA features.

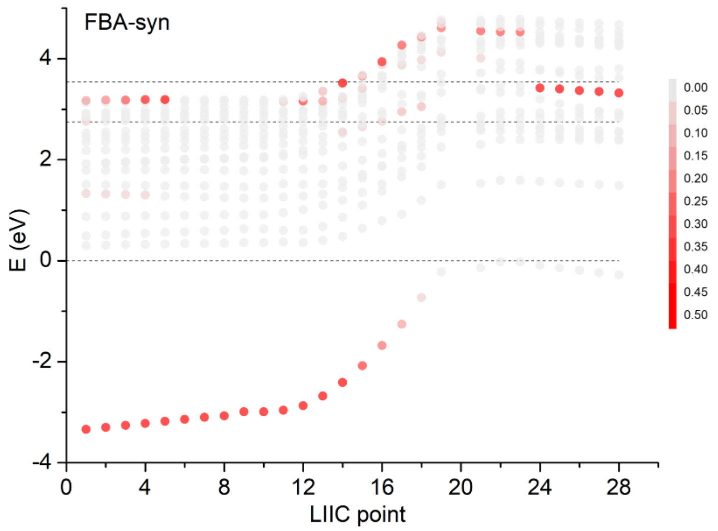

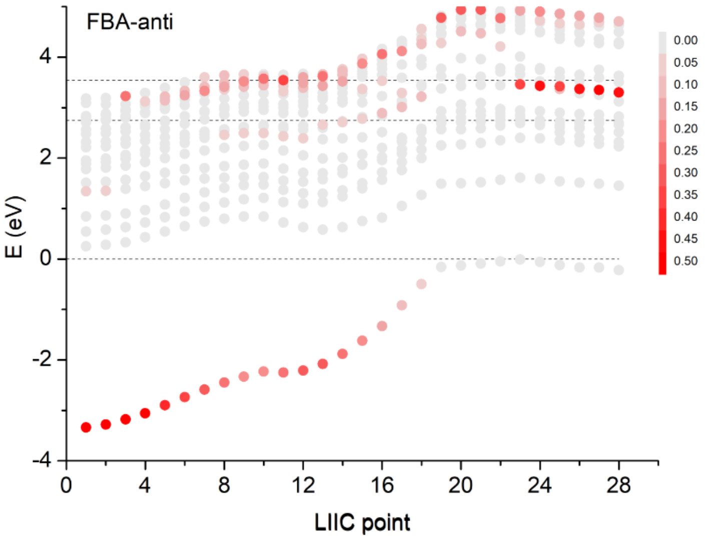

Fig. S54: Single point energies and oscillator strengths computed for syn- and anti-FBA using DFT/MRCI along the LIIC points shown in Fig. 4 of the manuscript. The dashed line at 0 eV represents the S_1 state. The energies of all other states is given taking S_1 state as reference. Therefore, negative energies represent $S_1 \rightarrow S_0$ vertical energy gaps and associated oscillator strengths (shown using an intensity scale such that the most intensely coloured points represent transitions of highest oscillator strength) and positive energies represent $S_n \leftarrow S_1$ vertical energy gaps. The dashed lines at E = 2.8 and 3.5 eV indicate the experimentally observed ESA features.

K. Antiradical activities

The antiradical activities of the barbiturics are reported in Table S10, while an example of the analysis for CafBA is reported in Figure S55. This figure shows the percentage curves of %DPPH and %reduced DPPH plotted in Regressi® software using an average of the last six points. The amount of solute needed to reduce the initial number of DPPH free radicals by half, i.e., EC_{50}, was provided by the crossing point of %DPPH and %reduced DPPH.

Table S10: Antiradical activity of the barbituric acid, commercially available antioxidants, and the barbituric studied herein.

Compounds	EC_{50} (nmol)	Standard Deviation (SD)	Standard Error of Mean (SEM)
**Barbituric Acid (BA)**	2.8	0.1	0.1
**Dimethyl Barbituric Acid (DBA)**	3.3	0.2	0.1
**Butyl HydroxyAnisole (BHA)**	42	0.1	0.1
**Butyl HydroxyToluene (BHT)**	4.1	0.1	0.1
**Coumaryl Barbituric Acid (CBA)**	18.7	0.5	0.3
**Ferulyl Barbituric Acid (FBA)**	12.0	0.3	0.2
**Caffeyl Barbituric Acid (CafBA)**	2.2	0.0	0.0
**Sinapyl Barbituric Acid (SBA)**	3.4	0.1	0.1
**4-Methoxy Coumaryl Barbituric Acid (MeCBA)**	16.3	2.7	1.9
**Coumaryl Dimethyl Barbituric Acid (CDBA)**	13.4	4.4	3.1
**Ferulyl Dimethyl Barbituric Acid (FDBA)**	10.6	0.5	0.3
**Caffeyl Dimethyl Barbituric Acid (CafDBA)**	2.1	0.1	0.1
**Sinapyl Dimethyl Barbituric Acid (SDBA)**	3.4	0.0	0.0
**4-Methoxy Coumaryl Dimethyl Barbituric Acid (MeCDBA)**	6.3	0.5	0.4

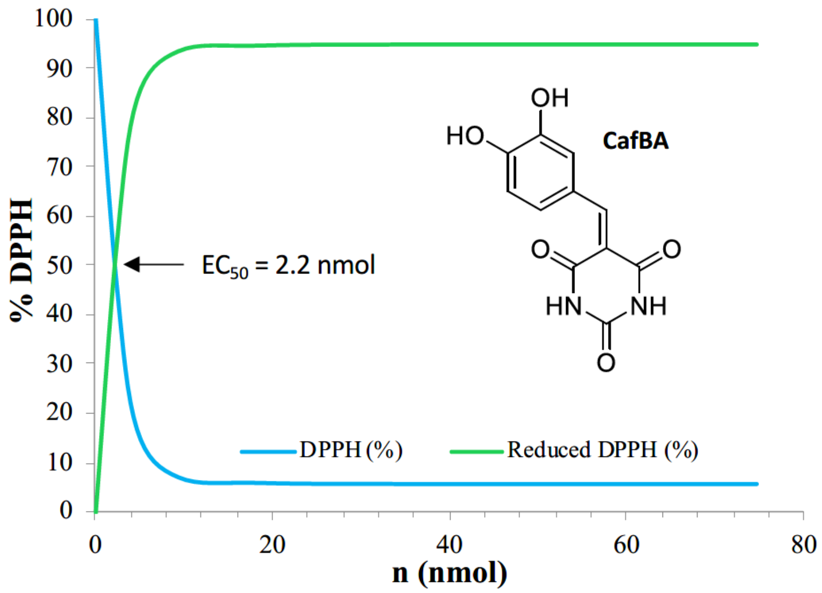

Fig. S55: Schematic representation of the antiradical activity analysis for CafBA via 2,2-diphenyl-1-picrylhydrazy (DPPH) assay. The EC_{50} is provided by the crossing point of % DPPH (blue) and % reduced DPPH (green), which occurs at 2.2 nmol.

L. Additional *in silico* toxicology prediction results

The mutagenicity and carcinogenicity predicted scores are shown in Fig. S56. The output for the barbituric derivatives endocrine toxicity predictions using the VEGA platform are reported in Table S11. In Table S12, the results of the acute and short-term toxicity are reported. The read-across analysis is reported in Table S13, while the compounds used for this analysis are reported in Figure S57. None of the barbiturics of interest is predicted to have the potential for acute toxicity, genotoxicity or carcinogenicity.

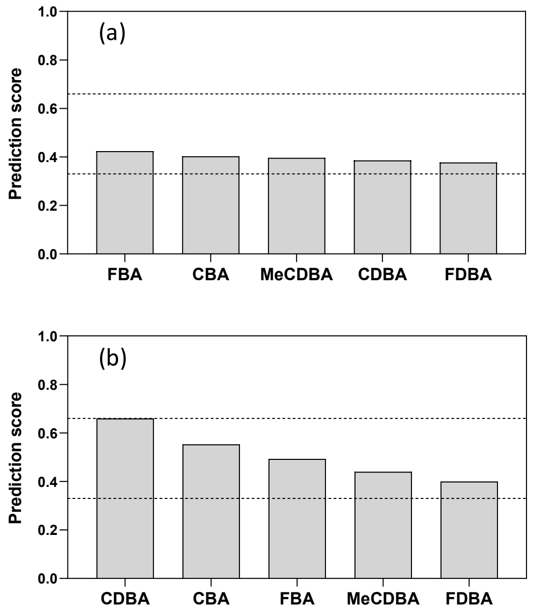

Fig. S56: Schematic presentation of the results of the *in silico* analysis with regard to (a) endpoint mutagenicity and (b) carcinogenicity. The test compounds are listed by their average prediction score on the y-axis. As discussed in section A, the prediction scores are divided into three groups: the probable mutagens/carcinogens with scores >0.66, the probable non-mutagens/non-carcinogens with scores <0.33 and the remaining equivocal predictions with scores in the range 0.33 -0.66.

Table S11. Predictions regarding endocrine toxicity using the VEGA platform.

	CBA		CDBA		MeCDBA		FBA		FDBA	
**VEGA computational model**	**Prediction**	**Reliability**	**Prediction**	**Reliability**	**Prediction**	**Reliability**	**Prediction**	**Reliability**	**Prediction**	**Reliability**
Estrogen Receptor Relative Binding Affinity	active	low	inactive	moderate	inactive	moderate	active	low	inactive	low
Estrogen Receptor-mediated effect	inactive	good	inactive	good	inactive	good	inactive	good	inactive	good
Androgen Receptor-mediated effect	active	low	active	low	active	moderate	active	moderate	active	moderate
Thyroid Receptor Alpha effect	inactive	good	inactive	good	inactive	good	inactive	good	inactive	good
Thyroid Receptor Beta effect	inactive	good	inactive	good	inactive	good	inactive	good	inactive	good

Table S12. Predicted oral LD50 and NOAEL in rats.

Substance	LD_{50} (mg/kg bw)	NOAEL (mg/kg bw per day)
CBA	1895	92.3
CDBA	2852	6.5
MeCDBA	1661	3.6
FBA	1683	116.2
FDBA	1641	8.2

Table S13. Prediction based on read-across analysis according to the OECD QSAR toolbox.

Substance	Genetic toxicity	Carcinogenicity	LD_{50} (mg/kg bw)
CBA	neg	neg	1480
CDBA	neg	neg	938
MeCDBA	neg	neg	214
FBA	neg	neg	1700
FDBA	neg	neg	1160

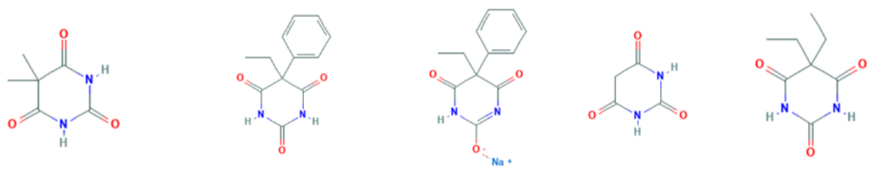

	Name	5,5-Dimethylbarbituric acid	Phenobarbital	Phenobarbital sodium	Barbituric acid	Barbital
	CAS	24448-94-0	50-06-6	57-30-7	67-52-7	57-44-3
Genetic toxicity	in vitro bacterial reverse mutation assay	-	negative	negative	negative	-
	in vitro mammalian cell micronucleus test	-	negative	-	-	-
	in vitro mammalian chromosome aberration test	-	negative	-	-	-
	in vitro mammalian cell gene mutation assay	-	positive	-	-	-
	in vitro micronucleus assay	-	negative	-	-	-
	in vivo mammalian chromosome aberration test	-	negative	-	-	-
	in vivo micronucleus assay	-	equivocal	-	-	positive
	in vivo transgenic rodent mutation	-	negative	-	-	-
	in vivo unscheduled DNA synthesis	-	negative	-	-	-
	Carcinogenicity	-<br>-<br>negative (rat)<br>-	negative (hamster)<br>positive (mouse)<br>negative (rat)<br>positive (rodents - undefined)	-<br>-<br>-<br>-	-<br>-<br>-<br>-	-<br>-<br>negative (rat)<br>-
	Oral LD_{50} (rats)	-	162 mg/kg	660 mg/kg *	5000 mg/kg<br>> 2000 mg/kg	600 mg/kg (mouse) *

Fig. S57: Compounds gathered for the read-across analysis. The OECD QSAR Toolbox was used to retrieve experimental data. Asterisks indicate that the information was not directly retrieved by the Toolbox but added by the authors (information retrieved using the PubChem database).

M. References

1. M. Mukhopadhyay, D. Banerjee and S. Mukherjee, *J. Phys. Chem. A*, 2006, **110**, 12743-12751.
2. T. T. Abiola, N. d. N. Rodrigues, C. Ho, D. J. L. Coxon, M. D. Horbury, J. M. Toldo, M. T. do Casal, B. Rioux, C. Peyrot, M. M. Mention, P. Balaguer, M. Barbatti, F. Allais and V. G. Stavros, *J. Phys. Chem. Lett.*, 2021, **12**, 337-344.
3. J. Owrutsky, D. Raftery and R. Hochstrasser, *Annu. Rev. Phys. Chem.*, 1994, **45**, 519-555.
4. X. Zhao, F. Ji, Y. Liang, P. Li, Y. Jia, X. Feng, Y. Sun, Y. Shi, L. Zhu and G. Zhao, *J. Lumin.*, 2020, **223**, 117228.
5. M. A. P. Turner, R. J. Turner, M. D. Horbury, N. D. M. Hine and V. G. Stavros, *J. Chem. Phys.*, 2019, **151**, 084305.
6. A. L. Whittock, M. A. P. Turner, D. J. L. Coxon, J. M. Woolley, M. D. Horbury and V. G. Stavros, *Front. Chem.*, 2020, **8**, 574038.
7. T. Kumpulainen, B. Lang, A. Rosspeintner and E. Vauthey, *Chem. Rev.*, 2017, **117**, 10826-10939.
8. M. Caricato, *J. Chem. Phys.*, 2013, **139**, 044116.
9. C. M. Marian, A. Heil and M. Kleinschmidt, *Wires Comput. Mol. Sci.*, 2019, **9**, e1394.
10. S. Grimme and M. Waletzke, *J. Chem. Phys.*, 1999, **111**, 5645-5655.
11. A. D. Becke, *Phys. Rev. A.*, 1988, **38**, 3098-3100.
12. C. Lee, W. Yang and R. G. Parr, *Phys. Rev. B.*, 1988, **37**, 785-789.
13. A. D. Becke, *J. Chem. Phys.*, 1993, **98**, 1372-1377.
14. M. Gerenkamp, PhD thesis, University of Muenster, 2005.
15. F. Weigend and R. Ahlrichs, *Physical Chemistry Chemical Physics*, 2005, **7**, 3297-3305.
16. S. G. Balasubramani, G. P. Chen, S. Coriani, M. Diedenhofen, M. S. Frank, Y. J. Franzke, F. Furche, R. Grotjahn, M. E. Harding and C. Hättig, *J. Chem. Phys.*, 2020, **152**, 184107.
